# Artificial Intelligence in the Discovery and Design of Ionic Liquids for Thermal Energy Applications

**DOI:** 10.1021/acs.jcim.6c02407

**Published:** 2026-08-28

**Authors:** Pawan Chhipa, Zachariah S. Baird, Dinis O. Abranches, Oliver Järvik

**Affiliations:** † Department of Energy Technology, 54561 Tallinn University of Technology, Ehitajate Tee 5, Tallinn 19086, Estonia; ‡ CICECO-Aveiro Institute of Materials, Department of Chemistry, 426216 University of Aveiro, Aveiro 3810-193, Portugal

**Keywords:** Ionic Liquids, Phase Change Materials, Thermal Energy Storage, Machine Learning, Enthalpy of Fusion, Applicability Domain

## Abstract

This review addresses a critical need in the emerging intersection of digital chemistry and material properties by critically evaluating the current capabilities and future prospects of applying machine learning (ML) to ionic liquids (ILs) for thermal energy storage (TES) applications. We examine available data sets for key thermophysical properties relevant to TES, including thermal conductivity, heat capacity, density, viscosity, surface tension, melting point, enthalpy, CO_2_ solubility, and toxicity. Particular emphasis is placed on chemical space coverage, biases, and practical limitations. We compare different modeling approaches, spanning from conventional QSPR/QSAR and group-contribution methods to contemporary ML approaches, including kernel models, ensemble methods, graph neural networks (GNNs), and deep learning architectures. The impact of molecular representations and descriptor choices and validation strategies in determining model robustness and transferability is examined. To structure this assessment, we introduce a three-tier property-selection framework for IL-based TES and apply a six-criterion evaluation checklist consistently across all nine reviewed properties. The analysis is supported by literature-summary tables that consolidate data sets, chemical space coverage, modeling approaches, and validation practices across different property domains. Finally, we outline recommendations for data set standardization, open sharing of data and code, and the establishment of benchmark validation protocols to improve reproducibility and accelerate the discovery of ILs for TES applications.

## Graphical abstract

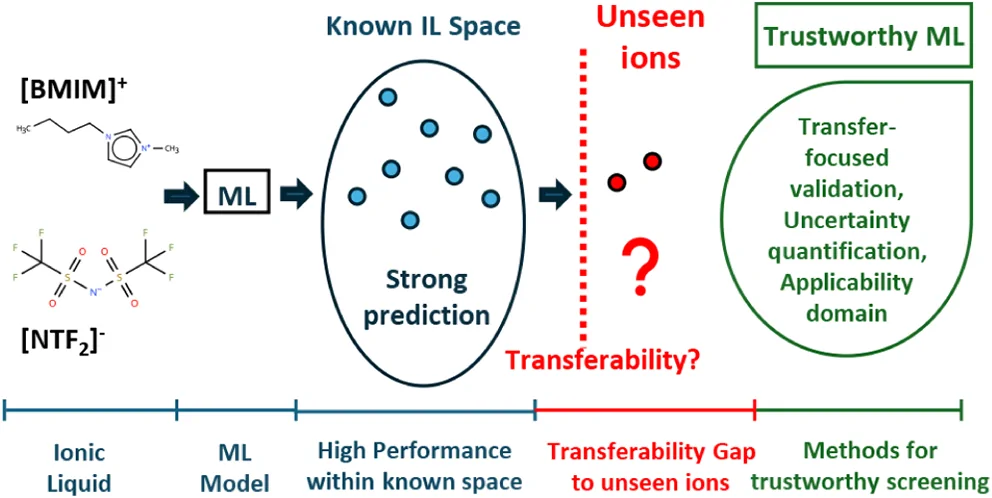

## 1 Introduction

Thermal energy storage (TES) enables the temporal decoupling of heat supply and demand by storing energy in the form of sensible, latent, or thermochemical heat.^1^ Among these, latent heat thermal energy storage (LHTES) offers particularly high energy density within a narrow temperature range, a feature that is often critical for system efficiency and performance.^2^ In conventional classifications, sensible TES stores energy through temperature change in a medium, LHTES uses solid–liquid phase transitions at defined melting temperatures with an associated enthalpy of fusion, and thermochemical TES relies on reversible chemical reactions and their associated enthalpies.^2^ LHTES systems may also rely on solid–solid transitions.^3^

Phase change materials (PCMs) are central to improving the efficiency of TES systems.^4^ During the charging phase, PCMs absorb heat as they melt; during discharging, they release stored energy upon solidification.^5^ Conventional PCMs face several challenges. Paraffins are flammable and have low thermal conductivity.^2^ Inorganic salt hydrates are prone to supercooling and phase separation, compromising long-term stability.^6^ High-temperature chloride-based molten salts often suffer from low heat capacity, making them unsuitable for large-scale TES.^7^ In addition, traditional PCMs often have low energy storage density and can be corrosive or chemically unstable during phase transition.^8^ These persistent challenges motivate the search for alternative compounds for TES applications.

Ionic liquids (ILs) have emerged as a promising class of PCM candidates for TES. Unlike conventional molecular solvents, ILs exist as liquid salts at or near room temperature. They offer several advantageous properties like negligible vapor pressure, usually exceptional thermal stability (up to 300–400 °C), and tunability, i.e., the physicochemical properties of ILs can be modulated through systematic variation of cation and anion structures. Originally defined as salts with melting points below 100 °C that consist solely of ions. They are effectively room-temperature molten salts whose melting point can be tuned by selecting different cations and anions.^9^

Desirable thermophysical properties for PCMs to be used in efficient TES systems include specific melting points, high enthalpies of fusion, adequate thermal conductivity, manageable viscosity for heat transfer, minimal supercooling, and long-term cycling stability. The chemical designability of ILs allows precise tuning of all of those properties.^10^ However, the theoretical chemical space of ILs expands to over 10^14^ unique cation–anion combinations.^11^ This immense diversity introduces significant complexity for traditional screening approaches.^11^ To date, the discovery of new ILs typically relies on modifying well-known cation–anion pairs or exploring new combinations through trial-and-error experimentation, which is both time-consuming and costly.^12^ Thus, experimental screening of even a small fraction of the theoretical IL chemical space is prohibitively resource intensive, with only a limited subset of the IL universe having been explored experimentally. Beyond neat ion pairs, the design space further expands to polymerized ILs, large organic ions, inorganic components, and mixtures. Deep eutectic solvents (DESs), while not ILs in the strict sense (they typically combine a salt with a hydrogen-bond donor rather than consisting solely of ions), are included in this review because the same ML approaches and descriptor frameworks apply. These extended families broaden the range of tunable interactions and open additional routes for tailoring materials suited for TES.

A possible solution for addressing the exploration of a virtually infinite number of viable ILs lies in predictive modeling and molecular simulations. The earliest efforts in high-throughput property prediction, also referred to as virtual screening or in silico prediction,^13^ date back to the late 1960s and early 1970s.^14^ Since then, these computational approaches have been applied across a wide range of scientific and engineering domains. In practice, structure–property relationships, group-contribution (GC) methods, equations of state, and COnductor-like Screening MOdel (COSMO) based approaches offer rapid estimations of physicochemical properties. However, they face several important limitations when applied to the design of ILs for TES. Empirical correlations and GC methods^15–17^ often lack predictive accuracy when extrapolated to novel chemical structures. Equations of state require fitting parameters, usually unavailable for many ILs and mixtures. COSMO-based models (e.g., COSMO-RS) were parametrized for neutral molecular systems and can predict certain IL properties with reasonable accuracy; however, they struggle with long-range Coulombic interactions in ionic media, which limits their reliability for properties governed by cooperative ion–ion effects. As discussed in Section 2.1, COSMO-derived descriptors remain highly effective when used as inputs to ML models rather than as direct predictors.^18–21^

Computational simulations, such as molecular dynamics (MD), Monte Carlo, and quantum chemistry calculations, can probe ILs at a molecular level, revealing detailed information about their structure and properties. For example, when carefully parametrized, MD simulations can predict transport properties, phase behavior, and interfacial characteristics that are critical for optimizing performance in TES applications.^22^ However, the choice of force-field, particularly parameters such as partial charges and the treatment (or not) of polarizability, has a strong influence on simulation accuracy.^23^ To extend simulations to longer time and length scales, coarse-grained models are employed. These models simplify molecular complexity by mapping multiple atoms into single beads, albeit at the cost of chemical detail. Other techniques like Monte Carlo methods provide alternative pathways for sampling system states and calculating thermodynamic properties, often with different computational trade-offs.^24^ All in all, extremely long simulation times are required for the convergence of the transport and dynamical properties of ILs, which pushes the computational costs beyond the limits of traditional MD approaches for high accuracy.^25^ Recent ML interatomic potentials (MLIPs) offer a possible solution to the accuracy-cost trade-off by learning quantum-chemical potential-energy surfaces and applying them to longer molecular simulations. However, their reliability is still limited by the quality and representativeness of the underlying reference data.

Artificial Intelligence (AI) and ML have transformed chemistry, materials science, and related disciplines, replacing traditional trial-and-error approaches with data-driven discovery.^26^ By learning directly from experimental data, augmented where beneficial by simulation-derived features or physics-based descriptors, ML models have the potential to deliver rapid, accurate predictions across the vast design space of ILs. Once trained, ML models can typically generate predictions in seconds. Even when accounting for training time, ML approaches generally have lower computational costs than simulations. If suitable data is available, an ML model covering a wide range of compounds can often be developed relatively quickly. Moreover, certain ML approaches can quantify predictive uncertainty (i.e., inform the user the type of data most beneficial to improve the accuracy of the model), guiding experimental data acquisition and accelerating materials discovery. The convergence of ML and IL research thus creates opportunities for TES innovation. In this context, the ability to rapidly identify ILs with specific properties is essential, and ML provides the tools to navigate this enormous design space with potentially unprecedented speed and precision.

Sitting at the interface of AI and materials science, this review examines how data-driven methods are being applied to accelerate IL discovery, with particular emphasis on TES-relevant properties. We start by outlining the foundations of ML models and molecular representations, both in general terms and specific to IL discovery. Then an extensive survey of property-specific applications across thermal, transport, and phase-transition behavior of ILs is presented. Finally, this review concludes by identifying current limitations, emerging directions, and opportunities for integrating physics-based insight with modern ML to guide future IL design. A central finding emerges throughout: the field’s limiting factor is not algorithmic sophistication, but rather, the absence of standardized, chemically diverse, and externally validated benchmarks. While most published models achieve high apparent accuracy within narrow chemical domains, validation is rarely designed to test transfer to unseen cation–anion families. Progress toward genuine IL discovery for TES will require transfer-focused validation (leave-one-ion or leave-one-family out rather than random splits), explicit applicability domains, uncertainty quantification, and the open release of data sets and trained models.

## 2 Machine Learning

ML encompasses methods ranging from linear models such as multiple linear regression (MLR) and support vector machines (SVMs) to nonlinear approaches including kernel methods, ensemble tree models, and neural networks (NNs), all of which learn relationships between molecular inputs and target properties from data. For ILs, the critical challenges are not algorithmic but data-specific: how to represent a two-component ionic system, how to validate models intended for discovery rather than interpolation, and how to define the domain in which predictions are reliable. These IL-specific issues, which shape every property section that follows, are discussed below alongside the standard modeling workflow.^27–30^

### 2.1 Molecular Representations

A critical decision in ML model development is the selection of input data. ML models are, after all, mathematical models that require numerical inputs to make predictions. However, molecular structures are not numerical in nature and thus must be converted somehow. Figure 1 illustrates representative molecular representations for the same ionic liquid, including string based encodings, molecular graphs, fixed length descriptor vectors, and COSMO RS derived σ representations. This process is often called molecular featurization, and can involve something as simple as SMILES (Simplified Molecular Input Line Entry System)^31,32^ or SELFIES (SELF-referencIng Embedded Strings)^33^ or InChI^34^ strings. However, recent studies increasingly rely on the usage of molecular descriptors richer in chemical/molecular information.^13^ Molecular descriptors can range from simple counts of atoms or functional groups; 2D descriptors, such as graphs or topological descriptors; or 3D descriptors, such as geometrical descriptors.^35^ Thousands of different molecular descriptors can be calculated using specialized software.^36^ For example, Dragon^35^ calculates over 5,000 descriptors, while the Chemistry Development Kit (CDK)^37^ offers a broad suite of descriptor calculations.^37^ In practice, multiple descriptors are often combined to capture diverse molecular characteristics and improve predictive accuracy.^35^ Some models even rely on the exhaustive calculation of molecular descriptors to identify optimal combinations.^13^ Beyond traditional descriptors, more complex representations of molecules, such as graphs or convolutional neural networks (CNNs), have been employed to retain detailed information about the local chemical environment.^13,38^ The choice of input data is important because the set of features used limits the available chemical/molecular information for the model.^13^

**Figure 1:**
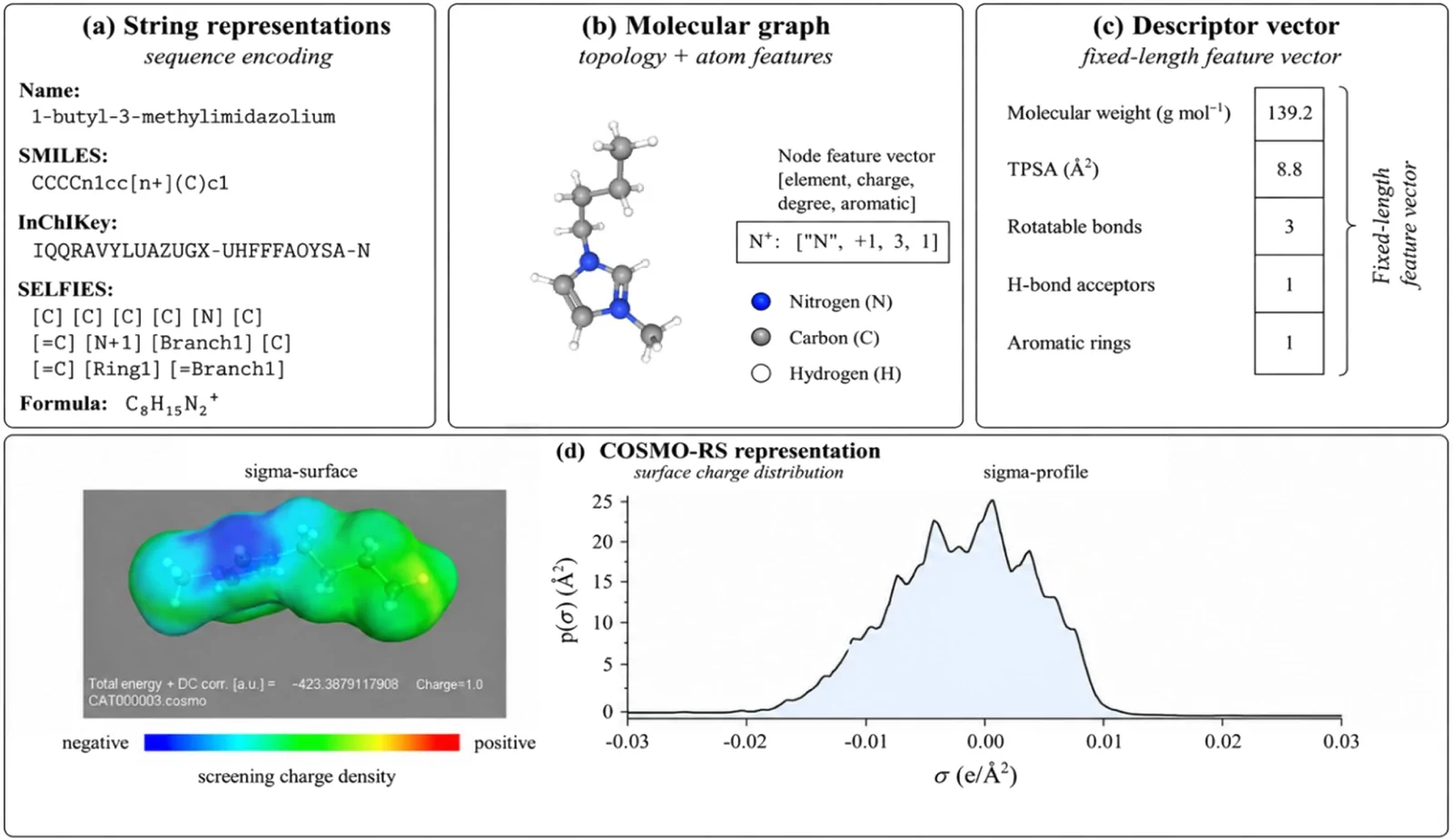
Representative molecular encodings of the 1-butyl-3-methylimidazolium cation [BMIM]^+^. (a) String representations, including SMILES, InChIKey, SELFIES, and molecular formula. (b) Molecular graph representation, in which atoms are treated as nodes and bonds as edges, with associated atomic features. (c) Fixed-length molecular descriptor vector containing representative physicochemical and structural descriptors. (d) COSMO-RS representation, showing the σ-surface and corresponding σ-profile that describe the molecular surface charge-density distribution. Together, these representations encode complementary structural, physicochemical, and electronic information.

Recent advances in ML increasingly favor models that learn molecular representations directly from structure to eliminate the need for manually engineered descriptors. One of the major developments in this area is the rise of graph neural networks (GNNs) for molecular property prediction.^39–41^ These models treat molecular structures as mathematical graphs, where atoms become nodes and bonds become edges.^41^ Common examples include message-passing neural networks (MPNN)^42^ and related variants such as gated-graph neural networks (GG-NN),^43^ graph attention networks (GAT),^44^ GraphSAGE,^45^ graph convolutional networks (GCN),^46^ and hierarchical intermessage passing^47^ models. FragNet^48^ is one such GNN. It maintains good predictive accuracy, shows how specific atom bonds and fragments influence the output providing a clearer link between structure and prediction.^48,49^ Progress has also been made in combining different data types into unified models. Modern ML models can now work with 2D molecular structures, 3D geometries, spectroscopic data, and even textual descriptions within one model. This multimodal approach provides richer and more comprehensive representation of molecular systems compared to earlier models that relied solely on limited sets of predefined descriptors.^50,51^

The introduction of sigma profile^21^ as an intermediate representation derived from quantum chemical calculations has recently redefined digital molecular spaces for ML applications. This descriptor stems from the Conductor-like Screening Model for Real Solvents (COSMO-RS) methodology.^52^ The sigma profile represents the surface charge density distribution of the molecule, derived from the distribution of polarization charge densities (σ) on the molecular surface.^19^ This representation is crucial because it characterizes the chemical nature of both the cation and the anion structures of the IL. This descriptor encapsulates electronic structure thereby providing a physics-informed feature set that drastically reduces reliance on purely empirical descriptors. It offers a system-independent, low-dimensional, yet information-rich representation of molecules. Abranches et al.^53^ established a continuous descriptor landscape using σ-profiles, enabling Gaussian Process (GP) models to interpolate and optimize across broad molecular classes, even with limited data availability. This descriptor-based usage differs from direct COSMO-RS property prediction. When σ-profiles serve as inputs to an ML model, the model learns to correct for the systematic limitations of the COSMO-RS framework, including its treatment of long-range ionic interactions, rather than inheriting them. This distinction extends to residual-learning approaches, where ML models are trained on the difference between COSMO-RS predictions and experimental values, effectively using COSMO-RS as a physics-based prior that the data-driven component refines (see, e.g., Qin et al.^18^ for CO_2_ solubility in Section 5).

The choice of molecular descriptors can be complemented by physics-informed NNs, which integrate physical constraints into training to ensure thermodynamic consistency in predictions.^54^ Related coarse-graining approaches also embed statistical-mechanics structure in the learning problem; Huang et al.^55^ introduced Statistical-Physics-Informed Neural Networks (Stat-PINNs), which use short-time particle simulation data to identify the thermodynamic potential and dissipative operator governing the macroscopic dynamics. The basic idea is to integrate physical laws directly into the learning process, ensuring that predictions remain consistent from a thermodynamic perspective.^54^ A compelling example is ThermoLearn, which predicts Gibbs free energy, total energy, and entropy all at once, while enforcing consistency with the Gibbs equation.^54,55^

### 2.2 Model Training and Evaluation

A critical step in training an ML model is obtaining high-quality training data.^56^ Since ML algorithms learn patterns directly from the data, the quality, distribution, and accuracy of that data fundamentally shape model performance. Errors or biases in the training data can lead to an inaccurate model.^57^ Ideally, an ML training data set should be evenly distributed across the target prediction space and free of outliers. However, real-world data sets, particularly those related to the properties of materials and molecules, are often sparse, clustered, and noisy. A dramatic increase in the volume of published experimental data over recent years has also raised concerns about data quality. In fact, studies have found approximately one-third of the recent scientific articles reporting thermophysical data contain substantial issues.^58^ It means there is an increasing demand for critical evaluation of experimental data to ensure its quality.

Assembling sufficient data is particularly challenging for properties that are difficult to measure. Even benchmark databases in fields like drug discovery have faced criticism for limited chemical diversity and high internal correlation.^59^ Much of ML research focuses on data-rich environments, such as automatic translation, image recognition, and recommendation systems, where large, well-structured data sets are readily available. Consequently, many ML methods are not inherently designed to perform well in situations where data is lacking. For these reasons, acquiring high-quality training data is one of the most important and challenging tasks that needs to be addressed, and computational simulations can help generate additional training data for ML models.^30^ This synergy is particularly valuable for ILs, where the complex interactions between different ions create complex behaviors that benefit from both physical understanding and data-driven insights.

To aid data collection, the possibility of automatically creating databases from scientific literature is being extensively studied. One example is ChemDataExtractor,^60^ which Zhao et al.^61^ used to build a database containing 49,076 refractive index and 60,804 dielectric constant data records covering 11,054 unique chemicals. A deep learning method presented by Staker et al.^62^ uses ML to extract molecular structures from journal articles and patents. The authors point out that large amounts of data reside in public literature, but they are often inaccessible to computers due to formatting and structure limitations.

Although recent language models can substantially improve the scalability and accuracy of scientific data extraction,^63^ automatically generated databases remain susceptible to errors arising from ambiguous property definitions, chemical nomenclature, units, and experimental context. Athar et al.^64^ demonstrated that related but physically distinct thermoelectric quantities and material compositions could be incorrectly represented during database construction. Analogous risks apply to IL data, where subscripts, phase information, composition, and measurement conditions are necessary to distinguish related thermophysical properties.^65^ Automated extraction should therefore be combined with property-specific consistency checks, source-provenance tracking, and targeted verification against the primary literature before the resulting data are used for machine-learning model development.

Before training, input data are typically scaled and transformed, and features are selected to reduce redundancy.^66^ Because these preprocessing choices can substantially change reported accuracy, studies should report the specific steps and the final descriptor set used for model fitting. Model training itself involves fitting parameters to minimize prediction errors, a process prone to overfitting if not properly regularized and validated. Proper separation between training, validation, and testing data is essential for unbiased performance estimates, and studies should report their split strategy and evaluation protocol explicitly.^67^

US National Institute of Standards and Technology (NIST) has developed a new dynamic evaluation framework, which includes ML-based property prediction methods.^23,68^ The ML models are used to help assess the validity of newly reported experimental data, especially in areas where no other experimental data exists for comparison.^68^ NIST has compared their ML models to commonly used group contribution methods, and stated that the results from ML were “far superior”.^23,69^ Another interesting feature of the NIST ML models is the incorporation of training data uncertainty into the prediction process. This is an aspect often overlooked.^23^ Given that measuring thermodynamic and physical properties is often time-consuming and resource intensive, many research groups have focused on developing ML models to predict such properties. Examples of such efforts can be found in the literature.^70–76^

There has also been growing interest in understanding why models make certain predictions, particularly identifying which molecular features drive property values.^77^ A major trend of 2025 is the use of interpretability tools like SHapley Additive exPlanations (SHAP)^78^ and layer-wise relevance propagation (LRP) for chemical ML models. Transformer models and GNNs, which can be black boxes, are increasingly explained using SHAP to identify which molecular substructures most influence model predictions. For instance, a 2025 advance documented by Highlights in Science, Engineering and Technology employed SHAP to decompose CO_2_ solubility prediction models, correlating key anion and cation features with model outputs providing computational chemists with actionable structure–activity insights.^78^

### 2.3 Other Classes of ML

Stochastic ML approaches, such as active learning and Bayesian optimization,^53,79^ have become another useful tool in AI workflows. Rather than collecting data randomly, active learning algorithms strategically identify the most informative experiments to perform next. This can dramatically reduce the amount of data required to achieve accurate predictions, which is especially valuable when measurements are expensive or time-consuming.^80^

Transfer learning is another powerful approach to data-efficient discovery. By building on models pretrained on large molecular databases, such as ZINC (with >750 million compounds) and ChEMBL^81^ (with more than 2 million bioactive molecules), GDB-17,^82^ it is possible to fine-tune these models on smaller target data sets. This often yields superior performance compared to training models from scratch, especially in property prediction tasks where related data exists but target-specific measurements are scarce.^83^ Transfer learning helps to bridge the sim-to-real gap by pretraining on computational data sets (e.g., quantum-chemical or force-field properties) and fine-tuning on experimental targets, thus helping to generalize better across different data sources and improving the predictive accuracy.^83^ Furthermore, transfer learning is a proven strategy to improve learned representations. Graph encoders pretrained on large molecular data sets using self-supervised objectives, such as masked-atom/bond prediction or contrastive learning, can be fine-tuned for specific property prediction tasks. These pretrained models often match or outperform models trained from scratch, while requiring far less labeled data.

Large language models (LLMs) have entered chemistry, with systems such as ChemDFM^84^ trained on chemical literature to interact with chemical knowledge through natural language. Autonomous research systems such as Coscientist and the A-Lab combine LLMs or ML surrogates with robotic platforms to design and execute experiments without human intervention. No equivalent autonomous campaign targeting IL property measurement or TES screening has been reported, but the infrastructure for closed-loop IL discovery is maturing.^85,86^

Modern ML systems can also quantify prediction confidence, identifying which molecular substructures or conditions contribute most to prediction error. For IL screening, such uncertainty estimates could guide which candidate chemistries merit experimental measurement, a capability that remains underused in the studies reviewed in Section 5.^87^

Several ready-to-use ML tools require no expertise in model development. The US Environmental Protection Agency’s toxicity estimation tool, for instance, predicts multiple toxicity end points and physical properties from chemical structure, offering a low-barrier entry point for preliminary IL safety screening.^88^

## 3 Properties Governing IL Selection for Thermal Energy Storage

The thermophysical properties relevant to IL-based PCMs do not all serve the same role during materials selection. Some determine whether an IL can function as a PCM, others determine whether it can operate within a TES system, while others constrain practical deployment. Organizing these properties according to their function provides a clearer basis for evaluating both the current ML literature and the remaining research gaps.

### 3.1 Tier 1. Storage Performance

As shown in Figure 2, Tier 1 includes melting point (T_m_), enthalpy of fusion (ΔH_fus_), heat capacity (c_p_), thermal conductivity (λ), volumetric latent heat (ρΔH_fus_), supercooling tendency (ΔT_sc_), and thermal diffusivity (α). These properties determine whether an IL can store and release heat within the required operating temperature window, and how quickly that energy can be transferred in and out of the material. Melting point and enthalpy of fusion define the latent heat storage process, while heat capacity, thermal conductivity, and thermal diffusivity influence heat storage and transfer. Volumetric latent heat and supercooling are also important performance metrics but have received little attention in IL-specific ML studies.

**Figure 2:**
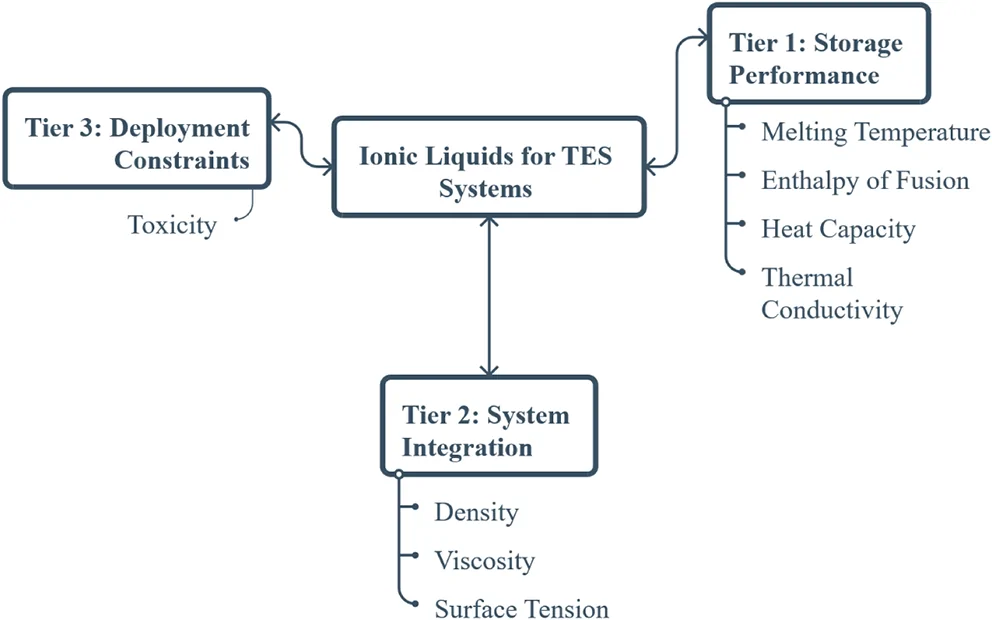
Three-tier framework for classifying the properties governing IL selection for TES. The tiers distinguish properties related to storage performance, system integration, and practical deployment constraints.

### 3.2 Tier 2. System Integration

Tier 2 includes density (ρ), viscosity (η), surface tension (γ), and cycling stability. These properties do not determine the intrinsic heat-storage capability of an IL but govern whether it can be implemented in a practical TES system. Density and viscosity influence flow behavior and pumping requirements, surface tension affects interfacial behavior, and cycling stability determines long-term performance during repeated melting and solidification.

### 3.3 Tier 3. Deployment Constraints

Tier 3 includes toxicity, thermal decomposition temperature (T_dec_), corrosion behavior, and cost or synthesis feasibility. These properties determine whether an IL with suitable performance can be deployed safely and economically. While toxicity has received increasing attention in ML studies, thermal decomposition, corrosion, and cost remain largely unexplored.

CO_2_ solubility is not included within the three-tier framework because it is not a TES selection property. It is retained as a methodological case study. Compared with most TES-related properties, CO_2_-solubility prediction has benefited from larger data sets, broader model comparisons, and more rigorous validation strategies. These studies provide useful methodological guidance for developing ML models for TES properties.

Current ML research is concentrated mainly on Tier 2 properties, particularly density and viscosity. By contrast, the Tier 1 properties that most directly determine PCM performance remain underrepresented. Enthalpy of fusion has only one dedicated ML study, while thermal conductivity has less than ten. This imbalance highlights one of the principal gaps in the current IL-ML literature and provides clear direction for future model development.

## 4 ML in IL Discovery

The intersection of ML and ILs has seen a remarkable growth since 2015. Figure 3 shows the yearly distribution of publications from 2010 to 2025. After 2020 there was a sharp rise in ML-focused IL studies, while traditional QSAR/QSPR studies remain comparatively steady, fluctuating around 20 to 50 papers per year without a comparable growth trend. This relationship indicates a growing interest and shift toward ML over conventional QSAR methods in IL research. The number of publications on ILs increased over time until 2020, reaching nearly 8000 papers per year. As shown by the publication trends in the Web of Science database, the application of ML to ILs is a rapidly expanding field. As of December 2025, 832 articles have been indexed under the broad topic of ML models applied to ILs.

**Figure 3:**
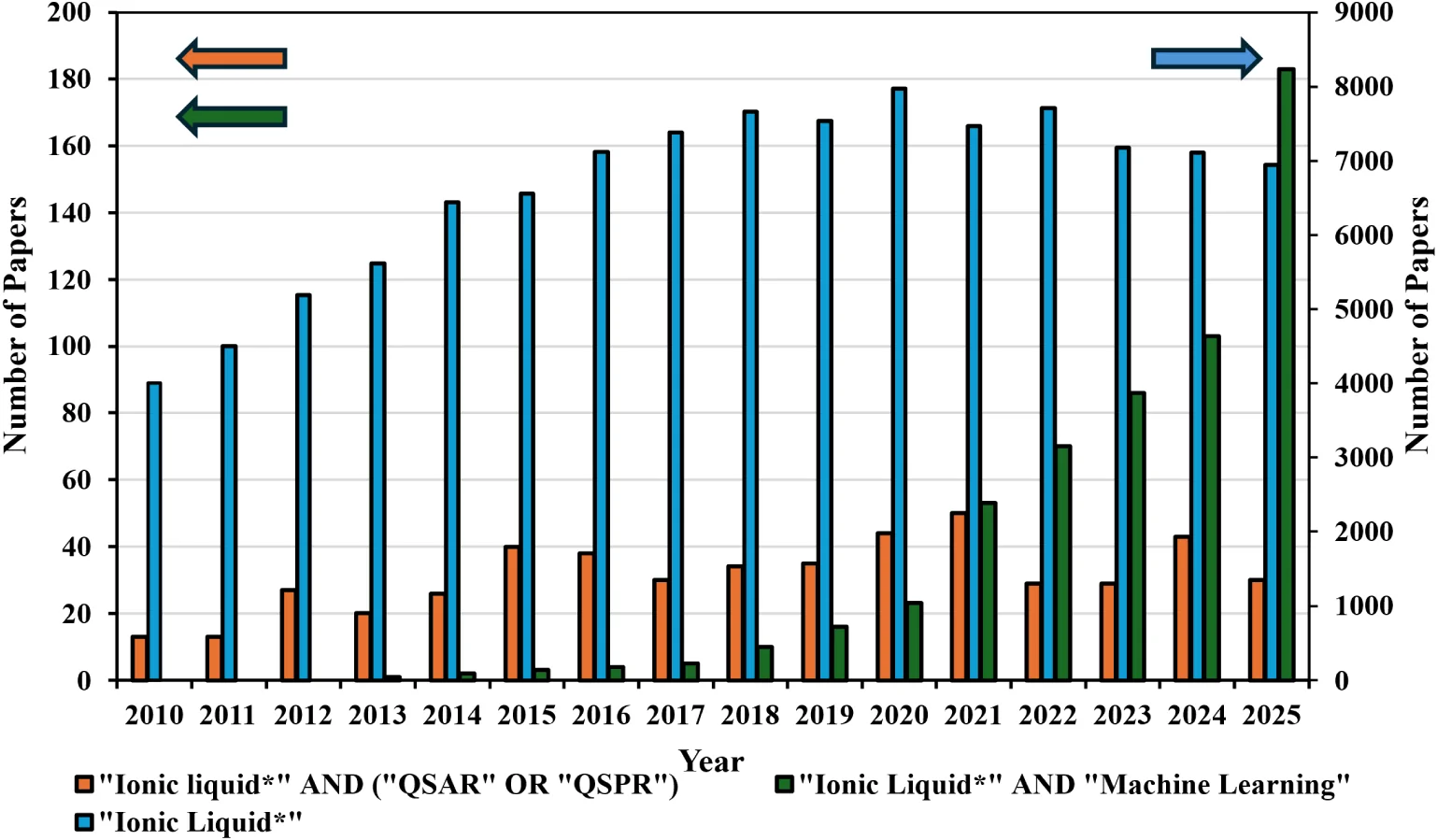
Annual publication trends retrieved from the Web of Science database using the queries included in the figure caption. The search term “Ionic Liquid*” is displayed using the secondary axis to highlight its larger publication volume relative to the other queries. Data taken as of 31–12–2025.

The bibliometric analysis in Figures 3–5 was conducted using the Web of Science Core Collection, with all records retrieved on 31 December 2025. Searches were restricted to the abstract field to reduce false positives, and wildcard truncation was used to capture term variants. For Figure 4, searches combined an IL term, model terms, and property-specific terms; the full search strings and property lists are provided in the Supporting Information. Records for Figures 3 and 4 were deduplicated and manually screened to retain only studies explicitly applying ML to IL property prediction. Figure 5 was based on raw abstract-level model-name searches without manual curation; therefore, papers may be counted under multiple model categories when more than one model term appears in the abstract.

**Figure 4:**
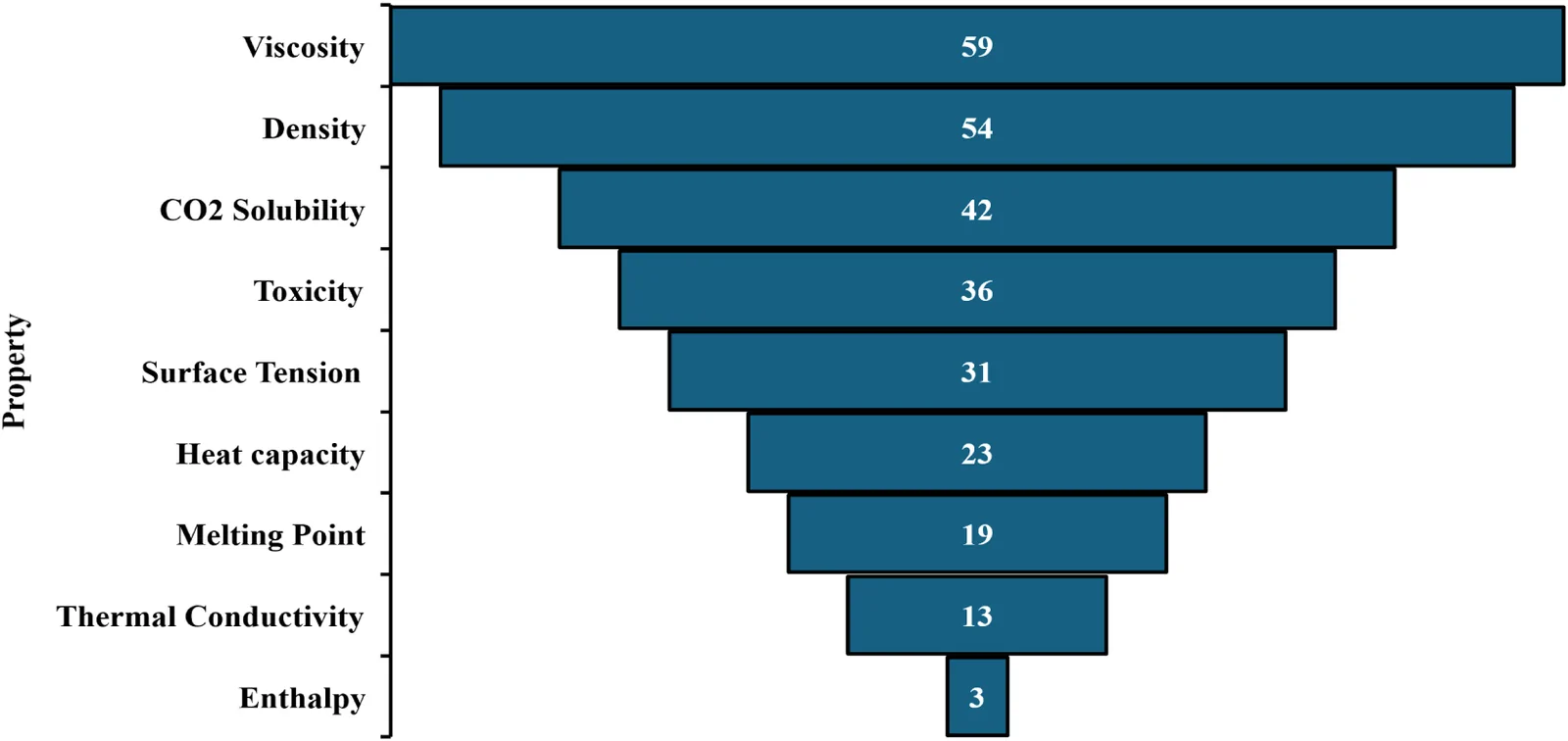
Number of publications retrieved from the Web of Science database for various properties using the search query: “Ionic liquid*” AND “<Models>”AND “<Property>”. See the explanation of “<Models>” and “<Property>” in Supplementary Information. Each bar represents the total number of articles published related to a specific physicochemical or thermodynamic property. Data taken as of 31-12-2025.

**Figure 5:**
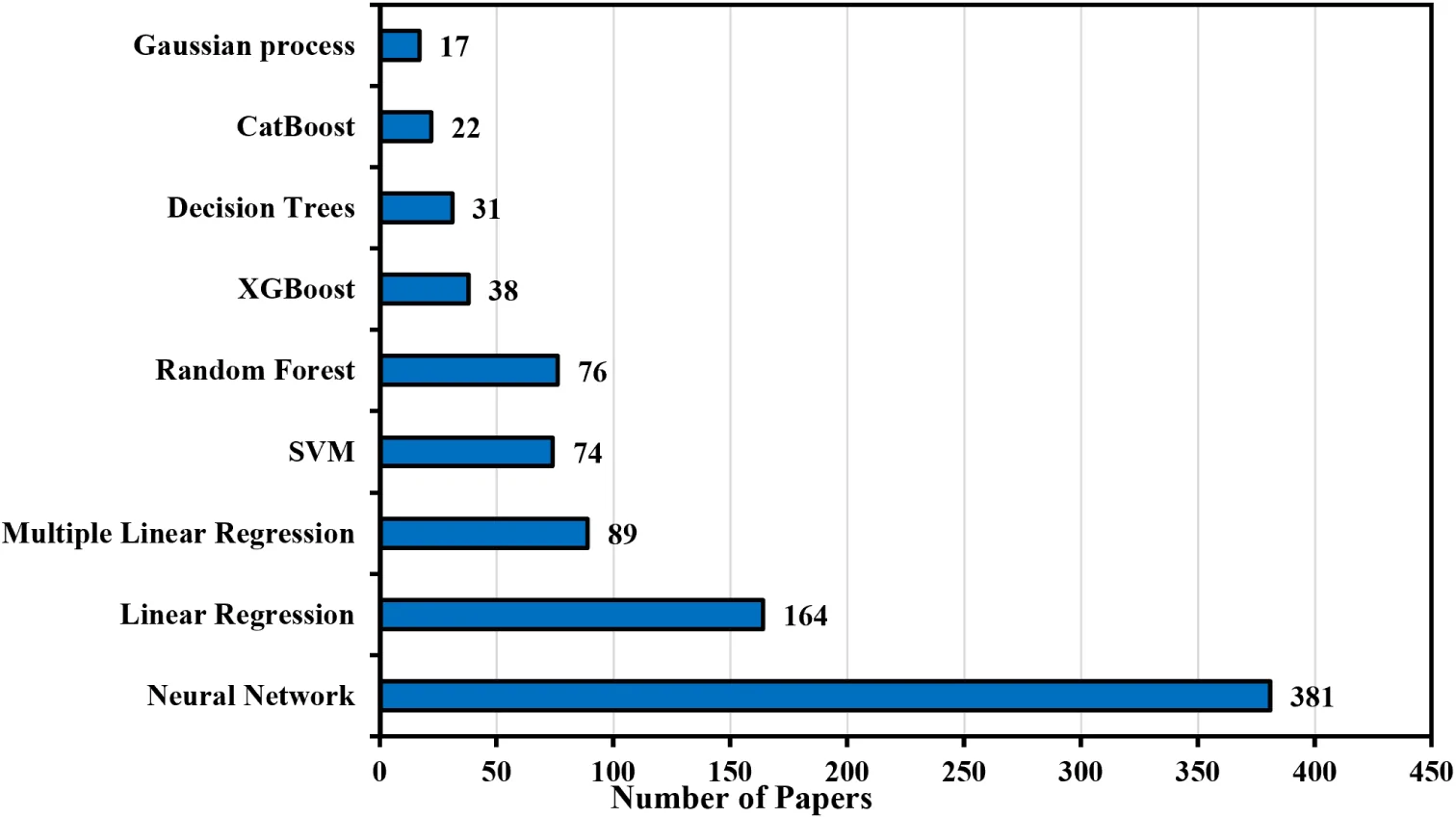
Number of publications retrieved from the Web of Science database for different ML models using the search query: “Ionic liquid*” AND “<Model Name>*”. Each bar represents the total number of articles associated with a specific ML algorithm applied to ionic liquid research. The plot reveals the popularity and adoption trends of various ML methods within the domain. Data taken as of 31–12–2025.

Several of the studies included in the statistical analysis of Figure 3 have applied ML to evaluate and predict IL properties. Figure 3 highlights the number of publications focusing on process-design-related properties, with particular emphasis on those relevant to TES, though not limited to this application. The explanation of how the information was collected is given in Supporting Information (SI). Interestingly, viscosity and density are the most extensively studied properties. CO_2_ solubility is well covered owing to interest in capture and separation applications and is tightly coupled to other thermophysical properties. Toxicity and surface tension are increasingly investigated as deployment and interfacial-performance constraints. Melting point and heat capacity, while less frequently modeled, are essential for phase-change behavior and stored energy per unit mass. Thermal conductivity and enthalpy remain the least studied targets and therefore constitute the primary data gaps for TES-focused screening and model development.

Similarly, Figure 5 illustrates the distribution of ML algorithms in IL research, derived from abstract-level searches using wildcards to capture all term variations (“Ionic liquid*” AND “<Model Name>*”). Unlike Figure 4, no additional manual curation was performed; the counts therefore reflect raw abstract matches. This provides a broader perspective on the ML methods most frequently reported in IL-related abstracts. NNs appear most frequently, whereas methods like XGBoost and GP demonstrate emerging, though still limited, adoption, indicating that the field has favored NN-based approaches. Publications may be counted under multiple models when abstracts mention more than one algorithm.

For ILs, a key representation decision is whether to describe the cation and anion separately or as an explicit ion pair. Separate cation and anion descriptors, such as individual σ-profiles, are computationally efficient and compositionally reusable because each ion descriptor can be combined with many counterions.^53^ They can still provide chemically rich information on polarity and surface-charge distribution, and the ML model can learn cation–anion combinations from paired inputs. However, fully explicit ion-pair descriptors may represent specific conformational and cooperative cation–anion effects more directly, particularly for properties strongly affected by ion pairing, such as melting point and viscosity. This comes at higher computational cost because ion-pair representations often require conformational sampling and scale less efficiently across the combinatorial IL space.^89^

A critical methodological concern for IL property prediction is data leakage. Most IL data sets contain repeated measurements of the same cation–anion pair at different temperatures, pressures, or compositions. Standard random train/test splits place data points from the same IL in both sets, inflating apparent accuracy without testing whether the model can predict properties of unseen chemistries. This is the single most common methodological flaw across the studies reviewed in this work. Discovery-oriented models require transfer-focused validation of increasing stringency: leave-one-cation-out, leave-one-anion-out, leave-one-family out, and external validation on independently measured ILs. Models should also define an explicit applicability domain: the region of chemical and condition space in which predictions are reliable. For ILs, this means specifying boundaries in terms of cation and anion families, temperature and pressure ranges, and descriptor-space coverage, rather than reporting only aggregate error metrics. Without a stated applicability domain, models risk silent extrapolation failure when applied to novel chemistries.^90,91^

Successful ML approaches for IL property prediction range from classical descriptor-based methods to NN-based models, including feed-forward architectures,^92^ graph-based representations, and transformer models^93^ pretrained on large chemical databases. These data-driven methods rely heavily on available data sets for training, yet IL property databases face unique challenges. Temperature-dependent measurement protocols, batch-to-batch synthesis variability, and trace water content can all significantly alter reported properties. While the ILThermo database is comprehensive,^94^ data availability is highly property-dependent, with substantial differences in the number of reported measurements across thermophysical properties, which constrains ML model development for sparsely populated targets.

Among NN-based approaches, Artificial Neural Networks (ANNs) have been widely applied in IL research, particularly in feed-forward and recurrent configurations.^92,95,96^ Across many studies, these models have been used to predict density, viscosity, thermal conductivity, and phase behavior using relatively compact descriptor sets. Comparative benchmarking by Racki et al.^78^ evaluated feed-forward, cascade, convolutional, and recurrent networks across several IL property prediction tasks. GNNs represent a more recent extension of neural models that encode ionic structures directly as molecular graphs. Baran et al.^97^ demonstrated that GNNs can robustly predict IL properties, even outperforming classical approaches when trained with noisy or incomplete data sets. Their work showed that combining cation and anion subgraphs via virtual ionic bonds improves representation and transferability across property domains.^97^ Similar trends have been reported in subsequent studies, where GNNs and transformer models trained on SMILES-encoded ions outperform generic descriptor-based regressions.^78^

Beyond architectural advances, transfer learning has been applied to mitigate data scarcity in IL property prediction. In this context, transfer learning applies knowledge encoded in models trained on large, general data sets and fine-tunes them for more specific, often data-scarce targets.^83^ ILBERT, introduced by Qiu et al. follow this paradigm^98^ by first being pretrained on millions of chemical structures and subsequently fine-tuned on IL property data sets. It achieved state-of-the-art predictions for 12 physicochemical properties of ILs and was experimentally validated on a set of novel electrolytes. This approach is especially valuable for newer IL chemistries, where experimental data is limited.^98^ Transfer learning now enables expanded predictive capabilities across highly diverse IL spaces.^83^ Gaussian Process Regression (GPR) has been successfully applied to predict properties such as CO_2_ solubility, viscosity, and other thermophysical characteristics. It provides precise predictions and statistical uncertainty estimates to guide further experimentation. For example, Zebida et al.^99^ reported that hybrid GPR LightGBM models trained on comprehensive IL data sets achieve superior performance in predicting CO_2_ solubility and conductivity, with uncertainty estimations directly informing the selection of optimal candidates.^99–101^ The same uncertainty-driven logic underlies active learning workflows that embed GP models in experimental campaigns on ILs and DES, where the model is updated iteratively and new experiments are chosen to be maximally informative.^102^

Bayesian optimization sits naturally within this stochastic ML family because it uses a probabilistic surrogate model, often a GP, to choose the next candidates to evaluate based on predicted performance and uncertainty. In IL design, this enables multiobjective searches over competing targets, such as high CO_2_ solubility and low viscosity, and helps map trade-offs in high-dimensional composition and structure spaces.^103^ Implementations in platforms such as Bohrium and EDBO+ illustrate how these surrogate-guided searches can accelerate screening and optimization workflows for IL applications. However, practical IL selection for TES requires simultaneous optimization of competing properties: high enthalpy of fusion against low viscosity, adequate thermal conductivity against acceptable toxicity. The vast majority of ML studies reviewed here address single properties in isolation and cannot capture these trade-offs. Multitask learning and Pareto-front screening could complement Bayesian approaches for coupled constraint screening, but such workflows remain rare in IL-for-TES work.^103,104^

### 4.1 Machine-Learning Interatomic Potentials and Simulation-Assisted Screening

Molecular simulations provide an important route for extending high-throughput property prediction beyond the region covered by experimental data.^24^ This is particularly relevant for ILs, where the cation–anion design space is extremely large, but the experimental coverage of several TES-relevant properties remains limited. In high-throughput screening, ML and molecular simulations are complementary: ML models can generate predictions rapidly once trained, but they usually require broad and representative training data, whereas simulations can provide information with little or no experimental input but at substantially higher computational cost.^24,57,106,107^ This complementarity has motivated hybrid workflows in which simulation-derived data are used to train ML models, or ML is used to accelerate the simulation step itself.^108–110^

Classical MD, Monte Carlo methods, and quantum-chemical calculations represent different compromises between physical detail and computational cost. Quantum-mechanical simulations provide direct electronic-structure information and are therefore attractive for describing ion–ion interactions, charge localization, and polarization effects. However, they are generally restricted to small systems and short time scales because of their computational cost.^56^ Classical MD can access larger systems and longer trajectories, but its reliability depends strongly on the chosen force field. In classical MD, the interactions between atoms and molecules are approximated by an interatomic potential energy function, and the quality of this approximation determines the quality of the resulting simulation.^23,111^ Monte Carlo methods provide an alternative sampling-based route for estimating thermodynamic properties, although they do not directly provide dynamical information.^24,112^ For ILs, these choices are especially important because density, viscosity, diffusion coefficients, thermal conductivity, melting behavior, and solid–liquid phase transitions are all sensitive to the balance between Coulombic interactions, dispersion forces, hydrogen bonding, conformational flexibility, and slow structural relaxation. The broader simulation literature also shows that simulation accuracy depends not only on the level of theory, but also on system size, equilibration time, sampling strategy, and the method used to calculate the target property.^24,56,114^

Machine-learning interatomic potentials (MLIPs) provide a possible route to reduce this accuracy–cost trade-off. Rather than relying on a fixed empirical force field, MLIPs learn the potential-energy surface from quantum-chemical reference data and then evaluate energies and forces at a cost closer to classical MD.^57^ In principle, this allows larger and longer simulations than ab initio MD while retaining a stronger connection to quantum-mechanical accuracy than conventional force fields. Early ML-based approaches to molecular simulation already showed that NNs and related models can approximate electronic-structure information more rapidly than direct quantum-chemical calculations, and this concept underlies modern MLIP development.^57,115^ For IL discovery, this is attractive because MLIP-based simulations could generate auxiliary data for properties where experiments are expensive, slow, or sparsely available, including diffusion coefficients, thermal conductivity, melting behavior, crystal packing, and enthalpy-related quantities.

The role of MLIPs in IL discovery is therefore not limited to producing final property predictions. They can also provide simulation-derived labels for downstream ML models, generate physically informed descriptors, and prioritize candidate ILs for experimental measurement. Simulation-derived training data have already been used in high-throughput property prediction, where large open databases of simulation results have supported ML model development.^108–110^ Simulations can also be used to calculate molecular descriptors that are then used as inputs to ML models, which is particularly useful when the target property is difficult to calculate directly from a full molecular simulation.^56,70,116,117^ In the context of ILs, a practical workflow would involve selecting representative cation–anion pairs, generating quantum-chemical reference structures across relevant conformations, temperatures, densities, and phases, fitting or fine-tuning an MLIP, and then using the resulting simulations to estimate properties or descriptors for a broader set of candidate ILs. Universal or foundation interatomic-potential models may further reduce the data requirement by providing transferable starting points that can be fine-tuned on IL-specific quantum-chemical data sets.

However, MLIP-derived data should not be treated as experimental ground truth. Their reliability is bounded by the quality, diversity, and chemical relevance of the underlying quantum-chemical training set. The performance of quantum-chemical calculations can depend on composition and atomistic configuration, and force-field or potential parameters are often optimized for specific classes of molecules.^118^ For ILs, basis-set effects, exchange-correlation functional choice, treatment of dispersion interactions, charge localization, finite-size effects, and incomplete conformational sampling can all introduce systematic errors. These uncertainties may propagate into downstream property models if simulated values are used directly as training labels. Thus, MLIP-assisted IL screening should be benchmarked against available experimental observables, including density, viscosity, diffusion coefficients, heat capacity, thermal conductivity, and phase-transition data. Experimental data remains essential for creating and validating prediction methods, and prediction methods are most useful when they also help identify which new measurements would add the most value.^23,56,119,120^

## 5 Property-by-Property Assessment of ML Models for Ionic Liquid Properties

This section provides a property-by-property review of IL research that uses ML and related computational methods. For each property, we synthesize findings from the published literature and, where available, summarize modeling approaches, algorithms, data sets, IL families, descriptors, and performance metrics in structured tables. We begin with thermal conductivity and enthalpy of fusion, two scientifically important yet comparatively underexplored targets in the ML-IL literature. Given the limited data sets available for these properties, the full summary tables are included in the main text (Tables 2 and 3) to show current coverage. The review then turns to properties that have been more extensively investigated, including density, viscosity, CO_2_ solubility, melting point, toxicity, heat capacity and surface tension; corresponding tables for these properties are provided in the Supporting Information (Tables S1–S7). To provide a consistent analytical lens across properties, Table 1 defines a six-criterion evaluation checklist applied throughout this section. For each property, we assess how the surveyed studies perform against these criteria and highlight systematic patterns of strength and weakness.

**Table 1:** Six-Criterion Evaluation Checklist for Assessing ML Studies on IL Property Prediction^a^

Criterion	Description
Split type	Random, leave-one-cation-out, leave-one-anion-out, leave-one-family out
External validation	Independent test set from a different laboratory, or external data source
Uncertainty quantification	Prediction intervals, ensemble variance, or related confidence measure reported
Applicability domain	Explicit domain boundaries or reliability indicators provided
Code/data availability	Published source code, trained model, raw or processed data sets
Experimental validation	ML-proposed candidates synthesized and measured

a This checklist provides a consistent framework for evaluating the methodological strengths and limitations of the studies reviewed in this section.

Each property-specific section now concludes with a synthesis paragraph that compares the reported methodologies, discusses the effect of descriptor selection, data set characteristics, validation strategy, and model performance, and identifies common methodological limitations across the literature instead of ending with a sequential summary of individual studies.

### 5.1 Thermal Conductivity

Thermal conductivity (λ) is classified as a Tier 1 property in the TES framework (Section 3): it governs the rate at which heat can be charged into or discharged from a PCM and is among the least-studied IL properties by ML methods, with fewer than ten published studies. Published ML studies for this property are summarized in Table 2. The table reports the IL families and measurement conditions represented in the underlying data sets, along with the modeling approach (ML model type and descriptor set), data set size, the property range covered, and the reported performance metrics. Where available, links to code repositories are included.

**Table 2:** Literature-Summary of Machine Learning Studies for the Prediction of Thermal Conductivity in Ionic Liquids

Reference	Families of Ionic Liquids	ML Models	Descriptor	Size of Data set	Thermal conductivity, W m^–1^ K^–1^	Range of Pressure	Range of Temperature, K	Performance	GitHub Link
Cao et al.^17^	46 ILs	MLR, RR, ANN, SVR, RF, and XGB	20 groups GC based	334 data points			273.15–390.00	XGB; R^2^ 0.929	no
Soman et al.^121^	BMImBr	ANN	Concentration, T		1.50–2.91		302.5–337.9	R^2^ 0.99	no
Sahebalzamani et al.^122^	binary mixture of 0.75 IL and 0.25 water	CFNN, GRNN models	Alumina mass fraction, T	30 (80% training and 20% testing)	0.192–0.202		275.15–333.15	AAPRE 0.2910%	no
Hezave et al.^123^	21 ILs	ANN	T, P, Mw	209 data points		100–20,000 kPa	273.15–390	MARD % 0.5% MSE 1.2 × 10^–6^	no
Said et al^.,^ ^124^	MXene-IL hybrid mixture [BDMIM][BF4]	GPR, quadratic SVR		70% training, 30% testing	0.443–0.82		293–323	GPR R^2^ 0.9985 RMSE 0.004	no
Lazzús et al.^125^	41 ILs (imidazolium, ammonium, phosphonium, pyrrolidinium, and pyridinium, halides, sulfonates, tosylates, imides, borates, phosphates, acetates, and amino acids)	ANN+GA	Mw, structure of molecules, represented by the number of well-defined groups	400 data points (75%–25%)	0.10–0.22	100–20,000 kPa	273–390	AARD 0.84%, R^2^ 0.9963	no
Firoozirad et al.^126^	30 data sets (Binary mixture of one IL [C2mim][CH_3_SO_3_] + water)	ANN		80%–20%	0.192–0.222		283.15–333	training (0.9970), validation (0.9835) and testing (0.9786), Max absolute error 0.0031 W m^–1^ K^–1^, RMSE 0.006 W m^–1^ K^–1^	no
Shojaee et al.^127^	21 ILs	ANN	Melting points, Mw	209 data points training (143 data points), testing (66 data points)			293–390	AARD % 10.76%	no
Lemaoui et al.^128^	1622 data points (ILs, DESs, and ILMs, with 563, 670, and 389 data points); 40 DESs at 184 different compositions, 80 ILs, and 12 ILMs at 82 different compositions	CPs and sigma profile with ANN, DT, GBM, RF, k-NN, and MLR	σ-profiles, critical properties	training 1259, testing 363	0.05−0.61	100–65000 kPa	273–438	AARD 1.68 (σ-profiles) 2.41 (CPs) R^2^ 0.995 (σ-profiles) 0.991 (CPs)	no
Mousavi et al.^129^	50 ILs	RBF-ANN, ANN,		504 experimental data, training 404, testing 100	0.1–0.2	100–20,000 kPa	273–390	MAPRE 1.901%	no
Parashar et al.^130^	[MMIM][DMP]	MLR, ANN	T, nanoparticle concentration, and Mw	48 data points, training data (80%) and test data (20%)	0.468–0.848		298–333	ANN R^2^ 0.9946, maximum error 1.644%	no
Wan et al.^131^	44 ILs (18 cations, 21 anions)	λ-QSPR model (MLR)	σ-profiles of ions, P, T	606 experiment data points, training 500 points, testing 106 points	0.1 – 0.22	0.1 – 65 MPa	273.15–438.07	R^2^ 0.9713, RMSE 0.004304, and AARD 2.18%	no
Wang et al.^132^	644 data point (75 ILs)	KNN, SVR, RF, GBRT, and XGBoost	constitution-structure-interaction, P, T	80%–20%	0.09–0.26	100.00–101.33 kPa	273.15–390.00	R^2^ 0.9554, RMSE 0.0053, MAE 0.0025	no

One of the earliest and most frequently cited efforts in this domain is by Hezave et al.,^123^ who employed a single-hidden-layer ANN trained on 209 data points from 21 ILs, spanning temperatures from 273 to 390 K and pressures up to 20 MPa. Their model achieved a mean absolute relative deviation (MARD) of 0.5% and mean square error (MSE) of 1.2 × 10^–6^ (W m^–1^ K^–1^).^2^ However, the model inputs were limited to temperature, pressure, and molecular weight (T, P, Mw), and the study does not report evaluation on ILs outside the 21-compound set (an external test set), which makes transferability to other IL families difficult to judge. Lazzus et al.^125^ extended this approach by incorporating structural information through group counts and applied a genetic algorithm-optimized ANN. The model was trained on 400 data points across 41 ILs, including ammonium, phosphonium, and pyrrolidinium families, within the same thermophysical range. It achieved AARD below 0.9% and R^2^ 0.996 at pressures up to 20 MPa.

Studies on IL mixtures and nanofluids (reported in SI) have generally used narrow chemical domains; for example, Soman et al.^121^ modeled a single aqueous IL system (50 points, R^2^ 0.99), illustrating how high apparent accuracy can arise from interpolation within one IL family. The standout contribution in terms of scale and diversity comes from Lemaoui et al.^128^ who compiled 1,622 data points covering 80 ILs, 40 DESs, and mixtures. They evaluated six different ML algorithms and found that ANNs delivered the best performance. Using COSMO-based σ-profiles as input features, their ANN achieved an impressive R^2^ of 0.995 and showed deviations under 3% when validated against new experimental data. The trained models were subsequently used to screen over 2,200 solvent systems, effectively mapping λ trends across diverse chemical structures. This screening step demonstrated the model’s potential for guiding targeted solvent design.

Wan et al.^131^ developed a multiple linear regression QSPR model using 606 data points from 44 ILs. Their approach used COSMO-SAC charge-density surfaces and cavity volumes as descriptors. The model achieved strong metrics with R^2^ of 0.971, RMSE of 0.0043 W m^–1^ K^–1^, and AARD of 2.18%. Beyond predictive accuracy, the linear coefficients provided valuable insight into the electrostatic features most influential to λ. Cao et al.^17^ explored GC methods, pairing 20 molecular fragments with six ML algorithms on a data set of 334 points from 46 ILs (273–390 K). Their best-performing model achieved an R^2^ of 0.929. However, cross-validation revealed a critical flaw: point-based data splits overestimated accuracy by up to 5%, highlighting a common bias in earlier studies that lacked robust validation schemes. Wang et al.^132^ advanced descriptor engineering by introducing a constitution-structure-interaction composite descriptor across 644 data points from 75 ILs. Using gradient boosting regression trees, they achieved an R^2^ of 0.955 and an RMSE of 0.0053 W m^–1^ K^–1^. Their SHAP analysis revealed interaction terms as key contributions, offering chemical interpretability. However, the data set’s cation imbalance limited the model’s extrapolation capability across broader IL families.

Taken together, these studies show a clear trajectory from simple inputs (T, P, Mw) toward physics-informed descriptors (σ-profiles, COSMO-SAC surfaces) and from narrow data sets (21 ILs) toward broader compilations (80+ ILs). Reported errors for the best-performing models reach below 3% (Lemaoui et al.,^128^ Wan et al.^131^), but performance remains closely tied to data set scope. Against the evaluation checklist (Table 1), the λ literature reveals a systematic pattern: all studies in Table 2 rely on random train/test splits, none defines an applicability domain, none reports prediction uncertainty, and none releases code or trained models. Only Lemaoui et al.^128^ includes any external validation. No study reports experimental validation of ML-proposed candidates. For TES applications, this means that existing models can interpolate λ within well-represented IL families, but cannot yet be relied upon to predict this property for novel cation–anion combinations proposed during screening, a critical limitation given that λ is one of the four Tier 1 properties governing whether an IL can function as a PCM.

### 5.2 Enthalpy-Related Properties

As a Tier 1 property in the TES framework (Section 3), enthalpy of fusion (ΔH_fus_) sets the latent heat available during melting and solidification and, together with melting temperature (T_m_), determines the energy storage density of phase-change materials for TES. In ILs and related salts, ΔH_fus_ is difficult to predict from structure because it depends on how ions pack and order in the solid phase, and reported values can vary with polymorphism and phase-transition pathways. Table 3 summarizes the available ML studies for enthalpy-related properties in ILs. Notably, only one study (Saher et al.^133^) targets ΔH_fus_ directly; the remaining studies predict enthalpy of vaporization or excess enthalpy of mixing, which are thermodynamically distinct quantities that do not measure latent heat storage capacity. This extreme scarcity of ΔH_fus_-specific ML work is itself a central finding for TES.

**Table 3:** Summary of Machine Learning Studies for the Prediction of Enthalpy-Related Properties in Ionic Liquids

Reference	Enthalpy Type	Families of Ionic Liquids	ML Models	Descriptor	Size of Data set	Range of Enthalpy, kJ mol^–1^	Range of Temperature, K	Performance	GitHub Link
Saher et al.^133^	ΔH_fus_	Protic organic salts	MLREM, BRANNLP	Dragon Descriptors	Training: 69 salts Screened: 182 salts	3.4–36	345–500	*T*_m_ R^2^ 0.63 (SEE 28 °C); ΔH_fus_ R^2^ 0.82 (SEE 4 kJ mol^–1^)	no
Changzheng et al.^134^	ΔH_vap_	148 ILs	BP-ANN	σ-profile	3,150 data points (training 2,520, testing 315, validation 315)		298–631.86	Δ_vap_ *H* R^2^ 0.990, AARD 0.613%, RMSE 1.58 kJ mol^–1^	no
Kalantari et al.^135^	HE (mixing)	9 binary mixtures	PCA–BPN model	T, mole fractions (x1 and x2), Mw	483 data points	–0.92–0.354	288.15–413.15	R^2^ 0.9999, AAD 0.98%	no

Saher et al.^133^ compiled data for 69 protic organic salts from the literature together with salts synthesized in-house and modeled T_m_ and ΔH_fus_ ^133^. They used molecular descriptors encoding features of both the cation and anion and evaluated a range of linear and nonlinear models. The trained models were also applied to screen 182 additional candidate salts. For ΔH_fus_, the best linear descriptor-based models achieved an R^2^ of 0.82 with a standard error of about 4 kJ mol^–1^. For T_m_, reported performance was lower with an R^2^ of 0.63 and an error of about 28 °C. Changzheng et al.^134^ used 3,150 data points spanning 148 ILs to predict ΔH_vap_. They employed σ-profile descriptors derived from COSMO-RS, which capture the molecular surface charge distribution, and trained a backpropagation ANN (BP-ANN). The resulting model achieved an R^2^ of 0.990 and RMSE of 1.6 kJ mol^–1^, demonstrating that physics-informed descriptors can efficiently compensate for the limited availability of experimental ΔH_vap_ data. Kalantari et al.^135^ focused on excess enthalpy in nine binary IL mixtures, using 483 data points. Their model, a principal component analysis-enhanced backpropagation NN, relied solely on temperature, mole fractions, and molecular weight as inputs. Despite the simplicity of the descriptors, the model achieved near perfect fit (R^2^ 0.9999, AAD 0.98%), suggesting that, for mixing-related enthalpies, basic compositional descriptors may be sufficient. However, the model’s generalizability beyond the studied systems remains untested.

Against the evaluation checklist (Table 1), the enthalpy literature presents the weakest profile of any property reviewed: all three studies use random splits, none reports prediction uncertainty or defines an applicability domain, and none releases code, trained models, or data sets. The sole ΔH_fus_ study (Saher et al.^133^) covers only 69 protic organic salts with an R^2^ of 0.82, substantially lower than the accuracies reported for density or viscosity models. For TES screening, this means that ML-based prediction of latent heat storage capacity is effectively unavailable: no model exists that can reliably estimate ΔH_fus_ for a novel cation–anion combination, making this the most critical ML gap in the TES property pipeline.

### 5.3 Heat Capacity

As a Tier 1 property in the TES framework (Section 3), heat capacity (c_p_) is needed for sensible heat storage calculations and thermal management, and it has therefore been a recurring target in ML studies on ILs and IL-containing systems. Studies are summarized in the Supporting Information (Heat Capacity Table S1). Experimental c_p_ data sets remain uneven across IL families and are often reported over narrow composition and temperature windows, which has encouraged the use of data-driven models for interpolation within the available measurement space. Early c_p_ models often used ANNs with a small set of input variables. For example, Lashkarbolooki et al.^136^ predicted the c_p_ of binary IL mixtures using an ANN with temperature, molecular weight, T_m_, and IL mole fraction as inputs, achieving an R^2^ of 0.9975.^136^ Similarly, Valderrama and colleagues^137^ used ANNs alongside a mass connectivity index descriptor, demonstrating satisfactory accuracy for engineering applications, albeit on relatively small data sets (e.g., 477 data points across 31 ILs) with limited chemical diversity. Barati-Harooni et al.^138^ compiled 2,940 data points from 56 ILs. Their radial basis function (RBF) NN, trained on structural parameters and temperature, achieved AARD of just 0.83%. The wide temperature range covered (188–663 K) further demonstrates the reliability of their approach.

Quantum-chemistry and COSMO-based descriptors have been used to improve c_p_ prediction by encoding charge distribution features. The integration of quantum chemical descriptors, particularly those capturing anion–cation interactions, has further improved predictions. Zhao et al.^139^ and Kang et al.^140^ applied extreme learning machines (ELM) with sigma profile descriptors and electrostatic-potential-based descriptors, reporting AARD values of 0.60% and 0.44%, respectively, and R^2^ values close to 0.999. However, the ELM algorithm’s sensitivity to random initializations introduces variability in model performance and neither study reported performance stability or uncertainty quantification. Moreover, both models were trained on 46 ILs of four distinct IL families, and lacked leave-one-ion-out validation, limiting confidence in their generalizability to novel IL families.

In contrast, Esmaeili et al.^141^ trained multiple ML models on a much larger data set comprising 13,893 data points across 322 ILs. The authors identified XGBoost as the top performer, achieving RMSE of 11.389 J mol^–1^ K^–1^, R^2^ of 0.997, and AARD of 1.212%. Their validation strategy included both 10-fold and leave-one-IL-out (LOILO) testing, offering a more rigorous assessment of chemical transferability. Their deployment of a public web interface also stands out as a rare gesture toward transparency and accessibility. Similarly, Wang et al.^132^ applied gradient boosting to a data set of 11,616 data points for 263 ILs, using a composite constitution–structure–interaction descriptor. Their model achieved competitive accuracy, with an RMSE of 27.47 J mol^–1^ K^–1^.

In mixture-focused studies, Liu et al.^142^ and Fu et al.^143^ compiled 2,504 and 2,131 c_p_ data points, respectively, for binary mixtures of ILs with organic solvents and water. Both studies concluded that ANN models outperform gradient boosting models. Their use of SHAP analysis is commendable, as it revealed the strong influence of IL mole fraction and cation substituents on c_p_. However, neither study attempted cross-family validation, and the narrow temperature range (283–353 K) limits the models to local interpolation rather than general prediction.

Several other studies focus on single ILs or highly specialized systems, limiting broader applicability. Mazari et al.^144^ developed GPR and SVM models exclusively for [Bmim]­[PF6] reporting R^2^ of 0.988. While the reported accuracy is high, the analysis is restricted to a single IL, and transferability to other cation–anion combinations is not evaluated.

Work on nanoparticle-enhanced IL systems shows similar limitations. Firoozirad et al.^126^ and Sahebalzamani et al.^122^ trained NNs on 48 data points for a single IL water base fluid and reported R^2^ values above 0.99 but provided no evidence of transferability or even uncertainty quantification.

Against the evaluation checklist (Table 1), c_p_ is the most methodologically advanced of the four Tier 1 properties. Esmaeili et al. applied LOILO validation across 322 ILs and deployed a public prediction interface, the only Tier 1 study to meet both of these criteria. Data set scale ranges from 48 points for nanofluid systems to over 13,000 points for neat ILs, and the largest compilation (Esmaeili et al.) demonstrates that ensemble methods trained on diverse IL families can achieve AARDs near 1%, while Wang et al.^132^ reach comparable accuracy (RMSE 27.47 J mol^–1^ K^–1^) on a similarly large multifamily data set. However, most studies still rely on random splits, and among the large multi-IL models none reports prediction uncertainty; the only exception in the section is the single-IL GPR model of Mazari et al., whose GP formulation yields predictive variance but is confined to one cation–anion pair. Applicability domains are likewise rarely defined. For TES screening, c_p_ models are closer to practical readiness than those for λ or, but their reliability for novel cation–anion combinations beyond the training chemistry remains demonstrated by only one study.

### 5.4 Density

Density (ρ) is a Tier 2 property in the TES framework (Section 3): it determines volumetric energy density and pumping requirements in TES systems, and is the most extensively studied IL property by ML methods. Studies are summarized in Supporting Information Table S2. Early work by Lazzús et al.^145^ represented molecular structure using functional groups and combined this representation with a GC method in an ANN framework. The model was trained on 2,410 ρ data points from 250 ILs and evaluated on an additional set of 72 ILs not used during training, achieving R^2^ 0.9972. This study shows that ANN-GC models can accurately interpolate ρ across fragment combinations represented in the data set; however, the reliance on predefined groups implies that transfer to ILs containing new or sparsely represented fragments is not explicitly assessed.

Subsequent studies focused on whether comparable performance could be achieved with simpler inputs. Fatehi et al.^95^ trained a three-layer feed-forward NN using basic structural information along with temperature, pressure, and molecular weight. Their results show that reduced descriptor complexity can still yield strong predictive accuracy, within the limits imposed by the underlying data set.

Other work focused on narrowly defined systems. Najafi-Marghmaleki et al.^146^ investigated IL-water mixtures and compared radial basis function (RBF) networks with multilayer perceptron (MLP) networks. Their findings showed that the MLP design outperformed RBF for their same data set. It shows the significance of model selection even within the same class of algorithms.^146^

To improve both chemical coverage and interpretability, hybrid approaches that combine ML with GC theory have gained traction. Paduszynski et al.^147^ developed a Support Vector Regression (SVR) model regularized by group fragments, trained on 41,250 ρ records for 2,267 ILs. Their model achieved an AARE of 0.90% over a wide range of conditions (0.1–300 MPa and 220–473 K).

Rostami et al.^148^ combined least-squares SVM with 47 substructure indicators. Their model maintained high accuracy (R^2^ 0.9925) on a hold-out set of 229 points and applied genetic algorithms to tune the SVM hyperparameters, which improved predictive accuracy on the hold-out data set. In contrast, Soman et al.^149^ worked with just 48 data points focused on aqueous solutions of 1-butyl-3-methylimidazolium bromide ([Bmim]­[Br]). While their ANN model achieved high accuracy within this narrow domain (AAE 0.097%), its applicability was limited to a constrained concentration window and a temperature range of (303.2–338.2 K). Generalization to other ILs or broader conditions is not evaluated. Similarly, Mazari et al.,^144^ trained models exclusively on [Bmim]­[PF_6_] and reported R^2^ values above 0.99. Although effective for the targeted system, the study does not assess transferability beyond this single IL, limiting its relevance for broader IL screening.

Model interpretability has improved through the adoption of explainable AI techniques. For example, Chen et al.^150^ and Shu et al.^151^ applied SHAP to binary and ternary mixtures, identifying alkyl chain length, anion identity, and mixture composition as dominant factors influencing ρ.

RBF-NNs and ANN-based GC methods^152^ have outperformed simpler approaches for ρ prediction. This advantage is strongly conditioned on data set scope and validation practice. Algorithm choice also affects generalizability. While ANNs predominate in the literature (e.g., Huang et al.;^153^ Golzar et al.^154^), more recent work has explored GNNs and probabilistic models. For example, Baran et al.^97^ applied GNNs to predict IL properties directly from molecular graphs and reported R^2^ above 0.99. However, they also observed that data contaminants, such as mislabeled structures, can degrade performance, underscoring the importance of data quality in graph-based models.

Marques et al.^155^ modeled CO_2_–IL mixtures with multilayer perceptrons trained on 1,139 data points, combining critical thermodynamic properties and cheminformatic structural descriptors. Their model achieved R^2^ 0.986 and showed that combining thermodynamic constants with cheminformatic fingerprints reduces overfitting compared to using critical-property inputs. However, evaluation remains limited to a restricted set of IL families and temperature ranges. Code and trained models are rarely shared in the ρ literature, with only a small minority of studies providing reusable implementations, which limits reproducibility.

Against the evaluation checklist (Table 1), ρ has the largest IL-ML data sets of any property. Paduszynski et al.^147^ compiled 41,250 records across 2,267 ILs, two to three times larger than any other single-property compilation. Two other practices stand out because they are uncommon here: Lazzús et al.^145^ evaluated on 72 ILs held out from training, and Mazari et al. reported GPR-based prediction uncertainty. Baran et al.^97^ showed that GNNs can predict ρ directly from molecular graphs at R^2^ above 0.99, but their results degraded on mislabeled structures, which is a useful reminder that data quality matters as much as model choice. Beyond these, the pattern is familiar: random point-wise splits, few explicit applicability domains, and code or trained models that are almost never released. Density is nonetheless further along than most TES properties. What is still missing is any test of transfer across cation–anion families under TES-relevant conditions, and single-IL studies such as Soman et al.^149^ and Mazari et al.^144^ remain common.

### 5.5 Viscosity

Viscosity (η) is a Tier 2 property in the TES framework (Section 3): together with ρ, it determines the pumping penalty for circulating an IL through a TES system and is one of the most extensively modeled IL properties. Studies on ML-based η prediction for ILs are summarized in Supporting Information Table S3. The table makes clear that reported performance is strongly shaped by data set scope, descriptor choice, and how validation is performed, so headline metrics are not directly comparable across studies.

The majority of the literature focuses on ANNs.^156,157^ For example, studies using MLPs have achieved high accuracy using only minimal input features, such as temperature and anion polarizability, with a reported R^2^ of 0.999 for ternary mixture η prediction^157^ and an MPE as low as 2.33% for choline carboxylate ILs.^158^ Dutt et al.^159^ trained an ANN that fit the training data reasonably well (6.6% deviation), showed weaker performance under external validation relative to an empirical correlation, indicating that model quality is sensitive to data set composition and evaluation protocol.

Work that expands chemical coverage tends to trade lower apparent accuracy for more informative robustness testing. Paduszynski et al.^160^ trained a feedforward ANN using 242 GC-type chemical descriptors using a large chemically varied data set (over 13,000 points; 1,484 ILs) and reports a higher test deviation (∼14.7%), but the result is more meaningful because it is obtained over a broader chemical space. Ma et al.^153^ likewise support the value^161^ of GC-based structural encodings in mixtures, but the consistently high R^2^ values reported for ternary systems should still be read in the context of the mixture families represented in the training data.

In addition to ANNs, SVR, random forest (RF), and gradient boosting are widely used for η prediction. Many papers report improvements over MLR, but the apparent gains often depend more on data set scope and the split strategy than on the specific algorithm. Zhao et al.^162^ reported an SVM with AARD 3.95% for imidazolium ILs, outperforming MLR. Huwaimel et al.^163^ reported very high R^2^ values for RF and gradient-boosting models on IL-water mixtures, but the validation relies on random splits within the sampled ion set, so performance for unseen cation–anion combinations is not established. Algorithms like XGBoost and LightGBM are often used in conjunction with GC techniques, have shown competitive performance in modeling complicated mixtures, including IL-water-organic solvents mixtures.^151,164^

Ding et al.^165^ compared fingerprints, molecular descriptors, and combined representations across a data set assembled from 281 cations and 114 anions, and found that combined representations performed best. More importantly, Wang et al.^132^ showed that split design can inflate metrics when closely related structures appear in both training and test sets, which can overstate reliability for new IL chemistries.^132^ Many η studies are focused and examine only one or a few IL families.^156,166^ By contrast, a few large compilations span hundreds to thousands of chemically distinct ILs.^132,160,167^

Physics-informed workflows aim to correct systematic biases rather than fit from scratch. Fan et al.^168^ trained CatBoost models on residuals relative to COSMO-RS using quantum-chemical descriptors (R^2^ 0.9999), though an R^2^ this close to unity on a limited data set warrants caution about overfitting to the training chemistry. Mohan et al.^169^ combined COSMO-RS sigma profile with SHAP analysis (R^2^ 0.984). Lemaoui et al.^167^ extended this direction to large-scale screening (R^2^ 0.907) and released an inverse-design tool. Across these studies, the main limitation is whether training data and validation support use outside the original chemical space and conditions.

Against the evaluation checklist (Table 1), η shares ρ’s advantage in scale. Paduszynski et al.^160^ assembled over 13,000 points across 1,484 Ils, but shares its validation weaknesses too. Wang et al.^132^ showed explicitly that split design inflates reported accuracy when structurally related ILs land in both training and test sets, yet most later studies still use random splits without addressing it. Dutt et al.^159^ found that testing against an empirical correlation exposed weaker performance than internal metrics implied. Lemaoui et al. released an inverse-design tool, one of the few instances of shared code in this literature. The recurring gaps are the same as elsewhere: explicit applicability domains are essentially absent, none of the multi-IL models reports prediction uncertainty, and no proposed candidate has been synthesized and measured. This matters for TES because η sets the pumping cost and heat-transfer efficiency of a working fluid. No model has yet been validated on cation–anion combinations outside its training families, which leaves this Tier 2 property without a reliable screening tool for new IL candidates.

### 5.6 CO_2_ Solubility (Methodological Case Study)

CO_2_ solubility is not a primary TES property; it is relevant mainly to carbon capture and separation. It is included here as a methodological case study because this subfield offers the largest IL-ML data sets, the most diverse model comparisons, and the most rigorous validation practices available, lessons that transfer directly to the less-developed TES-property models reviewed above. Studies are summarized in Supporting Information Table S4. ANNs remain common in this space. For example, Agwu et al.^170^ reported an ANN with R^2^ 0.992 (RMSE 0.04) using temperature, pressure, and bulk IL properties over 0.0098–72.24 MPa and 292–450 K. Similarly, Mesbah et al.^171^ reported R^2^ 0.9987 for 20 ILs over 0.25–100.12 MPa and 278.15–450.49 K. These studies show that ANN models can fit broad pressure and temperature ranges, but their reliability for new IL chemistries depends on how well the training set spans cation–anion families and descriptor space.

Ensemble methods are also widely reported when richer descriptors are available. Laakso et al.^96^ used σ-profile descriptors in a RF model for 124 ILs and achieved R^2^ 0.9754 (MAE 0.0257) across 243.15–453.15 K and 0.798–49,990 kPa. Rozhenko et al.^172^ applied XGBoost with 26 inputs for 43 imidazolium ILs and reported R^2^ of 0.999 and an RMSE of 0.0077 across pressures from 0 to 95 bar and temperatures from 298 to 353 K. While these results support the usefulness of tree-based models for solubility prediction within the sampled families, several high-accuracy studies remain concentrated in a small number of IL classes, which limits what can be concluded about transfer to underrepresented chemistries.

Descriptor choice is a recurring determinant of model behavior. Early studies often relied on fundamental thermodynamic properties like critical temperature, critical pressure, and molecular weight.^173,174^ Whereas later work increasingly incorporates structural and physics-derived representations. Valeh-E-Sheyda et al.^175^ reported R^2^ 0.995 for pyridinium ILs using chemoinformatics descriptors. Liu et al.^176^ showed that explicit molecular structure encodings improve predictive performance when coupled with ML, relative to more generic descriptor sets. Descriptors derived from techniques, such as GC methods, and COSMO-RS^18,177,178^ have also shown high potential by effectively integrating data-driven approaches with physical principles.

Data set size and chemical diversity remain limiting factors for model reliability. Studies based on larger data sets, such as Bastami et al.^179^ (16,480 data points across 296 ILs) provide a stronger basis for model evaluation, but many papers still focus on specific IL families. Aghaie et al.^174^ compared LSSVM, decision trees, RF, and MLR using two input strategies and found that tree-based models trained on structural descriptors were more stable than models relying only on thermodynamic variables, consistent with solubility being controlled by local chemical environments rather than bulk constants alone.

Qin et al.^18^ showed that COSMO-RS can give large errors for gas solubility, particularly for N_2_, and used XGBoost with molecular descriptors to correct COSMO-RS predictions, reducing CO_2_ errors to below 1% in their evaluation set and improving N_2_ predictions despite the smaller data set. This approach is attractive for extending COSMO-RS in data-limited regimes, but the corrected model remains bounded by the coverage of the training data and the descriptor space used for the residual mapping.

Generative design has also been applied. Chen et al.^181^ combined a variational autoencoder with an ANN predictor and particle swarm optimization to generate new IL candidates with improved CO_2_ solubility (reported MAE 0.022) and screened more than 5,000 structures against 3,735 known ILs under standard conditions. The workflow expands candidate generation, but the top predictions remain computational and are not experimentally validated in the study.

CO_2_ solubility is the most methodologically mature IL-ML subfield, and it still misses several checklist criteria (Table 1). Most studies use random splits, none defines an explicit applicability domain, and prediction uncertainty is rarely reported. Chen et al.^181^ generative-design candidates are a case in point: proposed computationally, never synthesized. If even the strongest subfield looks like this, the rest of the field has further to go.

Three lessons carry over to TES properties. First, large data sets are achievable, with Bastami et al.^179^ reaching 16,480 points across 296 ILs, and they buy real reliability compared with the few-hundred-point sets typical of λ or ΔH_fus_. Second, physics-informed descriptors like σ-profiles earn their cost when the target property depends on specific intermolecular interactions. Third, correcting a physics-based baseline such as COSMO-RS with a learned residual is a practical fallback when experimental data is thin, which is the norm for most TES-relevant properties today.

### 5.7 Melting Point

Melting point (T_m_) is a Tier 1 property in the TES framework (Section 3): it defines the phase-transition temperature and must fall within the operating window of the target application, making it the primary screening criterion for IL-based phase-change materials. Studies on ML-based melting point prediction for ILs are summarized in Supporting Information Table S5. Early efforts to predict T_m_s of ILs using ML date back to 2008, when Bini et al.^182^ evaluated 126 pyridinium bromides using an early RNN model. The model reported R^2^ 0.8725 and MAE 19.37 K. The data set is confined to a single cation family, so the reported accuracy largely reflects interpolation within a narrow chemical domain. Varnek et al.^183^ expanded the scope to 717 bromide salts from pyridinium, imidazolium, and quaternary ammonium families and compared k-NN, SVM, and associative NNs. Reported RMSE varied across IL subsets ranging from 26.2 to 49.3 °C indicate that performance remains moderate even when multiple cation families are included, and that results depend strongly on how the data set is partitioned.

More recent work has used larger data sets and richer representations. Feng et al.^184^ trained a GNN on 3,080 ILs and reported RMSE 37.06 K and MAE 28.79 K. Their analysis also highlights a data set imbalance issue (71% of ILs below 400 K), which can bias models toward midrange predictions unless addressed explicitly. Subsequent work by Paduszyński et al.^185^ reported lower errors (RMSE 19 K) using ensemble learning with merged descriptor families, but the resulting models are less transparent in terms of mapping descriptors to chemical mechanisms. Liang et al.^186^ introduced a pseudo-Siamese convolutional NN using filtered Mordred descriptors and achieved MAE 24.36 °C and RMSE 31.56 °C; the improvement over earlier descriptor-based baselines come with increased representation and model complexity, which also raises the computational burden of training and descriptor generation.

A widely used benchmark is the study by Venkatraman et al.^187^ who compiled a data set of 2,212 ILs (1,369 cations; 141 anions) from approximately 300 literature sources and evaluated multiple ML algorithms, including ensemble tree-based models, SVM, and k-NN. Ensemble models gave the best performance in their benchmark, and the study also framed T_m_ prediction as a classification problem (below vs above 100 °C), which is often more aligned with practical screening than minimizing regression error alone. Their feature analysis further suggests that quantum-chemical descriptors can contribute useful signal, but model reliability remains conditioned on the representativeness of the underlying training chemistry.

T_m_ stands out on the checklist for one good reason and several bad ones. The data sets are among the largest for any Tier 1 property, up to 2,212 ILs in Venkatraman et al.,^187^ and Venkatraman’s classification framing, below versus above 100 °C, is a practical screening idea rarely seen elsewhere. The rest is familiar. Splits are mostly random, though Varnek et al.^183^ used external cross-validation; uncertainty is seldom reported, though Venkatraman gives per-compound estimates; applicability domains are absent; and trained models stay unpublished, even where Feng et al.^184^ shared the underlying data. What matters most for TES is accuracy. T_m_ is harder to predict than ρ or c_p_, and the best models still report RMSE of 19 to 37 K. An error that size can move a candidate IL right out of its target window for latent heat storage, which makes T_m_ accuracy one of the real bottlenecks in AI-guided screening for PCMs.

### 5.8 Toxicity

As a Tier 3 property in the TES framework (Section 3), toxicity is the only safety/sustainability constraint with dedicated ML coverage for ILs. Studies are summarized in Supporting Information Table S6. For ILs to be deployed in energy storage, toxicity constraints need to be treated as design targets rather than postscreening filters. Most ML studies focus on cytotoxicity in the IPC-81 leukemia rat cell line.

Early IPC-81 models combined relatively simple structural encodings with linear or shallow nonlinear learners. Fatemi et al.^188^ compared MLR with MLP models using feature selection and reported high predictive performance on IPC-81 data. Torrecilla et al.^189^ introduced sigma profile descriptors in a COSMO-RS-based NN and linked cytotoxicity trends to electronic and steric features. These studies established that toxicity can be modeled from structural and physics-derived descriptors, but their usefulness for screening is conditioned on chemical coverage and whether validation separates closely related ions.

Abdellatif et al.^190^ trained an SVM on IPC-81 data (304 ILs) and used an external validation set (14 ILs), reporting R^2^ 0.9742 with descriptor-based inputs and explicit test-set evaluation. Wang et al.^191^ follow the same direction by using subgroup counts instead of quantum descriptors and benchmarking NN versus SVM under cross-validation, which is a reasonable choice when descriptor calculation is the bottleneck, but it also limits mechanistic interpretation to whatever the subgroup dictionary encodes.

Fan et al.^192^ modeled *Vibrio fischeri* toxicity using RF and XGBoost, optimized hyperparameters with Bayesian optimization, and used SHAP to interpret feature effects; the work also illustrates how model conclusions are end point-specific even when the same ML workflow is used. Tabaaza et al.^193^ reported a lower R^2^ of 0.79 using the same algorithm, underscoring the influence of data set characteristics on model performance.

Zhang et al.^194^ applied SHAP to an RF toxicity model and found that oxygen-containing functional groups tend to reduce toxicity, whereas substructures surrounding cationic carbon atoms exert the strongest influence. Similarly, Wu et al.^195^ developed RF and XGBoost QSAR models for AChE inhibition and used a reduced descriptor set to maintain performance while extracting feature importance; their analysis again points to cation-dominated contributions and hydrophobicity-related effects within that end point.

Model transferability remains highly dependent on data set diversity and size. While internal metrics may appear satisfactory, models trained on small data sets such as the 56 ILs used by Grzonkowska et al.^196^ to predict *Vibrio fischeri* toxicity (R^2^ 0.78) often fail external validation. In contrast, larger data sets, such as those employed by Wu et al.^195^ and Abdellatif et al.^190^ (304 ILs), yield more robust models. Zhao et al.,^197^ compiled a data set of approximately 4,000 toxicity data points, although modeling was performed on a subset of 100 ILs. Broader logEC_50_ ranges, such as −0.24 to 4.90 reported by Wu et al.,^198^ improve generalization across wider chemical spaces compared to narrower distributions.

Recent advances demonstrate that integrating deep learning-based molecular embeddings such as those from ChemBERTa,^199^ with conventional ML algorithms significantly improves predictive accuracy. Although most toxicity models rely on standardized biological assays like logEC_50_ some studies,^189,200–204^ incorporate quantum-chemical descriptors such as σ-profiles, providing a more fundamental link between molecular electronic structure and observed toxic effects.

Across Table S6, two limitations recur. First, many models remain assay-bound, trained and validated inside a narrow end point and chemical domain, so high R^2^ values often reflect interpolation among closely related ions rather than predictive reliability for new IL families. Second, interpretability claims depend on descriptor choice: subgroup counts support fast screening, while sigma-profile and related descriptors support more chemically grounded attribution, at higher computational cost. A practical path forward is to report end point-specific models with explicit external validation, and to state the applicability domain in terms of cation and anion families, not only aggregate error metrics.

Toxicity models are more methodologically varied than most sections here. Abdellatif et al.^190^ used an external validation set, and SHAP shows up across several studies. The gaps are still real. No study defines an applicability domain by cation and anion family, none releases code or trained models, and no ML-screened low-toxicity IL has been made and measured. There is also a scope problem for TES. Toxicity work leans heavily on cytotoxicity assays, IPC-81 above all, so even an accurate model captures just one slice of the risk. The other Tier 3 constraints, corrosion, thermal decomposition, and environmental fate, sit outside the toxicity models reviewed here.

### 5.9 Surface Tension

Surface tension (γ) is a Tier 2 property in the TES framework (Section 3): it governs interfacial behavior and wetting in heat-exchange surfaces, influencing heat-transfer efficiency in TES systems. Studies on ML-based γ prediction for ILs are summarized in Supporting Information Table S7. Early ML studies showed that γ can be fit accurately, but many results were obtained in narrow chemical domains. Golzar et al.^205^ used ANNs and genetic function approximation for quaternary ammonium ILs and reported R^2^ 0.9962, but the model was developed on only five ILs, so the performance mainly reflects interpolation within a very limited chemistry set. Lazzús et al.^206^ targeted low surface-tension regimes (15–62 mN m^–1^) and coupled GC features with an ANN optimized by a gravitational search algorithm (MAPE 1.29% across 162 ILs). This scale is more useful for screening, but the focus on low γ and the choice of optimization strategy make it difficult to compare directly with models trained on broader ranges. Paduszyński et al.^207^ addressed this limitation by compiling 6,114 measurements for 570 ILs and developing a GC framework spanning 15.5–100 mN m^–1^, including high γ systems that are underrepresented in most earlier data sets. The main value of this work is coverage rather than a single headline metric, because it exposes how strongly reported performance depends on whether high γ regions and less common families are included.

Atashrouz et al.^208^ introduced an LSSVM model optimized with genetic algorithms using 573 points from 32 binary IL mixtures. The reported accuracy (R^2^ 0.987; RMSE 1.629 × 10^–3^) is strong for this data set size, but the chemical space remains limited to the studied mixtures and the model inputs mix composition, bulk properties, and descriptors, which complicates transfer outside the training domain. Soleimani et al.^209^ used much larger data sets (4,000 points) and broader mixture coverage (48 ILs with 20 nonionic compounds over 278.15–348.15 K), and reported very low errors. These results support the use of ensemble trees for mixture data sets, but the central question remains whether the data split and mixture diversity support prediction for new IL-solvent combinations rather than new points from the same systems.

Recent γ work has converged on ensemble methods (boosting, bagging) applied to conventional base learners, which stabilize predictions across heterogeneous feature sets while preserving some interpretability. Obaid et al. reported that AdaBoost ensembles around GPR, SVR, and decision trees, combined with GA feature selection, improve performance on midsized IL data sets. Alshahrani et al. and Abourehab et al. reported similarly strong results using bagging with kNN and heuristic feature searches. Across these studies, ensembling often improves fit, but the practical contribution depends on whether the underlying data sets span multiple IL families and whether the validation avoids reusing closely related mixtures in both training and test sets.^208^

Physics-derived descriptors have also been used to improve feature quality. Mohan et al.^210^ combined COSMO-RS descriptors with gradient boosting and reported R^2^ 0.994 (RMSE 1.71 mN m^–1^) over 2,524 points spanning 263–533 K. This approach is valuable when the aim is to encode solvation physics explicitly, but the need to generate COSMO-RS profiles adds computational cost that can limit throughput relative to purely structural or GC representations.

More recent models explore higher-capacity learners and alternative representations. Bawazeer et al.^211^ optimized tree-based models on 1,042 points from 69 ILs (extra trees: R^2^ 0.979; MAPE 2.05%), but the data set scale remains modest relative to the diversity of IL chemistry, so transfer to new families is not established by accuracy alone. Zhu et al.^212^ combined SMILES-based representations with GC features and reported strong performance on 2,168 points from 289 ILs (R^2^ 0.995; RMSE 0.686 mN m^–1^). The practical interest here is the attempt to retain interpretability while increasing coverage, but the extent to which the learned relationship extrapolates beyond the represented chemistries still depends on the descriptor space and validation design.

γ is where the field has clearly settled on ensemble methods. Boosting and bagging models deliver strong accuracy across the studies here. Paduszyński et al.^207^ offers the broadest coverage at 6,114 points and 570 ILs, and Mohan et al.^210^ shows what COSMO-RS descriptors add by encoding solvation physics directly. The gaps, though, are the ones seen everywhere else. Splits are mostly random, explicit applicability domains are absent, uncertainty goes unreported, and trained models stay unpublished. γ is a secondary target for TES, well behind the Tier 1 properties, but it still has to be predicted accurately to engineer heat-exchange interfaces and judge wetting behavior in a working system.

## 6 Conclusions and Future Perspective

This review synthesized and summarized the current state of the art in the usage of AI and ML to predict IL physicochemical properties relevant for TES applications. This work shows that ML models can be practically useful for screening, but mainly within the IL families and temperature–pressure ranges represented in their training data sets. Unfortunately, many papers report very high model accuracy, yet these results often come from narrow chemical domains or closely related mixtures, so performance is best interpreted as interpolation rather than reliable prediction for new cation–anion combinations. Furthermore, data set coverage is uneven across properties and families, and validation is often not designed to test transfer to unseen ions, so out-of-domain reliability remains uncertain. In addition, most studies do not release code, trained models, or processed data sets, which limits reuse and makes it difficult to benchmark progress across properties. As a result, most published models can narrow candidates inside well-studied chemistries, but they provide weaker support for proposing genuinely new IL structures that meet performance targets.

The three-tier framework introduced in Section 3 exposes a structural mismatch between ML coverage and TES relevance. Tier 2 properties (density, viscosity) have the largest data sets, with compilations exceeding 40,000 data points and models approaching 1% error within known IL families; yet these process properties are not the primary determinants of energy storage capacity. The most TES-critical Tier 1 properties remain the least explored: enthalpy of fusion has one ML study covering 69 salts (R^2^ 0.82), thermal conductivity has fewer than ten studies with no transfer validation, and melting-point prediction errors of 19 to 37 K are large enough to shift candidates out of their target operating window. Heat capacity is the most methodologically advanced Tier 1 property, with one study (Esmaeili et al.^141^) applying leave-one-IL-out validation. Tier 3 coverage is limited to a single toxicity assay; corrosion, thermal decomposition, and cost remain entirely unaddressed by ML. Across all nine properties and the evaluation checklist (Table 1), no study defines an applicability domain, prediction uncertainty is almost never reported, code and trained models are rarely released, and experimental validation of ML-proposed candidates is absent.

To make ML usable for discovery, the field needs to shift from isolated, single-property fits toward screening under coupled constraints that reflect real selection problems under the relevant operating conditions. Progress will depend on assembling data sets with explicit coverage by cation and anion families and operating ranges, adopting transfer-focused validation that holds out ions or families rather than random splits, reporting uncertainty or applicability limits when models are used for selection, and releasing data sets and models so results can be reproduced and compared. Under these conditions, ML can move from providing accurate fits within known IL families to supporting defensible identification of new candidates for experimental testing and application.

## Supplementary Material



## Notes

Detailed Web of Science search methodology and search strings; property-specific literature summary tables for heat capacity, density, viscosity, CO_2_ solubility, melting point, toxicity, and surface tension (PDF)
