# Supplementary material for: Artificial Intelligence in the Discovery and Design of Ionic Liquids for Thermal Energy Applications
Source: J Chem Inf Model. 2026 Aug 28;66(19):12231–53. doi: 10.1021/acs.jcim.6c02407 (PMC13663856; doi:10.1021/acs.jcim.6c02407)
Supplement: Supplementary file 1 [file ci6c02407_si_001.pdf]

Supporting Information

Artificial Intelligence in the Discovery and Design of Ionic Liquids for Thermal Energy Applications

Pawan Chhipa[1], Zachariah S. Baird[1], Dinis O. Abranches[2*], Oliver Järvik[1*]

[1]Department of Energy Technology, Tallinn University of Technology, Ehitajate tee 5, 19086, Tallinn, Estonia
[2]CICECO-Aveiro Institute of Materials, Department of Chemistry, University of Aveiro, 3810-193 Aveiro, Portugal
* Corresponding authors: jdinis@ua.pt, oliver.jarvik@taltech.ee

Table of Contents

Literature Search Methodology Using Web of Science S3

Literature-Summary Tables S4

Table S1. Literature-Summary of Machine Learning Studies for the Prediction of Heat Capacity in Ionic Liquids S4

Table S2. Literature-Summary of Machine Learning Studies for the Prediction of Density in Ionic Liquids S8

Table S3. Literature-Summary of Machine Learning Studies for the Prediction of Viscosity in Ionic Liquids S16

Table S4. Literature-Summary of Machine Learning Studies for the Prediction of CO_2 Solubility in Ionic Liquids S25

Table S5. Literature-Summary of Machine Learning Studies for the Prediction of Melting Point in Ionic Liquids S31

Table S6. Literature-Summary of Machine Learning Studies for the Prediction of Toxicity in Ionic Liquids S34

Table S7. Literature-Summary of Machine Learning Studies for the Prediction of Surface Tension in Ionic Liquids S39

References S44

Literature Search Methodology Using Web of Science

For Figure 4, publications were retrieved from the Web of Science Core Collection using abstract-level searches:
“Ionic liquid*” AND “<Models>” AND “<Property>”

Wildcards (*) were used where appropriate to capture term variations, such as “Ionic liquid*”, “viscosit*”, and “conductiv*”. Retrieved records were manually screened to confirm that the selected property was modeled or analyzed using machine learning in ionic liquids.

The <Models> query was:
("Machine Learning" OR "Artificial Intelligence" OR "Neural Network*" OR "Gaussian Process*" OR "Linear Regression" OR "XGBoost" OR "Decision Tree*" OR "Multiple Linear Regression" OR "Random Forest*" OR "SVM*")

For Figure 5, individual ML models were searched separately using abstract-level queries of the general form “Ionic liquid*” AND “<Model Name>”, with wildcards applied where appropriate. No additional manual screening was performed for these searches. Therefore, the reported values represent raw abstract-level matches. A publication may contribute to more than one model category when multiple ML algorithms are mentioned in its abstract.
All reported searches reflect records available up to 31 December 2025.

Literature-Summary Tables

Table S1. Literature-Summary of Machine Learning Studies for the Prediction of Heat Capacity in Ionic Liquids

Reference	Families of ILs	ML Models	Descriptor	Size of Dataset	Heat Capacity, J mol^{-1} K^{-1}	Range of Pressure, kPa	Range of Temperature, K	Performance	GitHub Link
Barati-Harooni et al.[1]	56 ILs	RBF-NN, MLP-NN	T, Mw, chemical structure information	Total 2,940 data points (80% - 20%)	226 - 1,413		188.06 - 663.1	AARD 0.83%, R^2 0.9931	no
Sattari et al.[2]	82 ILs	genetic function approximation (GFA)		Total 3,726 data points (80% - 20%)	259.09 - 1,805.7		180.06 - 663	R^2 0.990, AARD 1.68%, RMSE 18.42 J mol^{-1} K^{-1}	no
Lashkarbolooki et al.[3]		ANN	T, Mw, T_m, X_{IL}	Total 1,571 data points			278.15 - 348.1	AARD 1.60%, R^2 0.9975	no
Daneshfar et al.[4]	4 ILs	SGB, RBF-ANN, MLP-ANN	nanoparticle concentration (x) and T, Mw, ω, Tc	Total 571 data points; training 429, testing 142			298.15 - 618.15	SGB: MSE 0.00249, R^2 0.987	no
Esmaeili et al.[5]	322 ILs	linear regression, SVM, RF, AdaBoost, CatBoost, XGBoost, KNN, MLP, CNN, RNN	36 features	Total 13,893 data points	100 - 1,805		187.36 - 524.87	XGBoost, RMSE 11.389, R^2 0.997, AARD 1.212%	Link
Kang et al.[6]	46 ILs (imidazolium, pyridinium,	MLR, ELM	electrostatic potential surface area S_{EP} quantum	Total 2,416 data points; training 1,933, testing 483	254.0 - 1,805.7		223.1 - 663	ELM model (R^2 0.999, AARD	no

	pyrrolidinium, phosphonium)		chemistry descriptors, six-parameter					0.44%), MLR model (R^2 0.988, AARD 2.29%)	
Zhao et al.[7]	46 ILs (imidazolium, pyridinium, pyrrolidinium, phosphonium)	MLR, ELM	Mw, T, four sigma profile descriptors	Total 2,416 data points; training 1,933, testing 483	254.0 - 1805.7		223.1 - 663	ELM model (R^2 0.998, AARD 0.60%), MLR model (R^2 0.985, AARD 2.72%)	no
Shu et al.[8]	8 IL-organic solvent-organic solvent	ANNs, XGBoost, and LightGBM		Total 1,057 data points; training 741, validation 159, testing 159	180.8 - 374		293.15 - 308.15	ANN model: MAE 1.7320, R^2 0.9929	no
Valderrama et al.[9]	56 imidazolium-type	analytical model directly derived from ANN	T, Reference Cp at 298.15 K, mass connectivity index (λ)	Total 1,135 data points; training 912 (41 ILs), testing 172 (11 ILs), validation 51 (4 ILs)	278 - 1,020		190 - 425	AARD 1.64%	no
Koutchoukali et al.[10]	104 ILs	multiple linear regression	mass connectivity index	Total 5,791 data points; training 3,238, testing 2,553				AARD 9.18%	no
Keller et al.[11]	17 ILs tetraalkylphosphonium cations and aprotic N-heterocyclic anions	linear regression, SVR model	T, volumic heat capacity, COSMO-RS sigma profiles	Total 37 data points; training 13 ILs, testing 4 ILs			293 - 363	AARD 3.0%	no
Liu et al.[12]	17 ILs	ANN, XGBoost, LightGBM	COSMO-RS (Sigma profile)	Total 2,504 data points; training 1,601, validation 349, testing 353		1 - 1.03	283.15 - 353.20	ANN model MAE 0.0557, R^2 0.9952	no

Fu et al.[13]	31 ILs	ANN, XGBoost, LightGBM		Total 2,131 data points; training 1,527, validation 294, testing 307				ANN-GC: R^2 0.9130, MAE: 0.0842, MSE: 0.0227	no
Valderrama et al.[14]	31 ILs	ANN		Total 477 data points; training 31 ILs					no
Sattari et al.[15]	82 ILs, 39 cations 24 anions	ANNs, SVM	3D chemical structures, ChemAxon's dff descriptors	Total 3,726 data points; training 3,001 (61 ILs), testing 725 (21 ILs)	259.09 - 1,805.7		180.06 - 663	AARD 1.70%, R^2 0.993, RMSE 15.11 J mol^{-1} K^{-1}	no
Firoozirad et al.[16]	Binary mixture of one IL [C2mim][CH3SO3] + water + different mass concentrations of alumina nanoparticles	ANN		Total 48 data points; training 34, validation 7, testing 7	1.729-2.095 J g^{-1} K^{-1}		298.15 - 333.15	R^2 0.9959, MAE 0.006963, RMSE 0.002 J g^{-1} K^{-1}	no
Wang et al.[17]	263 ILs	KNN, SVR, RF, GBRT, and XGBoost		Total 11,616 data points (80% - 20%)	93.00 - 2,040.0	100.0 - 101.3	183.45 - 663.10	R^2 0.9882, RMSE 27.4681, MAE 8.5443	no
Azadfar et al.[18]	143 ILs	ANN	T, Mw and structural-related parameters for each IL	Total 7,059 data points	256.1 - 1,810		181.25 - 524.87	AARD 1.14%	Link
Chen et al.[19]	133 ILs (IL-IL mixtures (Im, Pyr, Py, Pip, N, P))	ANN, XGBoost, and LightGBM	group contribution (GC) method	Total 730 data points; training and validation 619, testing 111			263.64 - 533.2	ANN R^2 0.9999	no
Mazari et al.[20]	[Bmim][PF6]	GPR models, SVM			400 - 440	0.1 - 100 MPa	283 - 353	R^2 0.988, AARD 0.074646%	no

Parmar et al.[21]		ANN	calibration standard amount, the sample amount, and a volume ratio	Total 800 data points; training 80%, validation 10%, testing 10%	525 - 600		293 - 343	R^2 0.9939	no
Sahebalzamani et al.[22]	IL [C2mim][CH3SO3] (binary mixture of 0.75 ionic liquid and 0.25 water)	CFNN, GRNN	mass concentration of alumina, T	Total 48 data points; training 80% and testing 20%			298.15 - 333.15	AAPRE 0.2519%	no
Huang et al.[23]	9 binary mixtures of ILs	ANN	T, X_{ILs}, properties (surface tension, heat capacity and thermal conductivity)	Total 603 data points, 9 correlation dataset, validation 594			283.15 - 353.2	AARD 0.93%	no

Table S2. Literature-Summary of Machine Learning Studies for the Prediction of Density in Ionic Liquids

Reference	Families of ILs	ML Models	Descriptor	Size of Dataset	Density	Range of Pressure, kPa	Range of Temperature, K	Performance	GitHub Link
Fatehi et al.[24]	49 ILs	FFNN	Mw, T, P, structural information	Total 1,631 data points; training 1,330, validation 301, testing 301		0.1 - 100 MPa	273.15 - 393.17	AAE 0.014%	no
Cancilla et al.[25]	[bmim][BF4], 2-propanol, and water	MLR, ANN		Total 72 data points; training 70%, verification 20%, simulation 10%			298.15	MPE 0.227%	no
Díaz-Rodrígue z et al.[26]	two IL binary mixtures	ANN		Total 156 data points; training 85%, verification 15%			273.15 - 353.15	MPEs 0.11%	no
Chen et al.[19]	133 different IL-IL binary mixtures	ANN, XGBoost, and LightGBM	GC	Total 3,626 data points; training 3,080, testing 546			263.64 - 533.2	ANN R^2 0.9998, MAE 0.00135	no
Mazari et al.[20]	[Bmim][PF6]	three GPR models, SVM			1-1.6 $g \cdot cm^{-3}$	0.1 - 173 MPa	278 - 391	R^2 0.9930, AARD 0.1354%	no
Najafi-Marghma leki et al.[27]	binary mixture of water and ionic liquids	ANN, MLP NN, RBF NN	T, water ratio, Mw, boiling temperature, Tc, Pc	Total 453	0.9748 - 1.40 $g \cdot cm^{-3}$		278.15 - 348.15	MLP-ANN, R^2 0.992	no
Huang et al.[28]	54 binary mixtures and 9 ternary mixtures with molecular solvents	extended Riedel (ER) model, ANN model		Total 1,985 data points; training 70%, cross validation 15%, testing 15%			278 - 348	ANN R^2 0.9981, AAD 0.37	no

Soman et al.[29]	BMImBr IL	ANN	Concentration, T	Total 48 data points; training 75%, testing 25%	949.8-998.5 kg·m^{-3}		303.2 - 338.2	MSE 1.27, RMSE 1.13 and AAE 0.097%	no
Huang et al.[23]	33 binary mixtures	ANN, semi-empirical model	T, $X_{IL,}$ properties (surface tension, heat capacity and thermal conductivity)	Total 1,104 data points; training 70%, validation 15%, testing 15%			298.15	AARD 1.1%, ANN AARD 0.42%	no
Sahebalzamani et al.[22]	binary mixture of 0.75 ionic liquid and 0.25 water	CFNN and GRNN	T, alumina mass fraction	Total 30 data points	1.228-1.284 g·cm^{-3}		288.15 - 333	CFNN-LM model: AAPRE 0.0088%	no
Safarov et al.[30]	[OMIM][PF6]	correlation model Vogel-Tamman-Fulcher (VTF)		Total 37 data points			278.15 - 413.15	AAD 1.1%	no
Marques et al.[31]	CO_2-IL mixtures (40 distinct IL families)	MLP-ANN	IL critical properties, structural descriptors	Total 1,139 data points; training 70%, validation 15%, testing 15%		0.003 - 247.080 bar	297.80 - 353.15	R^2 0.986	Link
Bendimerad et al.[32]	1,161 ILs	Logistic Regression, SVM, RF, and XGBoost	Mordred					Classification SVM: recall 0.99, precision 0.45, PR-AUC 0.94, F1 0.61,	Link

								MCC 0.66	
Lazzús et al.[33]	131 imidazolium-based ILs	ANN	molar mass and the structure of the molecule	Total 1,736 data points	983 - 2,151 $kg \cdot m^{-3}$	99 - 206,940	258 - 393	AARD, 0.44; R^2 0.9934	no
Lazzús et al.[34]	250 ILs	ANN	T, P, M, and the structural groups	Total 2,410 data points	0.87 - 2.4 $g \cdot cm^{-3}$	99 - 206,940	273 - 393	R^2 0.9972	no
Kianfar et al.[35]	3 pyridinium-based hydrophobic ILs	ANN			1.4025 - 1.4685 $g \cdot cm^{-3}$		278.15 - 353.15	MSE 7.5714 * 10^{-7}	no
Shojaee et al.[36]		ANN		Total 1,668 data points			174.67 - 288.29	AARD 1.56%	no
Yousefi et al.[37]		MLP ANN		Total 568 data points; training 398 (70%), validation 85 (15%), testing 85 (15%)			283.15 - 473.15		no
Golzar et al.[38]	11 different ILs mixtures	ANN		Total 884 data points; training 70%, validation 15%, testing 15%	0.98 - 1.21 $g \cdot cm^{-3}$				no
Barati-Harooni et al.[39]	146 ILs	RBF-NN model	T, Mw (g/mol), Tb (K), Tc (K), Pc (Pa), Vc ($cm^3 \cdot mol^{-1}$), critical compressibility factor (Zc) and acentric factor (w)	Total 602 data points; training 80%, testing 20%	0.9 - 1.66 $g \cdot cm^{-3}$		278 - 358	R^2 0.9916, RMSE 0.0139	no
Pan et al.[40]	573 distinct EILs	KRR, SVR, GBR, RF, DT, MLP, stacking models		Total 573 EILs				R^2 0.96, MAE 0.034 $g \cdot cm^{-3}$	no
Golzar et al.[41]	MDEA, PZ and ILs aqueous solutions as single, binary and ternary	ANN	weight fractions, molecular weight, acentric factor and T and P	Total 272 data points; training 70%, validation 15%, testing 15%		0.177 - 3938.40	293.15 - 373.15	R^2 0.999, RMSE 0.006	no

	aqueous solutions								
Taghizadehfard et al.[42]	28 AAILs	ANN	Molecular weight ($g \cdot mol^{-1}$), liquid molar density ($mol \cdot m^{-3}$) at 298 K, reduced temperature	Pure ILs total 344 data points; training 80%, validation 10%, testing 10% Mixtures total 279 data points			283 - 373.15	AAD 0.26%, 0.025%: pure and mixtures	no
Kovacevic et al.[43]	49 ILs	PCA, SS, MLR and ANN	250 molecular descriptors PaDEL ALOGPS 2.1 descriptors	MLR: Training 42 ILs, testing 8 ILs. ANN: training 25 ILs, testing 4 ILs, validation 4 ILs				MLR: R^2_{cv} 0.9047	no
Ma et al.[44]	25 classes of ILs	ANN, XGBoost, and LightGBM	IL's cation, its substituents, the anion, the cation and anion of the IC, along with the temperature and molar fraction of the IL	Total 5,738 data points; training 80%, validation 10%, testing 10%			283 - 373.15	XGBoost-GC R2 0.9983, MAE 3.0694	no
Paduszynski et al.[45]	2267 ILs	MLR, FF ANN, and least-squares SVM		Total 41,250 data points	800 – 2,800 $kg \cdot m^{-3}$	0.1 - 300 MPa	220 - 473	AARE 0.90%	no
Shu et al.[8]	54 IL-organic solvent-organic solvent	ANNs, XGBoost, LightGBM		Total 6,515 data points; training 4,555, validation 981, testing 981	749.2-1,379.9 $kg \cdot m^{-3}$		293 - 353	MAE 7.3760, ANN R^2 0.9943	no
Valderrama et al.[46]		ANN	GC method	Total 481 data points; training 399 (103 ILs), testing 82 (24 ILs)			278 - 358	AAD 0.26%	no
El-Harbawi et al.[47]	747 pure ILs	MLR and polynomial equation		Total 918 data points				R^2 0.995	no

Venkatraman et al.[48]		GBM	KRAKENX descriptors	Calibration 9,224 (67%), test 7,730 (33%)	1060 – 1,457 $kg\cdot m^{-3}$			GBM R^2 0.93	no
Lei et al.[49]	28 classes	ANN, XGBoost, and LightGBM	GC	Total 10,553 data points; training 70%, validation 15%, testing 15%			288.15 - 433.15	ANN R^2 0.998, MAE 0.612	no
Umaña et al.[50]	218 ILs	Neural network	Pubchem, rdkit descriptors	Total 2,371 data points	934.7-1,625.9 $kg\cdot m^{-3}$		273 - 363	R^2 0.96	Link
Baran et al.[51]	2000 distinct ILs	Graph neural networks	sigma partial charges, MMFF94 partial charges, Formal charges, QTPIE partial charges	Total 40,000 points; training 75%, validation 15%, testing 10%				R^2 0.99, RMSE 27.3, and MARE 0.015	Link
Vakili-Nezhaad et al.[52]	[BMIM][SCN], [BMIM][BF4], [HMIM][BF4], [HMIM][PF6]	adaptive neuro-fuzzy inference system (ANFIS)		Total 1,670 data points	1.07254 - 1.29145 $g\cdot cm^{-3}$		220 - 472	R^2 0.968, RMSE 7.274, and AARD 0.368%	no
Keller et al.[11]	58 unique ILs, 13 unique cations and 25 unique anions	SVR and GBR	GC features	Total 549 data points			293 - 363	AARD 1.0%. R^2 0.961 and 0.968	Link
Golparvar et al.[53]	6 ILs	SVM		Total 731 data points; training 548, testing 183			293.15 - 328.15	R^2 0.999	no
Liu et al.[12]	17 ILs	ANN, XGBoost, and LightGBM		Total 34,754 data points; training 24,282, validation 5,137, testing 5,335				ANN MAE 0.0049, R^2 0.9942	no

Torrecilla et al.[54]		ANN	polarizability of the anions, temperature	Total 39 data points; training 70%, validation 20%, testing 10%			293.15 - 353.15	MPE 0.06%	no
Baskin et al.[55]	1204 ILs	RFR, XGBoost, ASNN, DNN, CP-MPNN, CNF, TransCNF, GIN, TransCNN	ISIDA-Fr, MolD2, CDK23, and Dragon7	Total 47,267 data points				DNN Q^2: 0.95, RMSE 0.042	no
Golzar et al.[56]	quaternary ammonium-based ILs ([N222(n)]Tf2N with n 5, 6, 8, 10, 12)	GFA and ANN models	Tc, W, and T	Training 70%, validation 15%, testing 15%	1.16 - 1.29 g·cm^{-3}		298 - 373	R^2 0.9998, RMSE 0.007	no
Fu et al.[13]	288 ILs	ANN, XGBoost and LightGBM		Total 21,674 data points; training 15,248, validation 3,235, testing 3,191				ANN-GC: R^2 0.9130	no
Lashkarbolooki et al.[57]	250 ILs	ANNs		Total 426 experimental data			293.0 - 328.0	AARD 0.85%, ARD 0.05, R^2 0.9948	no
Nordness et al.[58]	39 ILs imidazolium ILs	SVR	COSMO-RS sigma profiles	Total 2,847 data points; training 80%, testing 20%		100 - 300,000	219.92 - 473.15	R^2 0.994, AARD 0.3%	no
Makarov et al.[59]	2581 ILs	TransCNN, TransCNF, Consensus		Training set 27,082 datapoint (2,251 ILs), Test 3,944 data points (330 ILs)			298 - 523	R^2 0.990 ± 0.00, RMSE 18.6 ± 0.8	no
Lemaoui et al.[60]	2100 ILs (135 IL families)	MLR, MNLR, DT, RF, GBM, kNN, SVM, ANN	COSMO-RS Sigma profile, molecular features	Total 40,860 data points	849 - 2,800 kg·m^{-3}	60 - 300,000	217 - 573	ANN R^2, 0.993	no

Cancilla et al.[61]		ANNs		Total 112 samples; training 70%, validation 20%, testing 10%			303.15 - 353.15	MPE 0.09%	no
Firoozirad et al.[16]	Binary mixture of one IL [C2mim][CH3SO3] + water	ANN		Total 30 datasets; training 70%, validation 15%, testing 15%	1.2287 - 1.2844 g·cm^{-3}		298.15 - 333.15	R^2 0.9991, MAE 0.00278 g·cm^{-3}, RMSE 0.0007 g·cm^{-3}	no
Palomar et al.[62]	45 imidazolium-based ILs	LR, MLP, NN	COSMO-RS Descriptors, sigma profile	Training 90%, testing 10%			298	R^2 0.995, MPE 0.8%	no
Wang et al.[17]	26,404 datapoint (995 ILs)	KNN, SVR, RF, GBRT, XGBoost		Training 80%, testing 20%	1.35·10^{-3} - 2.15 g·cm^{-3}	92.30 - 110.00	101.33 - 473.15	XGBoost R^2 0.981, RMSE 0.0235, MAE 0.0148 g cm^{-3}	no
Rostami et al.[63]	747 ILs	GC-LSSVM	T, P, and 47 substructures, COSMO-RS	Total 918 data points; training 689 (75%), testing 229 (25%)				MSE 0.0004, R^2 0.9925	no
Song et al.[64]	674 ILs (15 classes'9	GNN, DMPNN, FFNN	RDKit descriptors	Total 16,605 data points			280 - 350	Regression coefficient 0.9999	no
Li et al.[65]	333 cations, 120 anions, 711 ILs	Ionic Fragment Contribution (IFC) with GNNs	99 ionic fragment molecular graph features	Total 16,954 data points	836.1 – 1,852.6 kg·m^{-3}	1 – 2,000 bar	101.3 - 571.3	MAE 2.3431, R^2 0.99, RMSE 7.2365	Link

Ma et al.[66]	83 ILs	MLP, RF, CNN, Ensemble Model	418 Rdkit descriptor	Total 15,826 data points; training 80%, testing 20%	700 – 1,600 kg·m^{-3}		278.15 - 353.15	R^2 0.9945, MAE 0.0063, RMSE 0.0113	no

Table S3. Literature-Summary of Machine Learning Studies for the Prediction of Viscosity in Ionic Liquids

Reference	Families of ILs	ML Models	Descriptors	Size of Dataset	Viscosity, mPa·s	Range of Pressure, kPa	Range of Temperature, K	Performance	GitHub Link
Carrera et al.[67]	pure ILs, mixtures of two ILs and mixtures of an ILs and a molecule	RF and Kohonen neural network	MOLMAP descriptors	Training 6,937 systems, 2 validation sets: 1st set 767 systems, 2nd set 366 systems	Class A: 0.28-20.59, Class B: 20.6-51.6, Class C: 51.7-122.9, Class D: 123-414, Class E: 415-1,035.5 and Class F: 1,036-140,000	0.07 - 300	253 - 438	Classification of 6 groups Accuracy 74.61 %	no
Carrera et al.[68]	13798 samples including single IL systems, mixtures IL/IL and IL/Organic compound	RF	MOLMAP descriptors	Total 8,501 samples; training 7706 systems, validation sets, 1st set 795 systems, 2nd set 366 systems	Class A: 0.28-20.59, Class B: 20.6-51.6, Class C: 51.7-122.9, Class D: 123-414, Class E: 415-1,035.5 and Class F: 1,036-140,000	0.07 - 300	253 - 438		no
Kianfar et al.[35]	pyridinium-based hydrophobic ILs	ANN	normalized temperature	Total 48 data points; training 36, testing 12	9.19 - 276.16		278.15 - 353.15	MSE $1.1332 \cdot 10^{-4}$, R^2 0.9982	no
Bendimerad et al.[32]	1,974 ILs	Logistic Regression, SVM, RF, XGBoost	Mordred	1,762 features					no
Lashkarblooki et al.[69]	5 ILs	ANN, MFFANN	non-ILs components compositions,	Total 729 data points: training 547, testing 182				AARD 1.96%, R^2 0.999	no

			M, boiling point T						
Golzar et al.[41]	MDEA, PZ and ILs aqueous solutions as single, binary and ternary aqueous solutions	ANN	X_{IL}, Mw, acentric factor, T, P	Total 686 data points; training 70%, validation 15%, testing 15%		0.177- 3,938.4	293.15 - 373.15	R^2 0.989, RMSE 0.758	no
Díaz-Rodrígue z et al.[70]	4 ILs binary mixtures	ANN-MLP	T, X_{IL}, Mw mixture	Total 639 data points			298.15 - 308.15	MPE 1.5%	no
Moosavi et al.[71]	[bmim]PF6	ANN		Total 1,008 data points	75.3 - 264.5		283.15 - 333.15	RMS 0.026368	no
Fatehi et al.[72]	59 different ILs	ANN	T, P, IL structure	Total 736 data points; training 612, testing 124		0.1 - 15	273.15 - 393.15	AAE 1.310%.	no
Moslehi et al.[73]	20 pure ILs	ANN model		Total 3,074 data points			298.15 - 353.15	AAD 1.44%	no
Paduszynski et al.[74]	1,484 ILs	Feed-Forward ANN	242 GC-type molecular descriptors	Total 13,470 data points; training 90%, validation 5%, testing 5%	0.41 – 257,000	0.06 - 350	253 - 573	AARD% 11.4%, R^2 0.984	no
Díaz-Rodrígue z et al.[26]	two binary mixtures ([bpy][BF4]+[bpy][Tf2N] and [bpy][BF4]+[4bm py][Tf2N])	ANN	composition (X_{IL}), T	Total 156 data points			273.15 - 353.15	MPEs 3.24%	no
Golzar et al.[38]	IL + water/alcohol binary mixtures	ANN		Total 496 samples; training 70%, validation 15%, testing 15%	0.546 - 20,883		278.15 - 348.15	R^2 0.9572, RMSE 1.5831, AAD 1.09%	no

Beckner et al.[75]	imidazolium, phosphonium, pyridinium, and pyrrolidinium	ANN	633 RDKit descriptors	Total 578 data points	0.0035 - 0.993 Pa s	60 - 160	273.15 - 373.15	Relative AAD 7.1 ± 1.3%	no
Mousavi et al.[76]	45 different ILs	MLP-LMA, MLP-BR, LSSVM-BAT, DT	T, P, Mw, Vc, Tb, Tc, Pc, ω, and chemical structure	Total 2,813 data points; training 80%, testing 20%	1.13 - 9,667.6	0.06 - 298.9 MPa	293 - 393	RMSE 6.844, R^2 0.999, AARD% 3.454	no
Chen et al.[19]	6 ILs family (133 different IL-IL binary mixtures)	ANN, XGBoost, LightGBM		Total 6,141 data points; training 5,207, testing 934			263.64 - 533.2	R^2 0.9999, MAE 0.000321	no
Cao et al.[77]		MLR, RR, ANN, SVR, RF, and XGB					253.15 - 573.00	R^2 0.999	no
Fatehi et al.[78]	binary mixtures of ILs	FFNN	Mw, group structure, reduced boiling temperature, X_{IL}, T, P	Total 1,996 data points; training 1,775, testing 221			283.15-353.15	AAE 5.4%	no
Sahebalzamani et al.[22]	binary mixture of 0.75 IL and 0.25 water	CFNN and GRNN models	T(K) and shear rate ($1 \cdot s^{-1}$)	Total 133 data points; training 80%, testing 20%	0.047 - 0.246 Pa·s		283.15 - 333	CFNN-LM AAPRE 0.28, R^2 0.996	no
Soman et al.[79]	[Bmim][PF6] ionic liquid + water	ANN	Concentration, T		0.948 - 1.31		296 - 336	RMSE 0.014	no
Mazari et al.[20]	[Bmim][PF6]	three GPR models, SVM			190 - 1,000	0.1-175 M Pa	273 - 388	R^2 0.9884548, AARD% 10.7729	no
Chen et al.[80]	153 ILs-water mixtures	ANN-GC Model		Total 8,523 data points	$0.334 \cdot 10^{-3}$ - $20{,}900 \cdot 10^{-3}$ Pa·s		272.10 - 373.15	MAE 0.0095, R^2 0.9952	no

Huwaimel et al.[81]		RF, GB, XGBoost	Type of cation, Type of anion, T, xIL (mol%)	Total 8,500 data points; training 80%, testing 20%			280 - 373	RF R^2 0.9971, AARD 6.25%	no
Dutt et al.[82]	81 ILs	ANN		Total 736 data points; training 654 (73 ILs), testing 82 (8 ILs)	1.22 - 32.5		323.15	R^2 0.90	no
Valderrama et al.[83]	84 ILs	ANNs	T, ρ, M^+, M^-, λ	training 327 data points (31 ILs), testing 31 data points (26 ILs)	5,222 cP - 257,000 cP			AAD 1.68%	no
Zhao et al.[84]	45 imidazolium-based ILs	MLR, SVM	six parameters, T, M, Vc, P, Tb, and Zc	Total 1,079 data points; training 865, testing 214	7.75 - 142,000 cP	1 - 3,000 bar	273.15 - 395.32	SVM R^2 0.977, AARD 3.95%, MSE 0.009	no
Zimmermann et al.[85]	Ten ammoniums ILs	Feedforward and cascade forward neural networks		Total 2,499 data points; training 70%, validation 15%, testing 15%	0.5706 - 5,118.10		283.15 - 343.15	MSE 42.2915, AARD 0.1999% R^2 0.9999	no
Fan et al.[86]	55 ILs	SVR, RF, ANN, LightGBM, XGBoost, CatBoost		Total 807 data points	4.229 - 140,000		253.15 - 398.15	R^2 0.9999, MAE 0.0325	no
Ding et al.[87]		RF, SVM, MLR	GCM	Total 1,806 data points	8.28 - 142,000		253.15 - 433.15	R^2 0.989, RMSE 0.053	no
Firoozirad et al.[16]	Binary mixture of one IL [C2mim][CH3SO3] + water + different mass concentrations of	ANN		Total 66 datasets; training 46, validation 10, testing 10			298.15 - 333.15	R^2 0.9961, MAE 0.01988 Pa s, RMSE 0.081 Pa s	no

	alumina nanoparticles								
Nordness et al.[58]	imidazolium ILs, 40 different ILs	SVR	COSMO-RS sigma profile	Total 1,061 data points; training 552, testing 121	7.7 - 117,000	100 - 300,000	273.15 - 395.32	R^2 0.955, AARD 10.2%	no
Baskin et al.[55]	988 ILs	TransCNN, NN (7 different architectures), Transformers, RFR, XGBoost		Total 20,311 data points			288 - 343	Q^2 0.806, RMSE 0.289, MAE 0.191	no
Boualem et al.[88]	1,654 different ILs (21 and 15, cations and anions)	group contribution, NLR, SVM		Total 15,251 data points	0.841 - 128,800		253.15 - 573	GC/SVM AARD 9.07%	no
Huang et al.[89]	41 ILs	KNNR, RF, XGBoost, AdaBoost and CatBoost	208 RDKit descriptors	Total 2,676 total points			272.10 - 373.15	Catboost: RMSE 0.0084, MSE 0.0001, MAE 0.0020, R^2 0.9941	no
Makarov et al.[59]		DNN, RFR		Total 2,646 data points					no
Keller et al.[11]	15 unique cations, 26 unique anions, 59 unique combinations	SVR and GBR	GC-based descriptors	Total 527 data points			290 - 373	AARD 12.5%, R^2 0.989	Link
Lemaoui et al.[60]	135 ILs families (2,026 ILs)	RF, kNN, MLR, SVM, ANN, GBM, and DT	Sigma-profiles	Total 25,243 data points; training 20,961 (1,717 ILs), testing 4,282 (309 ILs)	2 - 442.413			R^2 0.9071	no

Gao et al.[90]	Anions: tetrafluoroborate [BF4]⁻, chloride [Cl]⁻, dicyanamide [DCA]⁻ etc. Cations: imidazolium, pyrrolidinium, pyridinium	GNNs, XGBoost	SMILES, morgan fingerprint, chemical descriptors	Total 10,733 data points; training 80%, testing 20%		0.6 - 3,000 bar	253 - 573	R^2 0.952, MAE 0.096	no
Zhong et al.[91]		CatBoost, CNN	molecular fingerprint, molecular descriptor, molecular image, and molecular graph 159 features	Total 1,420 data points; training 80%, testing 20%	0.58778 - 2.11360	101.325	269.96 - 286.03	R^2 0.906, MAE 0.304, RMSE 0.500	Link
Lei et al.[49]	15 classes of ILs	ANN, XGBoost, LightGBM	GCM	Total 3,581 data points; training 3,078, testing 503	0.000595 - 1.953		288.15 - 433.15	ANN - GC R2 0.985	no
Paduszynski et al.[92]	2,068 distinct ILs	SWMLR, FFANN, LSSVM		Total 3,236 data sets and 22,268 data points	0.7 - 257,000		290 - 370	SWMLR, FFANN, LSSVM: R^2 0.8436,0.9092, 0.9172	Link
Wang et al.[17]	756 ILs	KNN, SVR, RF, GBRT, and XGBoost		Total 16,123 data points; training 80%, testing 20%	0.84 - 220,000.00	90.00 - 110.00	222.20 - 573.00	KNN R^2 0.9578, RMSE 0.3219, MAE 0.1606	no
Baran et al.[51]		GNNs	Graph representations with electrostatic	Total 20,000 data points; training 75%, validation 15%, testing 10%				R^2 0.84, MARE 0.377	Link

			information (Gasteiger, MMFF94, formal, QTPIE charges)						
Mohan et al.[93]	967 ILs, 419 cations and 172 anions	SVR, FFNN, CATBoost	RDKit and Mol2vec chemical structure descriptors, COSMO-RS sigma profile	Total 11,180 data points	0.97 - 1,040,000	60 - 950,000	253.15 - 573	CATBoost R^2 0.984: AARD 2.75%	no
Torrecilla et al.[54]	Choline carboxylate IL	NN		Total 39 different data points; training 70%, validation 20%, testing 10%			293.15 - 353.15	MPE 2.33%	no
Billard et al.[94]	99 ILs	QSPR ANN		99 ILs; training 80%, testing 20%, external test set 23 new ILs	3 - 800 cP			R^2 0.73, RMSE 67.5 cP	no
Kiani et al.[95]	45 various ILs	genetic programming (GP) and group method of data handling (GMDH) models	T, P, Mw, Vc, Tb, Tc, Pc and w	Total 2,813 data points; training 80%, testing 20%	1.13 - 9,667.6	0.06 - 298.9	253.15 - 573	AARD 8.14%, R^2 0.98	no
Sheikhshoaei et al.[96]	Imidazolium-based IL mixtures	RF, KNN, GPR, CatBoost, LightGBM		Total 4,952 data points	1.13 - 8,278.0		253.15 - 573.0	RF R^2 0.995, AAPRE 3.97	no

Zhao et al.[97]	45 various ILs	Genetic Programming (GP), Group Method of Data Handling (GMDH)	T, P, Mw, Vc, Tb, Tc, Pc and w	Total 2,813 data points; training 2250 (80%), testing 563 (20%)	1.13 - 9,667.6	0.06 - 298.9	253.15 - 573	AARD 8.14%, R^2 0.98	no
Cancilla et al.[61]	112 samples	ANN		112 samples; training 70%, validation 20%, testing 10%			303.15 - 353.15	Average MPE 4.92%	no
Venkatraman et al.[48]		GBM	OpenBabel 100 Descriptors	calibration (67%), validation (33%)	43 - 1,848			R^2 0.83, AARD 0.74%, RMSE 0.017	no
Umaña et al.[50]	218 ILs	GNN, ANN	Experimental properties, computational molecular features, screened ion pair interaction energy, 3D RDKit descriptors	Total 2,224 data points	4.9 - 11,500		273 - 363	R^2 0.72	Link
Duong et al.[98]	83 unique ILs	K-means clustering, MLREM (multiple linear regression with expectation maximizatio	Avogadro descriptor	Total 1,827 data points; training 80% testing 20%	0.0258 - 5,647 cP		293 - 403	Q^2 (0.917 MLREM, 0.89 BRANNLP) SEP (0.310, 0.365 cP, MLREM, BRANNLP)	no

		n) and non-linear algorithm BRANNLP (Bayesian regularized ANN with Laplacian prior)							
Ma et al.[44]	12 classes of ILs (imidazolium, ammonium, and piperidinium)	GC - ANN, XGBoost, and LightGBM		Total 1,551 data points; training 80%, testing 20%			283 - 373.15	ANN-GC model R^2, 0.9967	no
Golzar et al.[56]	1-Methyl-3-octylimidazolium chloride with alcohols	ANN, GFA		Training 70%, validation 15%, testing 15%	10.17 - 311.8		298 - 373	ANN R^2 0.998, RMSE 2.597, AAD 0.51%	no
Shu et al.[8]	34 IL-organic solvent-organic solvent	ANNs, XGBoost, and LightGBM		Total 2,775 data points; training 1,949, validation 414, testing 414	0.1 - 153.8		288.15 - 363.15	ANN model MAE 0.0225, R^2 0.9973	no
Han et al.[99]		GPR, MLP, SVR	T and xIL (mol%), cation and anion	Total 8,500 entries				R^2 0.99765, MAE $1.73330\cdot10^{-3}$, RMSE - $2.2317\cdot10^{-3}$	no
Li et al.[65]	841 cations, 223 anions, 1762 ILs	Ionic Fragment Contribution (IFC) with GNNs	85 ionic fragment molecular graph features	Total 8,589 data points	0.02 - 6.85 (log10(η) in mPa·s)	1 - 3,000 bar	253 - 573	MAE 0.0667, R^2 0.95, RMSE 0.1524	Link

Table S4. Literature-Summary of Machine Learning Studies for the Prediction of CO_2 Solubility in Ionic Liquids

Reference	Families of ILs	ML Models	Descriptors	Size of Dataset	CO_2 Solubility (mole fraction)	Range of Pressure, kPa	Range of Temperature, K	Performance	GitHub Link
Valeh-e-Sheyda et al.[100]	11 pyridinium-based IL mixtures	MLFNN	five input variables	Total 430 data points	0.0017 - 0.5751		298.15 - 343.15	R^2 0.995	no
Laakso et al.[101]	124 ILs	MLR, ANN, RF	Sigma profile COSMO-RS	Total 9,864 data points; training 80%, testing 20%	0.0000648 - 0.9516	0.798 - 49,990	243.15 - 453.15	RF R^2 0.9754, MAE 0.0257	no
Li et al.[102]	126 ILs	SVM, RF, MLP, KNN, DTR, GBR	T, P, M, anion descriptors, cation descriptors	Total 10,174 data points; training 80%, testing 20%	0.00 - 0.96	0.007 - 500 bar	243 - 455	MAE 0.002 R^2 0.980	Link
Sheikhshoaei et al.[103]	imidazolium-based ILs	Bayesian Neural Networks (BNN), DNN, Gradient Boosting Neural Networks (GrowNet), Tabular Neural Networks (TabNet), RF, SVR	Mw, T, P, Tc, Pc, ω	Total 963 data points	0.0034 - 0.64	24.59 - 13,819	273.15 - 413.15	GrowNet model MSE 0.0054%, MAE 0.5324, RMSE 0.0073%, coefficient of determination 0.9962	no
Liu et al.[104]	103 ILs	MLP, RF, SVM	Rdkit features 338	Total 9,224 data points	0.0000648 - 0.9516	0.826 - 49,990	243.2 - 453.15	SE-MLP R^2 0.9873, MSE 0.0007	no
Deng et al.[105]	13 ILs	CP-DNN, CP-CNN, CP-RNN, XGBoost	M, Tc, Pc, ω, T, P	Total 218 data points; training 70%, testing 30%				R^2 CP-RNN 0.988, CP-CNN 0.999, and CP-DNN 0.984	no

Song et al.[106]	124 ILs	ANN-GC and SVM-GC	GC	Total 10,116 data points; training 8,093 (80%), testing 2,023 (20%)	0.0000648 - 0.9516	0.00798 - 499.9 bar	243.2 - 453.15	ANN-GC, MAE 0.0202, R^2 0.9836	no
Bastami et al.[107]	296 ILs	MLP, RBF, RF, LSBoost	239 and 304 molecular descriptors for cation and anion structures	Total 16,480 data points	0.0000018 2 - 0.9516	0.00001 - 105.01 MPa	271.11 - 453.15	LSBoost model: R^2 and MSE: 0.9243 and 0.1277	no
Rozhenko et al.[108]	43 imidazolium-based ILs	XGBoost, LightGBM, CatBoost, Adaboost-SVR	26 input variables	Total 4,397 data points	0.005 - 0.142	0 - 95 bar	298 - 353	XGBoost: R^2 0.999 and RMSE 0.0077	no
Torrecilla et al.[109]	5 ILs	MLR, Multiple Quadratic Regression (MQR), Radial Basis Network (RB), MLP, NN	T, P			0.01 - 12.88 MPa	278.47-368.22	MPE value less than 3.3%	no
Zhao et al.[110]	103 ILs	RF	2,051 molecular features	Total 9,188 data points; training 80%, testing 20%	0 - 0.952	0.883 - 49,990	243.20 - 453.15	R^2 0.982	no
Valeh-e-Sheyda et al.[111]	43 imidazolium-based ILs	FFNN, RBF-NN, SVM		Total 4,397 data points; training 3,078 (70%), validation 659 (15%), testing 660 (15%) 660	0 - 0.9150	0.0001 - 1,001.2 bar	278.12 - 450.49	R^2 0.952 MAPE 0.544	no
Benmouloud et al.[112]	124 different ILs	ANN, SVM optimized with dragonfly algorithm (DA-SVM)	13 significant PaDEL descriptors (E2M, MATS8S, TDB6I, TDB1S, ATSC4V, MATS8M,	10,116 data points, training set (8093 data points), test set (2,023 data points)	0.0000648 -0.9516	0.00798 - 499.9 bar	243.2 - 453.15	ANN MAE 0.0195, R^2 0.9828	no

			ATSC7V, Gats2S, Gats5S, Gats5C, ATSC6V, DE, and Lobmax)totalt						
Yang et al.[113]	110 ILs	XGBoost models FNN	GC, P, T, Chi2v, Chi0n, and BertzCT, 21 descriptors	Total 9,455 data points	0.0000648 - 0.9516	0.00798 - 499.9 bar	243.2 - 453.15	R^2 0.98963, MAE 0.01480, RMSE 0.02369	no
Qin et al.[114]	72 ILs	XGBoost	COSMO-RS	Total 3,036 data points	0.00116 - 0.713	9.7 - 6,532.8	273.15 - 413.15	AARD 0.94%, R^2 0.599	no
Tian et al.[115]	164 kinds of ILs	ionic fragments contribution (IFC) with SVM and ANN		Total 13,055 data points; training 10,444, testing 2,611	$3.8 \cdot 10^{-6}$ to 0.9516	0.0002 - 499.9 bar	243.2 - 453.15	R^2 0.9763, MSE 0.0013, MAE 0.0192	no
Jun et al.[116]	124 different ILs	MPNN-based		Total 10,116 data points	0.0000648 - 0.9516	0.00798 - 499.9 bar	243.2 - 453.15	MAE 0.0134, R^2 0.9902	no
Aghaie et al.[117]	46 ILs	LSSVM, DT, RF, MLR	T, P, Tc, Pc, ω, Mw		0.0000066 - 0.965	0.01 - 94,600	283 - 450.5	RF R^2 0.96 AARD 12.05%, 12.36%	no
Tatar et al.[118]	14 different ionic liquids	RBF-NN, LSSVM, MLP		Total 728 data points; training 582, testing 146	0.00175 - 0.824	9.7 - 100,120 MPa	298.15 - 373.15	AAED 7%	no
Rahimi et al.[119]	7 ILs	ANNs, RF		Total 173 data points; training 153 (80%), testing 20 (20%)	0.14 - 0.62	96.6 - 19,007.9 bar	298.15 - 343.15	RF R^2 0.9998	no
Agwu et al.[120]	20 ILs imidazolium-based ILs	ANN	T, P, physical properties of the ionic solution (critical pressure of ILs, acentric factor)	Total 1,517 data points; training 1,061 (70%), validation 228 (15%), testing 228 (15%)	0.00182 - 0.853	0.0098-72.24 MPa	292 - 450	R^2 0.992, RMSE 0.04, MSE 0.00173	no

Geng et al.[121]	124 ILs	CatBoost, LightGBM, XGBoost, GBDT, RF and AdaBoost	group contribution method (GC)	Total 10,116 data points; training 80%, testing 20%				CatBoost R^2 0.9945, MAE 0.0108	no
Gao et al.[90]	74 anions 78 cations	GNN, XGBoost		Total 24,067 data points		0.01 - 1051.1	243.2 - 453.15	R^2 0.971, MAE 0.023	no
Liu et al.[122]	20 ILs	SVR, Particle swarm optimization (PSO), Grey Wolf optimization (GWO), Sparrow search algorithm (SSA)	T, P, Tc, Pc, acentric factor (ω)	Total 1,517 data points	0.002 - 0.853	0.0098 - 72.24 MPa	292.65 - 450.49	RMSE 0.01881, R^2 0.9824	no
Yang et al.[123]	124 ILs	GPR, LightGBM, and CatBoost	molecular structure descriptor (MSD)	Total 10,116 data points; training 80%, testing 20%	0.0000648 - 0.9516	0.00798 - 499.9 bar	243.2 - 453.15	CatBoost-GC-MSD model R^2 0.9925, MAE 0.0122, MRE 11.1550%	no
Mesbah et al.[124]	20 ILs	MLP-NN		Total 1,386 data points; training 70%, validation 15%, testing 15%	0.02 - 0.82	0.25 - 100.12 MPa	278.15 - 450.49	R^2 0.9987, MSE 0.6293, AARD 1.8416%	no
Song et al.[64]	123 ILs	GNN		Total 9,338 data points				DMPNN R^2 0.988	no
Ali et al.[125]	164 ILs	ANN	T, P	Total 10,116 data points; training 8,093 (80%), testing 2,023 (20%)		0.00798 - 499 bar	243.2 - 453.15	R^2 0.986, RMSE 0.0273	no
Moosanezhad-Kermani et al.[126]	67 ILs	GMDH algorithm	T, P, Tc, Pc, and acentric factor (ω)	Total 4,726 data points; training 3,781, testing 945, external testing 642	0.0001 - 0.968	0.0089 - 100.12	271.11 - 453.15	R^2 0.9072, RMSE 0.0759	no
Baghban et al.[127]	67 ILs	LSSVM, MLP-ANN, RBF-ANN, ANFIS	T, P, Tc, Pc, and acentric factor (ω)	Total 5,368 data points; training 4,026 (75%), testing 1,342 (25%)	0.0001 - 0.9681		278.12 - 453.15	LSSVM R^2 0.9942, MSE 0.00035	no

Balchandani et al.[128]	19 (ILs+alkanolamine + water/ethanol)	MLR, ANN	Tc, Pc, ω, activity coefficient, fugacity coefficient	Total 278 data points; training 28 data sets, testing 22 data sets	0 - 0.487	50 - 6,200	298 - 353	AAD 0.00624%	no
Chen et al.[129]	213 ILs	VAE-ANN-PSO		Total 15,666 data points; training 90%, testing 10%	0 - 0.9516	0 - 5,000	300 - 375	R^2 0.979, MAE 0.022	Link
Fierro et al.[130]	33 ILs	ANN	T, P, Tc, Pc	Total 2,099 data points; training 1,890 (90%), validation 104 (5%), testing 105 (5%)	0.1000 - 0.8456	0.010 - 100.120 MPa	273.15 - 449.41	absolute deviation 4.38%	no
Davoodi et al.[131]	124 ILs	CNN, LSTM	P, T, cation type, and anion type	Total 10,116 data points; training 80%, testing 20%	0.000065 - 0.9516	0.00798 - 499.9 bar	243.2 - 453.1	R^2 0.9953, RMSE 0.0164	no
Mirarab et al.[132]	[pamim][Ala]	ANN, MLP		54 data sets; training 38 data (70%), validation 8 data (15%) testing 8 data (15%)			298 - 333	R^2 0.998	no
Golzar et al.[41]	12 ILs and MDEA, PZ	ANN	w, Mw, ω, T, P	Total 1,371 data points	0.0 - 1.0	0.177 - 3938.400 kPa	293.15 - 373.15	R^2 0.998, RMSE 0.059	no
Ghiasi et al.[133]	66 ILs	RBF-ANN, MLP-ANN, ANFIS and LSSVM models	T, P, Tc, Pc, ω	Total 5330 data points; training 90%, testing 10%	0.0001 - 0.9681	0.0089 - 100.12 MPa	271.11- 453.15	AARD% 0.04	no
Mazari et al.[20]	aqueous and non-aqueous [Bmim][PF6]	GPR (Matern 5/2 GPR), SVM	T, CO_2 partial pressure and water wt%			0.015 - 9.685 MPa	293 - 395	R^2 0.9676177, ARD% 1.7029, AARD% 5.7391	no

Venkatraman et al.[134]	185 ILs	Partial Least Squares Regression (PLSR), RFs , Conditional Inference trees (CTREE)	Semi-empirical (PM6) electronic, thermodynamic and geometrical descriptors from KRAKENX software	Total 10,848 data points	0.000 - 0.951	0.000 - 95.700 bar	271 - 453	RF R^2 0.85, RMSE (MAE), 0.10 (0.003)	no
Hamzehie et al.[135]	51 single and blended alkanolamine, IL and amino acid salt solutions	MLFNN, ANN		Total 2,982 data points; training, 2,089, validation 305, testing 588		0 - 20,000	293 - 478.15	ANN R^2 0.9984, MSE $3.7468\cdot10^{-5}$, ARD 2.7992	no
Hamzehie et al.[136]	32 single and mixed amine and IL solutions	ANN	T, solute's concentration, acid gas partial pressure, solution Tc, solution Pc, MW	Testing 114 data points	0.038 - 1.0	13 - 9319	298 - 434.5	MRE 4.143, MSE $1.011\cdot10^{-4}$, R^2 0.998	no
Li et al.[65]	78 cations, 74 anions, 216 ILs	Ionic Fragment Contribution (IFC) with GNNs	48 Ionic fragment molecular graph features	Total 10,897 data points	0 - 0.9516	0 - 1,050.1 bar	271.11 - 453.15	MAE 0.0187, R^2 0.98, RMSE 0.0338	Link

Table S5. Literature-Summary of Machine Learning Studies for the Prediction of Melting Point in Ionic Liquids

Reference	Families of ILs	ML Models	Descriptors	Size of Dataset	Melting Point, K	Performance	GitHub Link
Bini et al.[137]	pyridinium bromides	RNN		Total 126 data points; training 100, testing 26	<333	correlation coefficients (R) 0.8725, MAE: 19.37 K	no
Wang et al.[138]		SVM		Total 768 data points; training 600, testing 168		AARD 5.12%, R^2 0.8542	no
Feng et al.[139]	3,080 ILs	eight GNN, SVM, RF, and MLP	ECFPs descriptors	Training 80%, validation 10%, testing 10%	178 - 592	RMSE 37.06, MAE 28.79, R^2 0.76	Link
Varnek et al.[140]	Bromides of nitrogen-containing organic cations (pyridinium, imidazolium, benzoimidazolium, quaternary ammonium)	QSPR Modeling, Associative Neural Networks, SVMs, kNN, PLSM, BPNN, and MLR	E-state indices, molecular descriptors (DRAGON), substructural molecular fragments	Total 717 data points (PYR: 126, IMZ: 384, QUAT: 207)		RMSE 37.5 - 46.4 °C (FULL), 26.2 - 34.8 °C (PYR), 38.8−45.9 °C (IMZ), and 34.2−49.3 °C (QUAT)	no
Fatemi et al.[141]	62 ILs, ammonium, pyrrolidinium, imidazolium, pyridinium, piperidinium, phosphonium ILs salts	QSPR, LR, MLR, MLP NN	structural descriptors			Training, Test and validation sets: MLR 0.91, 0.86 and 0.79, MLP NN: 0.97, 0.96 and 0.85	no
Lazzús et al.[142]	63 ILs, 10 cations, 21 Anions	MLR		Training 40, testing 23	185 - 466	AARD 5.3%	no
Lazzús et al.[143]	200 ILs	genetic algorithm-based on MLR		Total 200 data points	212-528	average deviation of 7%, R^2 of 0.6-0.9	no

Qu et al.[144]		Monte Carlo Tree Search, Recurrent Neural Network, RF regression, XGBoost regression, Ridge regression, and ANN	RDKit descriptors 759 features	Total 2,276 data points; training 80%, testing 20%	below 100 °C	RF Regression: R^2 of 0.863 and the lowest RMSE of 11.106	Link
Paduszynski et al.[145]	953 distinct ILs	QSPR models, KNN, NB, SVM	CODESSA and DRAGON Descriptors	Total 1,179 data points	220 - 400	AARE 5.1%, RMSE 19K	no
Baskin et al.[55]	717 (379 cations, 81 anions)	QSPR, RFR	ISIDA Fragment Descriptors, Mold2 Descriptors, CDK23 Descriptors, Dragon7 Descriptors	Total 1,028 data points (10-fold cross-validation)		Q^2, RMSE, MAE: 0.6,39,27 K	no
Kilic et al.[146]	139 ILs	RF	rdkit descriptor, 20 descriptors calculated by PM3 semi-empirical	Training 75%, testing 25%		R^2 0.95, RMSE 0.34	no
Mashayekhi et al.[147]	510 ILs	DNN, VAE	molecular fingerprinting	Training 80%, testing 20%		R^2 0.9759, MSE 106.9762, MAE 6.0654	no
Liu et al.[148]	280 imidazolium-based ILs	NNs (multilayer perceptron network (MLP))	12 kinds of quantum chemical descriptors	Training 80%, testing 20%	below 283	R^2 0.75, MAE 25.03 K	no

Gao et al.[90]		classification problem: XGBoost	molecular fingerprints (morgan) and chemical descriptors	Total 4,742 data points (cations 1,893, anions 221)	below 298.15	accuracy 91.68%, precision 86.41%, recall 83.29%	no
Song et al.[64]	2,079 ILs	classification GNN model	RDKit	Total 2,079 data points	below 298.15	PRC-AUC 0.913, recall 0.9, precision 1	no
Torrecilla et al.[149]	97 imidazolium salts	ANN	fourteen input nodes	Total 87 data points	202 - 464.4	mean prediction error 1.30%, a regression coefficient 0.99 and a mean P-value 0.92	no
Liang et al.[150]		pSCNN model	1,631 molecular descriptors rdkit	Total 3,098 data points	177 - 592	MAE 24.36, RMSE 31.56 °C	Link
Venkatraman et al.[151]	2,212 ILs	Cubist, RF, GB regression	electronic, thermodynamic and geometrical descriptors	Total 2,212 data points; training 1,486, testing 726	178 - 632	Cubist: R^2 0.64	no
Li et al.[65]	1,362 cations, 137 anions, 2,186 ILs	Ionic Fragment Contribution (IFC) with GNNs	99 Ionic fragment molecular graph features	Total 2,186 data points	177 - 632	MAE 32.9296, R^2 0.73, RMSE 43.3328	Link

Table S6. Literature-Summary of Machine Learning Studies for the Prediction of Toxicity in Ionic Liquids

Reference	Families of ILs	ML Models	Descriptors	Size of Dataset	Toxicity	Performance	GitHub Link
Fatemi et al.[152]	227 ILs	genetic algorithm (GA), MLR, MLP NN		Total 227 data points; training 182, testing 45		R^2 0.98	no
Torrecilla et al.[153]	96 ILs	NN	Sigma profile	Training set of 105 compounds (96 ILs and 9 closely related salts)	−0.19 - 4.30 Log EC_{50} IPC-81	R^2 0.91	no
Wu et al.[154]	304 ILs	MLR	33 descriptors	Total 304 data points; training 228, testing 76	logEC_{50} (µM): −0.24 - 4.90	R^2 0.90	no
Abdullah et al.[155]	304 ILs	Ridge, LASSO, DT, RF, XGBoost, SVR	five molecular descriptors	Total 304 data points; training 212 70%, testing 92 30%		R^2 0.86, MAE 0.32, RMSE 0.43	no
Abdellatif et al.[156]	304 ILs (126 imidazolium, 61 pyridinium, 35 ammonium, 33 morpholinium, 28 pyrrolidinium, 17 piperidinium, 9 aminium, 5 phosphonium, 5 quinolinium, and 2 sulfonium)	dragonfly algorithm (DA), moth-flame optimization (MFO), and gray wolf optimizer (GWO) with SVM	13 descriptors AlvaDesc	Total 304 data points; training 244 (80%), testing 60 (20%)	(IPC-81) (log10(EC_{50})) (− 0.24 - 4.9)	DA-optimized SVM model: R^2 0.9742, RMSE 0.1787, MSE 0.0625	Link
Qin et al.[157]	24 binary mixtures	LR two mixture models (LCA and LIA)		Total 24 data points		R^2 0.99	no
Roy et al.[158]	49 ILs	MLR, PLS regression	computed lipophilicity, atom-type fragment, quantum	Total 49 data points		R^2 0.955, Q^2 0.917	no

			topological molecular similarity (QTMS) and extended topochemical atom (ETA) descriptors					
Grzonkowska et al.[159]	56 ILs	MLR	ChemSketch Descriptors, PM7 level using MOPAC 2012	Total 56 data points; training 42 (75%), validation 14 (25%)	log EC_{50}, 2.54 - 4.30	R^2 0.78, Q^2_{CV} 0.72, Q^2_{EXT} 0.75		no
He et al.[160]	169 and 101 ILs: MICs and MBCs	QSAR		Total 270 data points; training 169, testing 101		R^2 0.919, (SE) 0.341 (in log unit of mM) for pMIC, R^2 0.913 and SE of 0.282 for pMBC		no
Fan et al.[161]	155 ILs	DCNN model	218 feature descriptors Sigma profile and molecular fingerprint MACCS fingerprint	Total 155 data points; training 80%, testing 20%	log EC_{50} (−0.24 to 4.6)	R^2 0.965		no
Tabaaza et al.[162]	243 ILs	XGBoost, DT, RF, ETR, GBR	GC	Total 243 data points; training 206 (85%), testing 37 (15%)		R^2 0.79, RMSE 0.45, minimum MAE 0.32		no
Semenyuta et al.[163]		ASNN, Trans-CNN, RF	AlvaDesc descriptors	Dataset 1 – 75 data points, dataset 2 – 99 data points	dataset 1 (0.031 - 1,923 µM) dataset 2 (0.26 - 18,440 µM)	Q^2 0.77 - 0.82		no
Izadiyan et al.[164]	40 ILs	MLR and MLP	Structural descriptors		EC_{50} (−3.57 - 4.01)	MLP: R^2 0.976, standard error 0.254		no

Shan et al.[165]	237 ILs	RF, GBDT	digitized structural representations, rdkit, descriptors, fingerprints	Total 237 data points; training 80%, testing 20%	log EC_{50} (0.00-4.90) μM	R^2 0.9084 RMSE 0.3456	no
Torrecilla et al.[166]	153 ILs	MLR, RB, MLP, NN			log10 EC_{50} (IPC-81 and AchE,	mean MPE 3.3%, R^2 0.98	no
Zhao et al.[167]	100 ILs	MLR, SVM		Total 100 ILs; training 80%, testing 20%	EC_{50} values (−0.24 to 4.58)	SVM R^2 0.958, RMSE 0.234	no
Yan et al.[168]	157 ILs	MLR model	QSAR The topological index (TI)	Total 157 data points; training 80%, testing 20%		R^2 0.908 AAE 0.278	no
Cao et al.[169]	119 ILs	ELM, MLR, SVM	electrostatic potential surface area (SEP), sigma profile	Total 119 data points	IPC-81 (EC_{50} values) 0.17 - 4.58	R^2 0.937, AARD 4.73%, RMSE 0.189, Q^2_{ext} 0.940	no
Peng et al.[170]	127 ILs	MLR, MLP	Sigma profile, GC-COSMO	Total 127 data points; training 93, validation 15, testing 15		R^2 0.938, MSE 0.037	no
Sadaghiyanfam et al.[171]	355 chemical compounds	CNNs, XGBoost, SVR	molecular descriptors, chemical fingerprints, and deep learning-based embeddings generated through ChemBERTa	Total 355 ILs; training 70%, validation 15%, testing 15%	log EC_{50} values 0.64 - 3.59	CNN- R^2-0.865, RMSE 0.390, Pearson correlation coefficient 0.937	no
Salam et al.[172]	17 ILs (imidazolium, pyrrolidinium, and pyridinium)	MLR	Reactivity descriptors, EHOMO, ELUMO, Δ E, ω		Log10[EC_{50}] 1.63 - 3.86	R^2 0.999	no
Fan et al.[173]	145 ILs (imidazolium, pyridinium, pyrrolidinium,	RF, XGBoost,KNN, ANN	Sigma profile, 10 Descriptor	Total 1,590 data points	Log EC_{50} 0.36 - 5.34 μmol L^{-1}	R^2 RF 0.933, XGBoost 0.957	no

	piperidinium, phosphonium and others)						
Wu et al.[174]	160 ILs	RF and XGBoost	244 descriptors	Total 160 data points; training 70%, testing 30%	Log EC_{50} 0.2 - 3.0	XGBoost R^2 0.86, RMSE 0.14, MAE 0.11	no
Gao et al.[90]		GNN regression			$\log_{10}$ EC_{50} greater than 3.4 μM		no
Yan et al.[175]	153 ILs 62 cations and 20 anions	RF, KNN, XGBoost, ANN	3,150 Dragon descriptors, MOE descriptor	Training 80%, testing 20%			no
Fajar et al.[176]	186 ILs	RF	Sigma profile COSMO - RS	Total 150 data points		R^2 0.76	no
Zhang et al.[177]	103 ILs	MLP, RF, GBR, SVR	RDKit descriptors 209 descriptors, MACCS, and ECFP. QSAR models	Total 103 data points; training 80%, testing 20%	log EC_{50} −2.339 - 3.740	R^2 0.975, RMSE 0.222	no
Yan et al.[178]	173 ILs 75 imidazolium, 28 pyridinium, 18 pyrrolidinium, 20 ammonium, 4 phosphonium, 5 quinolinium, 13 piperidinium, 10 morpholinium cations, and 29 anions	MLR model	atom radius, atom electronegativity, etc.) and atom position in the hydrogen-suppressed molecule structure	Total 173 data points; training 138, testing 35	log EC_{50} IPC-81	R^2 0.938, AAE 0.226	no
Venkatraman et al.[48]	227 ILs	RF, Cubist, GBM	KRAKENX, Molecular descriptors (HOMO/LUMO energies,	Total 227 data points; calibration 157 (67%) validation 70 (33%)	IPC-81 (log10(EC_{50}) > 3.4 μM	Cubist: RMSE 0.40, R^2 0.86, MAE 0.30	no

			polarizabilities, superdelocalizabilities, charge partial surface areas (CPSA) and geometrical indices)					
Zhang et al.[179]	229 ILs	FE-SVM, FE-RF, FE-MLP, CNN-SVM, CNN-RF, CNN-MLP	208 descriptors	Total 266 data points	log EC_{50} [µM]		CNN-SVM model (0.947)	Link
Nu'aim et al.[180]	31 ILs	MLR	14 descriptors	Training 80%, testing 20%				no
Hodyna et al.[181]	long-chain pyridinium- and imidazolium-based ILs	ANN QSAR, k-NN	DRAGON descriptors	Total 1195 compounds; training 956, testing 239	IC_{50} ranging 0.0002 to 5,000 µM			no
Zhu et al.[182]	160 ILs	MLR ELM	σ-profiles	Total 160 data points; training 80%, testing 20%			ELM R^2 Test set 0.950, total data 0.964	no
Wang et al.[183]	355 ILs	FFNN, SVM	IL structural descriptors	Total 355 data points; training 284 (80%), testing 71 (20%)	IPC-81 (log EC_{50})		RMSE 0.3204, MAE 0.2628, R^2 0.9202	no
Sadaghiyanfam et al.[184]	355 ILs (pyridinium, ammonium, and morpholinium, amide, chloride, tetrafluoroborate, bromide, etc.	RF, CatBoost, SVR, Chemception with XGBoost meta-classifier	Morgan fingerprints (ECFP), 650 RDKit descriptors	Training 80%, testing 20%			RMSE 0.060812, R^2 0.996432, MAE 0.024410	Link
Feng et al.[185]	IPC-81 242 ILs, AchE 153 ILs, *E. coli* 125 ILs, *Vibrio fischeri* 165 ILs	CatBoost, RF, GB	207 features	Training 80%, testing 20%			R^2 0.845, 0.819, 0.859, 0.774	no

Table S7. Literature-Summary of Machine Learning Studies for the Prediction of Surface Tension in Ionic Liquids

Reference	Families of ILs	ML Models	Descriptors	Size of Dataset	Surface Tension, $mN \cdot m^{-1}$	Range of Pressure, kPa	Range of Temperature, K	Performance	GitHub Link
Obaid et al.[186]	69 ILs	GPR, SVR, DT	45 input features	Total 1,042 data points; training 80%, testing 20%	18.5 - 70.3		268.29 - 532.4	GPR: R^2 0.981	no
Golzar et al.[41]	MDEA, PZ, 12 ILs	ANN	w (weight fraction), M ($g \cdot mol^{-1}$), acentric factor), T (K), P (kPa)	Total 308 data points; training 216, validation 46, testing 46	42.638 - 65.556	0.177-3938.4	293.15 - 373.15	R^2 0.989, RMSE 0.593	no
Shojaeian et al.[187]	33 non-ionic + ILs binary mixture	ANN, ANFIS, ANFIS-ACO, ANFIS-DE, ANFIS-GA, and ANFIS-PSO		Total 1,537 data points	0.0189 - 0.0727 $N \cdot m^{-1}$		278.15 - 338.15	R^2 0.9998	no
Setiawan et al.[188]	31 binary ILs mixtures	ANN model with four optimization algorithms (PSO, GA, TLBO, and ICA)		Total 748 data points; training 75%, testing 25%			283.1 - 348.15	MSE & R^2 0.0000002 and 0.998	no
Yalcin et al.[189]	Protic ILs (PILs) with water and excess acid/base	MLR, Bayesian regularized ANNs	Alkyl chain length, hydroxyl groups on cation, anion type, nonstoichiometry, water presence	Total 207 data points; training 80%, testing 20%				MLR model: R^2 0.83, SEP 4.61	no
Bawazeer et al.[190]	69 ILs	RF, Decision tree (DT), Extremely randomized trees (ET)	chemical structure as inputs	Total 1,042 data points; training 80%, testing 20%	18.7 - 70.3		268.29 - 532.4	ET: R^2 0.979, MAPE, 2.05E-02, MAE 0.724 $mN \cdot m^{-1}$	no

Deng et al.[191]	59 organic solvents-ILs and four organic solvents-ILs - water	SVR, ANN, KNN, RFR, LightGBM CatBoost		Total 1,593 binary mixture data points and 216 ternary mixture data points			278.15-348.15	CatBoost: R^2 0.9946, RMSE 0.7565, MAE 0.3338	no
Zhu et al.[192]	289 ILs	RF, MLP	SMILES string and GC methods	Total 2,168 data points; training 1,734, testing 434	16.9 - 76.2	0.101 MPa	263.08 - 533.2	String-MLP: R^2 0.995, RMSE 0.686, AARD 0.71%	no
Paduszynski et al.[193]	570 ILs (196, 98 distinct cations and anions)	Stepwise MLR, feed-forward ANN, least-squares SVM (LSSVM)	GCs	Total 445 ILs and 3,841 data points; training validation 90%, testing 10%	15.5 - 100		263 - 533	GC + LSSVM: AARE (%) 5.3	no
Alshahrani et al.[194]	2 (1,3-dimethylimidazolium methylsulfate and 1-ethyl-3-methylimidazolium ethylsulfate)	Lasso, KNN, LR	45 input features	Total 1,042 data points (69 ILs)	18.5 - 70.3	0.101 MPa	268.29 - 532.4	Bagging LR, Bagging Lasso, and Bagging KNN: R^2 0.934, 0.915, 0.977	no
Mohan et al.[195]	360 ILs	Tree-based approaches, FFNN, XGBoost, gradient-boosting trees, SVM	COSMO-RS sigma profile	Total 2,524 data points; training 55%, testing 45%	16.9 - 76.2		263 - 533	XGBoost: R^2 0.994, RMSE 1.71 $mN \cdot m^{-1}$	Link
Golzar et al.[56]	five quaternary ammonium-based ILs ([N222(n)]Tf2N with n 5, 6, 8, 10, 12)	QSAR, ANN, GFA	GC	Training 70%, validation 15%, testing 15%	28.5 - 32.5 $mJ \cdot m^{-2}$		298 - 373	R^2 0.9962, RMSE 0.1929, ARD 0.0056, AAD 0.1632%	no

Baran et al.[51]	570 ILs	GNNs	Molecular graphs with structural information openbabel, rdkit	Total 6,000 data points; training 75&, validation 15%, testing 10%				R^2 0.99, RMSE 4.7, MARE 0.085	Link
Fu et al.[196]	138 ILs (imidazolium, pyridinium, pyrrolidinium, ammonium, phosphonium, sulfonium, piperidinium, morpholine)	ANN-GC model	Group contribution method combined with T and IL structure	Total 4,574 data points; training 3,562, testing 1,012			278.15 - 344.2	MAE 0.00082, R^2 0.9719	no
Soleimani et al.[197]	15 ILs ([C2mim][BF4], [C4mim][BF4], [C6mim][BF4])	SVM, GA-SVM, GA-LSSVM, CSA-LSSVM, GMDH-PNN and ANN	T, mole fraction, molecular properties	Total 748 data points			283.1 - 348.15	MRAE 1.06%	no
Baskin et al.[55]	407 ILs (157 cations, 77 anions)	XGBoost	chemical structures, fragment	Total 6,162 data points				Q^2 0.75, RMSE 0.0041, MAE 0.0027 $N\cdot m^{-1}$	no
Zarenezhad et al.[198]	alkanolamine-IL	SVM	T, ILs mass fraction, amines mass fraction, molecular weight of ILs, and molecular weight of amines	Total 327 data points; training 70%, testing 30%			293.15 - 343.15	AAD 0.44%, R^2 0.9935	no
Soleimani et al.[199]	48 different ILs	SGB	T, IL properties, and non-IL properties	Total 4,010 data points	0.0157 - 0.0727 $N\cdot m^{-1}$		278.15 - 348.15	MRAE 0.003989 R^2 0.99923	no

Mohan et al.[200]	360 ILs (119 cations and 63 anions)	MLR, Gradient Boosting Tree (GBT)	COSMO-RS molecular descriptors	Total 2,524 data points; training 1,388 (55%), testing 1,136 (45%)			263.08 - 533.2	R^2 0.96, RMSE 1.71 mN·m^{-1}	Link
Lemaoui et al.[60]	65 ILs families (579 ILs)	ANN	Sigma profile molecular features	Total 6,599 data points; training 5,405 (81.9%), testing 1,194 (18.1%)	15 - 85			R^2 0.931	no
Benmouloud et al.[201]	62 binary mixtures of IL and molecular solvent	ANN, LSSVM, SVM, Particle swarm optimization	COSMO-RS molecular descriptors sigma profiles, T, X_{IL}	Total 1,623 data points; training 70%, validation 15%, testing 15%	18.9 - 72.7			AARD 0.8466%, MSE 0.4952	no
Aboureheab et al.[202]	69 ILs	GENETIC-KNN, FOA-KNN, and PSO-KNN	T, chemical structure	Total 1,042 data points	18.5 - 70.3		268.29 - 532.4	R^2 GENETIC-KNN, FOA-KNN, and PSO-KNN 0.962, 0.941, and 0.827	no
Wang et al.[17]	406 ILs	KNN, SVR, RF, GBRT, XGBoost		Total 5,987 data points; training 80%, testing 20%	15.50 - 68.35		262.89 - 743.71	RF R^2 of 0.9560, RMSE 1.7737, MAE 0.9675	no
Cao et al.[77]	279 ILs	MLR, ridge regression (RR), ANN, SVR, RF, and XGB	65 features GC based	Total 2,768 data points			262.89 - 743.71	SVR R^2 0.952	no
Chen et al.[19]	133 different IL-IL binary mixtures	ANN, XGBoost, LightGBM	GC	Total 626 data points; training 549, testing 77			263.64-533.2	XGBoost R^2 0.9550, MAE 0.001170	no
Járvás et al.[203]	79 different ILs	ANN	COSMO-RS sigma profile	Total 880 data points; training	15.5 - 65.1		268 - 533	R^2 0.97, MAE 1 mN·m^{-1}	no

				616, validation 132, testing 132					
Lazzús et al.[204]	162 different ILs	GCM + ANN + GSA method	T, Mw, and 46 structural groups	Total 2,307 data points	0.015 - 0.062 $N \cdot m^{-1}$		263 - 533	MAPE 1.29%, R^2 0.991	no
Huang et al.[23]	28 binary mixtures	ANN model, Semi-empirical model		Total 573 data points; correlation dataset (28 data points) validation dataset (545 data points)			293.15 - 343.3	AARD 1.55%	no
Sheikhshoaei et al.[205]		gradient boosting neural networks (GrowNet) and deep neural networks (DNN), (CatBoost), (GPR)	T, IL Conc., Mw, Tc, Pc, ω	Total 1,020 data points; training 80%, testing 20%			288.15-318.15	CatBoost: AAPRE 0.19, RMSE 0.21, R^2 0.998	no
Lashkarbolooki et al.[206]	32 binary ILs	ANN model (multi-layer perception neural network (MLPNN))	T_m, Mw and mole fraction of ILs	Total 836 data points; testing 209				AARD%: 0.93, MSE: $6.67 \cdot 10^{-7}$, R^2 0.9950	no

References

(1) Barati-Harooni, A.; Najafi-Marghmaleki, A.; Mohammadi, A. H. Prediction of Heat Capacities of Ionic Liquids Using Chemical Structure Based Networks. *J. Mol. Liq.* **2017**, *227*, 324–332.

(2) Sattari, M.; Gharagheizi, F.; Ilani-Kashkouli, P.; Mohammadi, A. H.; Ramjugernath, D. Development of a Group Contribution Method for the Estimation of Heat Capacities of Ionic Liquids. *J. Therm. Anal. Calorim.* **2014**, *115* (2), 1863–1882.

(3) Lashkarbolooki, M.; Hezave, A. Z.; Ayatollahi, S. Artificial Neural Network as an Applicable Tool to Predict the Binary Heat Capacity of Mixtures Containing Ionic Liquids. *Fluid Phase Equilib.* **2012**, *324*, 102–107.

(4) Daneshfar, R.; Bemani, A.; Hadipoor, M.; Sharifpur, M.; Ali, H. M.; Mahariq I.; Abdeljawad, T. Estimating the Heat Capacity of Non-Newtonian Ionanofluid Systems Using ANN, ANFIS, and SGB Tree Algorithms. *Appl. Sci. (Basel)* **2020**, *10* (18), 6432.

(5) Esmaeili, A.; Hekmatmehr, H.; Moheisen Mohammad and Atashrouz, S.; Abedi, A.; Mohaddespour, A. Heat Capacity of Ionic Liquids: Toward Interpretable Chemical Structure-Based Machine Learning Approaches. *J. Chem. Inf. Model.* **2025**, *65* (8), 4010–4026.

(6) Kang, X.; Liu, X.; Li, J.; Zhao, Y.; Zhang, H. Heat Capacity Prediction of Ionic Liquids Based on Quantum Chemistry Descriptors. *Ind. Eng. Chem. Res.* **2018**, *57* (49), 16989–16994.

(7) Zhao, Y.; Zeng, S.; Huang, Y.; Afzal, R. M.; Zhang, X. Estimation of Heat Capacity of Ionic Liquids Using $S_{\sigma\text{-Profile}}$ Molecular Descriptors. *Ind. Eng. Chem. Res.* **2015**, *54* (51), 12987–12992.

(8) Shu, Y.; Du, L.; Lei, Y.; Hu, S.; Kuang, Y.; Fang, H.; Liu, X.; Chen, Y. Modeling Study on Heat Capacity, Viscosity, and Density of Ionic Liquid–Organic Solvent–Organic Solvent Ternary Mixtures via machine Learning. *Processes (Basel)* **2024**, *12* (7), 1420.

(9) Valderrama, J. O.; Cardona, L. F.; Campusano, R. A.; Rivas, F. An Analytical Predictive Model for the Heat Capacity of Imidazolium-Type Ionic Liquids Derived Directly from Artificial Neural Network Modeling. *Int. J. Thermophys.* **2023**, *44* (5), 68.

(10) Koutchoukali, O.; Haloui, I.; Koutchoukali, M. S. Heat Capacity Prediction of Complex Molecules by Mass Connectivity Index. *MATEC Web Conf.* **2013**, *3*, 1080.

(11) Keller, A. N.; Kelkar, P.; Baldea, M.; Stadtherr, M. A.; Brennecke, J. F. Thermophysical Property Prediction of Anion-Functionalized Ionic Liquids for CO_2 Capture. *J. Mol. Liq.* **2024**, *393*, 123634.

(12) Liu, X.; Gao, J.; Chen, Y.; Fu, Y.; Lei, Y. Machine Learning-Assisted Modeling Study on the Density and Heat Capacity of Ionic Liquid-Organic Solvent Binary Systems. *J. Mol. Liq.* **2023**, *390*, 122972.

(13) Fu, Y.; Liu, X.; Gao, J.; Lei, Y.; Chen, Y.; Zhang, X. Machine Learning Models for the Density and Heat Capacity of Ionic Liquid–Water Binary Mixtures. *Chin. J. Chem. Eng.* **2024**, *73*, 244–255.

(14) Valderrama, J. O.; Martinez, G.; Faúndez, C. A. Heat Capacity of Ionic Liquids Using Artificial Neural networks and the Concept of Mass Connectivity. *Int. J. Thermophys.* **2011**, *32* (5), 942–956.

(15) Sattari, M.; Gharagheizi, F.; Ilani-Kashkouli, P.; Mohammadi, A. H.; Ramjugernath, D. Estimation of the Heat Capacity of Ionic Liquids: A Quantitative–Property Relationship Approach. *Ind. Eng. Chem. Res.* **2013**, *52* (36), 13217–13221.

(16) Firoozirad, K.; Alagumalai, A.; Solomin Evgeny and Fangfang, F.; Ahn, H. S.; Mahian, O. Study on Thermophysical Properties of Alumina Nanoparticles Enhanced Ionic Liquids (NEILs): A Modeling Approach. *J. Mol. Liq.* **2021**, *332*, 115827.

(17) Wang, C.; Song, C.; Zhou, G.; Dai, Z.; Yang, Z. Developing Machine Learning Models for Predicting Multiple Physical Properties of Ionic Liquids through a Combined Constitution -Structure-Interaction Descriptor. *J. Chem. Eng. Data* **2024**, *69* (12), 4350–4361.

(18) Azadfar, R.; Shaabanzadeh, M.; Hashemi-Moghaddam, H.; Nafchi, A. M. Estimation of Heat Capacity of 143 Pure Ionic Liquids Using Artificial Neural Network. *Int. J. Thermophys.* **2022**, *43* (6), 81.

(19) Chen, Y.; Ma, S.; Lei, Y.; Liang, X.; Liu, X.; Kontogeorgis, G. M.; Gani, R. Ionic Liquid Binary Mixtures: Machine Learning-assisted Modeling, Solvent Tailoring, Process Design, and Optimization. *AIChE J.* **2024**, *70* (5), e18392.

(20) Mazari, S. A.; Siyal, A. R.; Solangi, N. H.; Ahmed, S.; Griffin, G.; Abro Rashid and Mubarak, N. M.; Ahmed, M.; Sabzoi, N. Prediction of Thermo-Physical Properties of 1-Butyl-3-Methylimidazolium Hexafluorophosphate for CO_2 Capture Using Machine Learning Models. *J. Mol. Liq.* **2021**, *327*, 114785.

(21) Parmar, N.; Bendová, M.; Wagner, Z. Heat Capacity Measurements by a Setaram MDSC3 Evo: Estimation of Deviation in the Measurement, Advanced Data Analysis by Mathematical Gnostics, and Prediction by the Artificial Neural Network. *J. Therm. Anal. Calorim.* **2025**, *150* (1), 313–325.

(22) Sahebalzamani, S.; Naghizadeh, A.; Mahmoudzadeh, A.; Ghader, S.; Hemmati-Sarapardeh, A. Modeling the Thermophysical Properties of Alumina Nanoparticles Ionic Liquids

(NEILs) Using Advanced Intelligent Techniques. *Int. J. Hydrogen Energy* **2025**, *158*, 150375.

(23) Huang, Y.; Zhang, X.; Zhao, Y.; Zeng, S.; Dong, H.; Zhang, S. New Models for Predicting Thermophysical Properties of Ionic Liquids Mixtures. *Phys. Chem. Chem. Phys.* **2015**, *17* (40), 26918–26929.

(24) Fatehi, M.-R.; Raeissi, S.; Mowla, D. An Artificial Neural Network to Calculate Pure Ionic Liquid Densities without the Need for Any Experimental Data. *J. Supercrit. Fluids* **2014**, *95*, 60–67.

(25) Cancilla, J. C.; Diaz-Rodríguez, P.; Matute, G.; Torrecilla, J. S. The Accurate Estimation of Physicochemical Properties of Ternary Mixtures Containing Ionic Liquids via Artificial Neural Networks. *Phys. Chem. Chem. Phys.* **2015**, *17* (6), 4533–4537.

(26) Díaz-Rodríguez, P.; Cancilla, J. C.; Matute, G.; Torrecilla, J. S. Determination of Physicochemical Properties of Pyridinium-Based Ionic Liquid Binary Mixtures with a Common Component through Neural Networks. *Ind. Eng. Chem. Res.* **2014**, *53* (2), 1015–1019.

(27) Najafi-Marghmaleki, A.; Khosravi-Nikou, M. R.; Barati-Harooni, A. A New Model for Prediction of Binary Mixture of Ionic Liquids + Water Density Using Artificial Neural Network. *J. Mol. Liq.* **2016**, *220*, 232–237.

(28) Huang, Y.; Zhao, Y.; Zeng, S.; Zhang, X.; Zhang, S. Density Prediction of Mixtures of Ionic Liquids and Molecular Solvents Using Two New Generalized Models. *Ind. Eng. Chem. Res.* **2014**, *53* (39), 15270–15277.

(29) Soman, D. P.; Kalaichelvi, P.; Radhakrishnan, T. K. Density Modelling and Apparent Molar Volume of Ionic Liquid-Butyl-3-Methylimidazolium Bromide in Water. *J. Braz. Soc. Mech. Sci. Eng.* **2022**, *44* (3), 79.

(30) Safarov, J.; Bussemer, C.; Aliyev Abilgani and Lafuente, C.; Hassel, E.; Abdulagatov, I. Effect of Temperature on Thermal (Density), Caloric (Heat Capacity), Acoustic (Speed of Sound) and Transport (Viscosity) Properties of 1-Octyl-3-Methylimidazolium Hexafluorophosphate at Atmospheric Pressure. *J. Chem. Thermodyn.* **2018**, *124*, 49–64.

(31) Marques, H.; Lopes, J. N. C.; Freitas, A. A. de; Shimizu, K.; Mendes, P. S. F. Robust Method for Property Prediction via Artificial Neural Networks: Incorporating Key Structural Features for Carbon Dioxide–Ionic Liquid Mixtures. *J. Phys. Chem. B* **2025**, *129* (1), 286–304.

(32) Bendimerad, R.; Petro, E. Discovery of Ionic Liquid Propellants for Electrospray Thrusters Using Molecular Descriptors and Machine Learning. *J. Elect. Propuls.* **2025**, *4* (1), 40.

(33) Lazzús, J. A. Estimation of Density as a Function of Temperature and Pressure for Imidazolium-Based Ionic Liquids Using a Multilayer Net with particle Swarm Optimization. *Int. J. Thermophys.* **2009**, *30* (3), 883–909.

(34) Lazzús, J. A. ρ–T–P Prediction for Ionic Liquids Using Neural Networks. *J. Taiwan Inst. Chem. Eng.* **2009**, *40* (2), 213–232.

(35) Kianfar, E.; Shirshahi, M.; Kianfar, F.; Kianfar, F. Simultaneous Prediction of the Density, Viscosity and Electrical Conductivity of Pyridinium-Based Hydrophobic Ionic Liquids Using Artificial Neural Network. *Silicon* **2018**, *10* (6), 2617–2625.

(36) Shojaee Ali, S.; Hezave Ali, Z.; Lashkarbolooki, M.; Shafipour Sadat, Z. Prediction of the Binary Density of the ILs+ Water Using Back-Propagated Feed Forward Artificial Neural Network. *Chem. Ind. Chem. Eng. Q.* **2014**, *20* (3), 325–338.

(37) Yousefi, F.; Amoozandeh, Z. Statistical Mechanics and Artificial Intelligence to Model the Thermodynamic Properties of Pure and Mixture of Ionic Liquids. *Chin. J. Chem. Eng.* **2016**, *24* (12), 1761–1771.

(38) Golzar, K.; Amjad-Iranagh, S.; Modarress, H. Prediction of Thermophysical Properties for Binary Mixtures of common Ionic Liquids with Water or Alcohol at Several Temperatures and Atmospheric Pressure by Means of Artificial Neural Network. *Ind. Eng. Chem. Res.* **2014**, *53* (17), 7247–7262.

(39) Barati-Harooni, A.; Najafi-Marghmaleki, A.; Mohammadi, A. H. A Reliable Radial Basis Function Neural Network Model (RBF-NN) for the Prediction of Densities of Ionic Liquids. *J. Mol. Liq.* **2017**, *231*, 462–473.

(40) Pan, L.; Wang, R.; Fang, H.; Yang Xiurong and Feng, X.; Wang, Y.; Qi, X.; Song Siwei and Zhang, Q. Accelerating Energetic Ionic Liquid Discovery: A Synergistic Fusion Strategy for Property Prediction. *J. Phys. Chem. B* **2025**, *129* (35), 8991–9004.

(41) Golzar, K.; Modarress, H.; Amjad-Iranagh, S. Evaluation of Density, Viscosity, Surface Tension and CO_2 Solubility for Single, Binary and Ternary Aqueous Solutions of MDEA, PZ and 12 Common ILs by Using Artificial Neural network (ANN) Technique. *Int. J. Greenhouse Gas Control* **2016**, *53*, 187–197.

(42) Taghizadehfard, M.; Hosseini, S. M.; Pierantozzi, M.; Alavianmehr, M. M. Predicting the Volumetric Properties of Pure and Mixture of amino Acid-Based Ionic Liquids. *J. Mol. Liq.* **2019**, *294*, 111604.

(43) Kovačević, S.; Podunavac-Kuzmanović, S.; Zec, N.; Papović, S.; Tot, A.; Dožić, S.; Vraneš Milan and Vastag, G.; Gadžurić, S. Computational Modeling of Ionic Liquids Density by Multivariate Chemometrics. *J. Mol. Liq.* **2016**, *214*, 276–282.

(44) Ma, S.; Lei, Y.; Chen, Y.; Liu, X.; Chen, Y. Coupling Machine Learning and Group Contribution Method to Predict Density and Viscosity of Ionic Liquid-Inorganic Solvent-Water Ternary Mixtures. *Chem. Eng. Commun.* **2025**, *212* (8), 1213–1232.

(45) Paduszyński, K. Extensive Databases and Group Contribution QSPRs of Ionic Liquid Properties. 1. Density. *Ind. Eng. Chem. Res.* **2019**, *58* (13), 5322–5338.

(46) Valderrama, J. O.; Reátegui, A.; Rojas, R. E. Density of Ionic Liquids Using Group Contribution and Artificial Neural Networks. *Ind. Eng. Chem. Res.* **2009**, *48* (6), 3254–3259.

(47) El-Harbawi, M.; Samir, B. B.; Babaa, M.-R.; Mutalib, M. I. A. A New QSPR Model for Predicting the Densities of Ionic Liquids. *Arab. J. Sci. Eng.* **2014**, *39* (9), 6767–6775.

(48) Venkatraman, V.; Evjen, S.; Lethesh, K. C.; Raj, J. J.; Knuutila, H. K.; Fiksdahl, A. Rapid, Comprehensive Screening of Ionic Liquids towards sustainable Applications. *Sustain. Energy Fuels* **2019**, *3* (10), 2798–2808.

(49) Lei, Y.; Ma, S.; Du, L.; Liu, X.; Wu, X.; Liang, X.; Kontogeorgis, G. M.; Chen, Y. Modeling Study on the Density and Viscosity of Ionic–Organic Solvent–Water Ternary Mixtures. *Ind. Eng. Chem. Res.* **2024**, *63* (23), 10444–10455.

(50) Umaña, J. E.; Cashen, R. K.; Zavala, V. M.; Gebbie, M. A. Uncovering Ion Transport Mechanisms in Ionic Liquids Using Data Science. *Digit. Discov.* **2025**, *4* (6), 1423–1436.

(51) Baran, K.; Kloskowski, A. Graph Neural Networks and Structural Information on Ionic Liquids: A Cheminformatics Study on Molecular Physicochemical Property Prediction. *J. Phys. Chem. B* **2023**, *127* (49), 10542–10555.

(52) Vakili-Nezhaad, G. R.; Mohammadzaheri, M.; Mohammadi, F.; Humaid, M. Density and Refractive Index of Binary Ionic Liquid mixtures with Common Cations/Anions, along with ANFIS Modelling. *Liquids (Basel)* **2022**, *2* (4), 432–444.

(53) Golparvar, A.; Bahreini, A.; Choubineh, A.; Wood, D. A. A Support Vector Machine Analysis to Predict Density of mixtures of Methanol and Six Ionic Liquids. *Monatsh. Chem.* **2018**, *149* (12), 2145–2152.

(54) Torrecilla, J. S.; Cancilla, J. C.; Aroca-Santos, R.; Pariente, E. S. Neural Networks to Estimate Physicochemical Properties of Choline Carboxylate Ionic Liquids. *ACS Sustain. Chem. Eng.* **2016**, *4* (2), 536–540.

(55) Baskin, I.; Epshtein, A.; Ein-Eli, Y. Benchmarking Machine Learning Methods for Modeling Physical Properties of Ionic Liquids. *J. Mol. Liq.* **2022**, *351*, 118616.

(56) Golzar, K.; Amjad-Iranagh, S.; Modarress, H. Prediction of Density, Surface Tension, and Viscosity of Quaternary Ammonium-Based Ionic Liquids ([N222(n)]Tf2N) by Means of Artificial Intelligence Techniques. *J. Dispers. Sci. Technol.* **2014**, *35* (12), 1809–1829.

(57) Lashkarbolooki, M.; Hezave, A. Z.; Babapoor, A. Correlation of Density for Binary Mixtures of Methanol+Ionic Liquids Using Back Propagation Artificial Neural Network. *Korean J. Chem. Eng.* **2013**, *30* (1), 213–220.

(58) Nordness, O.; Kelkar, P.; Lyu, Y.; Baldea, M.; Stadtherr, M. A.; Brennecke, J. F. Predicting Thermophysical Properties of Dialkylimidazolium Ionic Liquids from Sigma Profiles. *J. Mol. Liq.* **2021**, *334*, 116019.

(59) Makarov, D. M.; Fadeeva, Y. A.; Shmukler, L. E. Predictive Modeling of Physicochemical Properties and ionicity of Ionic Liquids for Virtual Screening of Novel Electrolytes. *J. Mol. Liq.* **2023**, *391*, 123323.

(60) Lemaoui, T.; Eid, T.; Darwish, A. S.; Arafat, H. A.; Banat, F.; AlNashef, I. Revolutionizing Inverse Design of Ionic Liquids through The Multi-Property Prediction of over 300,000 Novel Variants Using Ensemble Deep Learning. *Mater. Sci. Eng. R Rep.* **2024**, *159*, 100798.

(61) Cancilla, J. C.; Perez, A.; Wierzchoś, K.; Torrecilla, J. S. Neural Networks Applied to Determine the Thermophysical Properties of Amino Acid Based Ionic Liquids. *Phys. Chem. Chem. Phys.* **2016**, *18* (10), 7435–7441.

(62) Palomar, J.; Torrecilla, J. S.; Ferro, V. R.; Rodriguez, F. Development of an a Priori Ionic Liquid Design Tool. 1. Integration of a Novel COSMO-RS Molecular Descriptor on Neural Networks. *Ind. Eng. Chem. Res.* **2008**, *47* (13), 4523–4532.

(63) Rostami, A.; Baghban, A.; Shirazian, S. On the Evaluation of Density of Ionic Liquids: Towards a Comparative Study. *Chem. Eng. Res. Des.* **2019**, *147*, 648–663.

(64) Song, Y.; Guo, Y.; Chen, J.; Yuan, M.; Dong, K. Deep Learning Assisted High Throughput Screening of Ionic Liquid Electrolytes for NRR and CO2RR. *J. Environ. Chem. Eng.* **2023**, *11* (5), 110556.

(65) Li, K.; Song, C.; Jiang, H.; Zhu, Y.; Wang, M.; Zhang, X. An Explainable Ionic Liquid Property Model by Coupling Ionic Fragment Contribution with Graph Neural Network. *AIChE Journal* **2026**, *72* (2), e70113.

(66) Ma, R.; Zu, C.; Li, H.; Xin, L.; Liang, R.; Miao, C.; Wang, Y.; Zhu, Z.; Cui, P. Machine Learning Prediction of Density of Ionic Liquid–Organic Solvent Systems for Green Chemistry Applications: Ensemble Model, Interpretability, and Validation. *ACS Sustain. Chem. Eng.* **2025**, *13* (50), 21715–21728.

(67) Carrera, G. V. S. M.; da Ponte, M. Machine-learning Approaches to Tune Descriptors and Predict the viscosities of Ionic Liquids and Their Mixtures. *Chem. Methods* **2021**, *1* (5), 214–223.

(68) Carrera, G. V. S. M.; da Ponte, M.; Rebelo, L. P. N. Chemoinformatic Approaches to Predict the Viscosities of Ionic and Ionic Liquid-Containing Systems. *ChemPhysChem* **2019**, *20* (21), 2767–2773.

(69) Lashkarblooki, M.; Hezave, A. Z.; Al-Ajmi, A. M.; Ayatollahi, S. Viscosity Prediction of Ternary Mixtures Containing ILs Using Multi-Layer Perceptron Artificial Neural Network. *Fluid Phase Equilib.* **2012**, *326*, 15–20.

(70) Díaz-Rodríguez, P.; Cancilla, J. C.; Matute, G.; Torrecilla, J. S. Viscosity Estimation of Binary Mixtures of Ionic Liquids through a Multi-Layer Perceptron Model. *Journal of Industrial and Engineering Chemistry* **2015**, *21*, 1350–1353. https://doi.org/10.1016/j.jiec.2014.06.005.

(71) Moosavi, M.; Daneshvar, A.; Sedghamiz, E. Rheological Properties of [Bmim]PF6+methanol Mixtures at different Temperatures, Shear Rates and Compositions. *J. Mol. Liq.* **2015**, *209*, 693–705.

(72) Fatehi, M.-R.; Raeissi, S.; Mowla, D. Estimation of Viscosities of Pure Ionic Liquids Using an artificial Neural Network Based on Only Structural Characteristics. *J. Mol. Liq.* **2017**, *227*, 309–317.

(73) Moslehi, H.; Hosseini, S. M.; Pierantozzi, M.; Alavianmehr, M. M.; Haghighi, B. Modeling the Viscosity of Ionic Liquids Using Combined Friction Theory with Perturbed Hard-Chain Equation of State and Neural Network Approaches. *J. Mol. Liq.* **2023**, *383*, 122004.

(74) Paduszyński, K.; Domańska, U. Viscosity of Ionic Liquids: An Extensive Database and a New Group Contribution Model Based on a Feed-Forward Artificial Neural Network. *J. Chem. Inf. Model.* **2014**, *54* (5), 1311–1324.

(75) Beckner, W.; Mao, C. M.; Pfaendtner, J. Statistical Models Are Able to Predict Ionic Liquid viscosity across a Wide Range of Chemical Functionalities and Experimental Conditions. *Mol. Syst. Des. Eng.* **2018**, *3* (1), 253–263.

(76) Mousavi, S. P.; Atashrouz, S.; Nait Amar Menad and Hemmati-Sarapardeh, A.; Mohaddespour, A.; Mosavi, A. Viscosity of Ionic Liquids: Application of the Eyring's Theory and a Committee Machine Intelligent System. *Molecules* **2021**, *26* (1), 156.

(77) Cao, P.; Chen, J.; Chen, G.; Qi Zhiwen and Song, Z. A Critical Methodological Revisit on Group-Contribution Based Property Prediction of Ionic Liquids with Machine Learning. *Chem. Eng. Sci.* **2024**, *298* (120395), 120395.

(78) Fatehi, M.-R.; Raeissi, S.; Mowla, D. Estimation of Viscosity of Binary Mixtures of Ionic Liquids and solvents Using an Artificial Neural Network Based on the Structure Groups of the Ionic Liquid. *Fluid Phase Equilib.* **2014**, *364*, 88–94.

(79) Soman, D. P.; Karthika, S.; Kalaichelvi, P.; Radhakrishnan, T. K. Impact of Viscosity of Nanofluid and Ionic Liquid on Heat Transfer. *J. Mol. Liq.* **2019**, *291*, 111349.

(80) Chen, Y.; Peng, B.; Kontogeorgis, G. M.; Liang, X. Machine Learning for the Prediction of Viscosity of Ionic–Water Mixtures. *J. Mol. Liq.* **2022**, *350*, 118546.

(81) Huwaimel, B.; Alanazi, J.; Alanazi, M.; Alharby, T. N.; Alshammari, F. Computational Models Based on Machine Learning and Validation for Predicting Ionic Liquids Viscosity in Mixtures. *Sci. Rep.* **2024**, *14* (1), 31857.

(82) Dutt, N. V. K.; Ravikumar, Y. V. L.; Rani, K. Y. Representation of Ionic Liquid Viscosity-Temperature Data by Generalized Correlations and an Artificial Neural Network (ANN) Model. *Chem. Eng. Commun.* **2013**, *200* (12), 1600–1622.

(83) Valderrama, J. O.; Muñoz Jéssica Makarena and Rojas, R. E. Viscosity of Ionic Liquids Using the Concept of Mass and Artificial Neural Networks. *Korean J. Chem. Eng.* **2011**, *28* (6), 1451–1457.

(84) Zhao, Y.; Zhang, X.; Deng, L.; Zhang, S. Prediction of Viscosity of Imidazolium-Based Ionic Liquids Using MLR and SVM Algorithms. *Comput. Chem. Eng.* **2016**, *92*, 37–42.

(85) Zimmermann, A. S.; Mattedi, S. Feedforward and Cascade Forward Networks for Viscosity Prediction for Binary Mixtures of Ammonium-Based Ionic liquids and Water. *Fluid Phase Equilib.* **2022**, *556*, 113416.

(86) Fan, J.; Dai, Z.; Cao, J.; Mu, L.; Ji, X.; Lu, X. Hybrid Data-Driven and Physics-Based Modeling for Viscosity Prediction of Ionic Liquids. *Green Energy Environ.* **2024**, *9* (12), 1878–1890.

(87) Ding, Y.; Chen, M.; Guo, C.; Zhang, P.; Wang, J. Molecular Fingerprint-Based Machine Learning Assisted QSAR Development for Prediction of Ionic Liquid Properties. *J. Mol. Liq.* **2021**, *326*, 115212.

(88) Boualem, A. D.; Argoub, K.; Benkouider, A. M.; Yahiaoui, A.; Toubal, K. Viscosity Prediction of Ionic Liquids Using MLR and SVM. *J. Mol. Liq.* **2022**, *368*, 120610.

(89) Huang, M.; Deng, J.; Jia, G. Predicting Viscosity of Ionic Liquids - Water Mixtures by Bridging UNIFAC Modeling with Interpretable Machine Learning. *J. Mol. Liq.* **2023**, *383*, 122095.

(90) Gao, J.; Guo, Y.; Yu, Y.; Wang Zhenlei and Dong, K. Rationally Design the Ionic Liquid-Based Absorbents for CO_2 Absorption Using Machine Learning. *Sep. Purif. Technol.* **2025**, *364*, 132613.

(91) Zhong, S.; Chen, Y.; Li, J.; Igou, T.; Xiong, A.; Guan, J.; Dai, Z.; Cai Xuanying and Qu, X.; Chen, Y. Screening Environmentally Benign Ionic Liquids for CO_2 Using

Representation Uncertainty-Based Machine. *Environ. Sci. Technol. Lett.* **2024**, *11* (11), 1193–1199.

(92) Paduszyński, K. Extensive Databases and Group Contribution QSPRs of Ionic Liquids Properties. 2. Viscosity. *Ind. Eng. Chem. Res.* **2019**, *58* (36), 17049–17066.

(93) Mohan, M.; Jetti, K. D.; Guggilam, S.; Smith, M. D.; Kidder, M. K.; Smith, J. C. High-Throughput Screening and Accurate Prediction of Ionic Liquid Viscosities Using Interpretable Machine Learning. *ACS Sustain. Chem. Eng.* **2024**, *12* (18), 7040–7054.

(94) Billard, I.; Marcou, G.; Ouadi, A.; Varnek, A. In Silico Design of New Ionic Liquids Based on Quantitative Structure-Property Relationship Models of Ionic Liquid Viscosity. *J. Phys. Chem. B* **2011**, *115* (1), 93–98.

(95) Kiani, S.; Hadavimoghaddam, F.; Atashrouz Saeid and Nedeljkovic, D.; Hemmati-Sarapardeh Abdolhossein and Mohaddespour, A. Modeling of Ionic Liquids Viscosity via Advanced White-Box Learning. *Sci. Rep.* **2024**, *14* (1), 8666.

(96) Sheikhshoaei, A. H.; Sanati, A. New Insight into Viscosity Prediction of Imidazolium-Based Ionic Liquids and Their Mixtures with Machine Learning Models. *Sci. Rep.* **2025**, *15* (1), 22672.

(97) Zhao, Y.; Huang, Y.; Zhang, X.; Zhang, S. A Quantitative Prediction of the Viscosity of Ionic Liquids $S_{\sigma\text{-Profile}}$ Molecular Descriptors. *Phys. Chem. Chem. Phys.* **2015**, *17* (5), 3761–3767.

(98) Duong, D. V.; Tran, H.-V.; Pathirannahalage, S. K.; Brown, S. J.; Hassett, M.; Yalcin, D.; Meftahi, N.; Christofferson, A. J.; Greaves, T. L.; Le, T. C. Machine Learning Investigation of Viscosity and Ionic Conductivity of Protic Ionic Liquids in Water Mixtures. *J. Chem. Phys.* **2022**, *156* (15), 154503.

(99) Han, Z. Theoretical and Computational Investigations on Estimation of Viscosity of Ionic Liquids for Green Adsorbent: Effect of Temperature and Composition. *Case Stud. Therm. Eng.* **2025**, *65*, 105703.

(100) Valeh-e-Sheyda, P.; Faridi Masouleh, M.; Zarei-Kia, P. Prediction of CO_2 Solubility in Pyridinium-Based Ionic Liquids New Descriptor-Based Chemoinformatics Models. *Fluid Phase Equilib.* **2021**, *546*, 113136.

(101) Laakso, J.-P.; Gorji, A. E.; Uusi-Kyyny, P.; Alopaeus, V. Machine Learning Modeling of the CO_2 Solubility in Ionic by Using σ-Profile Descriptors. *Chem. Eng. Sci.* **2025**, 307, 121226.

(102) Li, J.; Qi, X.; Zhang, Z.; Wang Yingying and Dang, L.; Li, Y.; Wang, L.; Zhang, J. Screening of Ionic Liquids for Efficient CO_2 Cycloaddition Catalysis under Mild Condition:

A Combined Machine and DFT Approach. *ACS Sustain. Chem. Eng.* **2024**, *12* (48), 17512–17522.

(103) Sheikhshoaei, A. H.; Sanati, A.; Khoshsima, A. Deep Learning Models to Predict CO_2 Solubility in Imidazolium-Based Ionic Liquids. *Sci. Rep.* **2025**, *15* (1), 26445.

(104) Liu, T.; Fan, D.; Chen, Y.; Dai, Y.; Jiao, Y.; Cui, P.; Wang, Y.; Zhu, Z. Prediction of CO_2 Solubility in Ionic Liquids via Convolutional Autoencoder Based on Molecular Structure Encoding. *AIChE J.* **2023**, *69* (10), e18182.

(105) Deng, T.; Liu, F.-H.; Jia, G.-Z. Prediction Carbon Dioxide Solubility in Ionic Liquids Based on Deep Learning. *Mol. Phys.* **2020**, *118* (6), e1652367.

(106) Song, Z.; Shi, H.; Zhang, X.; Zhou, T. Prediction of CO_2 Solubility in Ionic Liquids Using Machine Learning Methods. *Chem. Eng. Sci.* **2020**, *223* (115752), 115752.

(107) Bastami, Z.; Sobati, M. A.; Amereh, M. Predicting CO_2 Solubility in Diverse Ionic Liquids: A Data-Driven Approach Using Machine Learning Algorithms. *Energy Fuels* **2025**, *39* (23), 11256–11278.

(108) Rozhenko, A.; Hadavimoghaddam, F.; Valeh-E-Sheyda, P.; Tamtaji, M.; Abdi, J. Application of Machine Learning Approaches for Estimating Carbon Absorption Capacity of a Variety of Blended Imidazolium-Based Ionic Liquids. *J. Mol. Graph. Model.* **2025**, *139*, 109060.

(109) Torrecilla, J. S.; Palomar, J.; Garcia, J.; Rojo, E.; Rodriguez, F. Modelling of Carbon Dioxide Solubility in Ionic Liquids at Sub and Supercritical Conditions by Neural Networks and Mathematical Regressions. *Chemometr. Intell. Lab. Syst.* **2008**, *93* (2), 149–159.

(110) Zhao, F.; Liu, S.; Liu, Q.; Cheng Yongqiang and Song, M.; Lv, Q.; Jiang, B.; Li Guoxuan and Cui, P.; Lei, Z. Computational Molecular Engineering of Ionic Liquids for CO_2 Drying: Design Strategies and Molecular Mechanisms. *AIChE J.* **2025**, *71(10)*, e70014.

(111) Valeh-e-Sheyda, P.; Heidarian, P.; Rezvani, A. A Novel Molecular Structure -Based Model for Prediction of CO_2 Equilibrium Absorption in Blended Imidazolium-Based Ionic. *J. Mol. Liq.* **2022**, *360*, 119420.

(112) Benmouloud, W.; Euldji, I.; Si-Moussa, C.; Benkortbi, O. Quantitative Structure-property Relationship Techniques for Predicting Carbon Dioxide Solubility in Ionic Liquids Using Machine Learning Methods. *Int. J. Quantum Chem.* **2024**, *124* (15), e27450.

(113) Yang, A.; Sun, S.; Mi, H.; Wang, W.; Liu, J.; Kong, Z. Y. Interpretable Feedforward Neural Network and XGBoost-Based Algorithms to Predict CO_2 Solubility in Ionic Liquids. *Ind. Eng. Chem. Res.* **2024**, *63* (18), 8293–8305.

(114) Qin, H.; Wang, K.; Ma, X.; Li, F.; Liu, Y.; Ji, X. Predicting the Solubility of CO_2 and N_2 in Ionic Liquids Based on COSMO-RS and Machine Learning. *Front. Chem.* **2024**, *12*, 1480468.

(115) Tian, Y.; Wang, X.; Liu, Y.; Hu, W. Prediction of CO_2 and N_2 Solubility in Ionic Liquids Using a combination of Ionic Fragments Contribution and Machine Learning Methods. *J. Mol. Liq.* **2023**, *383* (122066), 122066.

(116) Jun, Z.; Dai, P.; Kong, Z. Y.; Yang, A.; Shen, W.; Wang, Q. Message Passing Neural Network-Based Contribution Analysis Towards CO_2 Solubility Prediction in Ionic Liquids. *Sep. Purif. Technol.* **2025**, *364*, 132361.

(117) Aghaie, M.; Zendehboudi, S. Estimation of CO_2 Solubility in Ionic Liquids Using Connectionist Tools Based on Thermodynamic and Structural Characteristics. *Fuel* **2020**, *279*, 117984.

(118) Tatar, A.; Naseri, S.; Bahadori, M.; Hezave, A. Z.; Kashiwao, T.; Bahadori, A.; Darvish, H. Prediction of Carbon Dioxide Solubility in Ionic Liquids Using MLP and Radial Basis Function (RBF) Neural Networks. *J. Taiwan Inst. Chem. Eng.* **2016**, *60*, 151–164.

(119) Rahimi, A.; Bahmanzadegan, F.; Ghaemi, A. Analysis of CO_2 Solubility in Ionic Liquids as Promising Absorbents Using Response Surface Methodology and Machine Learning. *J. CO2 Util.* **2025**, *93* (103043), 103043.

(120) Agwu, O. E.; Alatefi, S.; Alkouh, A.; Abdel Azim, R.; Wee, S. C. Carbon Capture Using Ionic Liquids: An Explicit Data Driven for Carbon (IV) Oxide Solubility Estimation. *J. Clean. Prod.* **2024**, *472*, 143508.

(121) Geng, R.; Deng, W.; Hu, Z.; Wang, J.; Zhao, Y.; Zhou, B.; Tian, G. Machine Learning with New Functional Structure Descriptors for Design and Screening of Ionic Liquids in CO_2 Efficient Capture. *Phys. Chem. Chem. Phys.* **2025**, *27* (27), 14482–14491.

(122) Liu, Z.; Bian, X.-Q.; Duan, S.; Wang, L.; Fahim, R. I. Estimating CO_2 Solubility in Ionic Liquids by Using Machine Methods. *J. Mol. Liq.* **2023**, *391* (123308), 123308.

(123) Yang, A.; Sun, S.; Su, Y.; Kong, Z. Y.; Ren, J.; Shen, W. Insight to the Prediction of CO_2 Solubility in Ionic Liquids Based on the Interpretable Machine Learning Model. *Chem. Eng. Sci.* **2024**, *297*, 120266.

(124) Mesbah, M.; Shahsavari, S.; Soroush Ebrahim and Rahaei, N.; Rezakazemi, M. Accurate Prediction of Miscibility of CO_2 and Supercritical in Ionic Liquids Using Machine Learning. *J. CO2 Util.* **2018**, *25*, 99–107.

(125) Ali, M.; Sarwar, T.; Mubarak, N. M.; Karri, R. R.; Ghalib, L.; Bibi, A.; Mazari, S. A. Prediction of CO_2 Solubility in Ionic Liquids for CO_2 Capture Using Deep Learning Models. *Sci. Rep.* **2024**, *14* (1), 14730.

(126) Moosanezhad-Kermani, H.; Rezaei, F.; Hemmati-Sarapardeh, A.; Band, S. S.; Mosavi, A. Modeling of Carbon Dioxide Solubility in Ionic Liquids Based on Group Method of Data Handling. *Eng. Appl. Comput. Fluid Mech.* **2021**, *15* (1), 23–42.

(127) Baghban, A.; Mohammadi, A. H.; Taleghani, M. S. Rigorous Modeling of CO_2 Equilibrium Absorption in Ionic Liquids. *Int. J. Greenhouse Gas Control* **2017**, *58*, 19–41.

(128) Balchandani, S. C.; Dey, A. Prediction of CO_2 Solubility in Potential Blends of Ionic Liquids with Alkanolamines Using Statistical Non-Rigorous and ANN Based Modeling: A Comprehensive Simulation Study for Post CO_2 Capture. *Int. Commun. Heat Mass Transf.* **2022**, *132*, 105866.

(129) Chen, X.; Chen, G.; Xie, K.; Cheng, J.; Chen, J.; Song, Z.; Qi, Z. Exploring the Chemical Space of Ionic Liquids for CO_2 Dissolution through Generative Machine Learning Models. *Green Chemical Engineering* **2025**, *6* (3), 335–343.

(130) Fierro, E. N.; Faúndez, C. A.; Muñoz, A. S.; Cerda, P. I. Application of a Single Multilayer Perceptron Model to predict the Solubility of CO_2 in Different Ionic Liquids for Gas Removal Processes. *Processes (Basel)* **2022**, *10* (9), 1686.

(131) Davoodi, S.; Thanh, H. V.; Wood, D. A.; Mehrad, M.; Hajsaeedi, M. R.; Rukavishnikov, V. S. Combined Deep-Learning Optimization Predictive Models for Determining Carbon Dioxide Solubility in Ionic Liquids. *J. Ind. Inf. Integr.* **2024**, *41*, 100662.

(132) Mirarab, M.; Sharifi, M.; Behzadi, B.; Ghayyem, M. A. Intelligent Prediction of CO_2 Capture in Propyl Amine Methyl Imidazole Alanine Ionic Liquid: An Artificial Neural Network Model. *Sep. Sci. Technol.* **2015**, *50* (1), 26–37.

(133) Ghiasi, M. M.; Mohammadi, A. H. Application of Decision Tree Learning in Modelling CO_2 Equilibrium Absorption in Ionic Liquids. *J. Mol. Liq.* **2017**, *242*, 594–605.

(134) Venkatraman, V.; Alsberg, B. K. Predicting CO_2 Capture of Ionic Liquids Using Machine Learning. *J. CO2 Util.* **2017**, *21*, 162–168.

(135) Hamzehie, M. E.; Najibi, H. Prediction of Acid Gas Solubility in Amine, Ionic Liquid and amino Acid Salt Solutions Using Artificial Neural Network and Evaluating with New Experimental Measurements. *J. Nat. Gas Sci. Eng.* **2016**, *29*, 252-263.

(136) Hamzehie, M. E.; Fattahi, M.; Najibi, H.; der Bruggen, B.; Mazinani, S. Application of Artificial Neural Networks for Estimation of solubility of Acid Gases (H_2S and CO_2) in 32 Commonly Ionic Liquid and Amine Solutions. *J. Nat. Gas Sci. Eng.* **2015**, *24*, 106–114.

(137) Bini, R.; Chiappe, C.; Duce, C.; Micheli, A.; Solaro, R.; Starita, A.; Tiné, M. R. Ionic Liquids: Prediction of Their Melting Points by a Recursive Network Model. *Green Chem.* **2008**, *10* (3), 306.

(138) Wang, K.; Xu, H.; Yang, C.; Qiu, T. Machine Learning-Based Ionic Liquids Design and Process Simulation for CO_2 Separation from Flue Gas. *Green Energy Environ.* **2021**, *6* (3), 432–443.

(139) Feng, H.; Qin, L.; Zhang, B.; Zhou, J. Prediction and Interpretability of Melting Points of Ionic Liquids Using Graph Neural Networks. *ACS Omega* **2024**, *9* (14), 16016–16025.

(140) Varnek, A.; Kireeva, N.; Tetko, I. V; Baskin, I. I.; Solov'ev, V. P. Exhaustive QSPR Studies of a Large Diverse Set of Ionic Liquids: How Accurately Can We Predict Melting Points? *J. Chem. Inf. Model.* **2007**, *47* (3), 1111–1122.

(141) Fatemi, M. H.; Izadian, P. In Silico Prediction of Melting Points of Ionic Liquids by Using Multilayer Perceptron Neural Networks. *J. Theor. Comput. Chem.* **2012**, *11* (01), 127–141.

(142) Lazzús, J. A. A Group Contribution Method for Predicting the Freezing Point of Ionic Liquids. *Period. Polytech. Chem. Eng.* **2016**, *60* (4), 273–281.

(143) Lazzús, J. A. A Group Contribution Method to Predict the Melting Point of Ionic Liquids. *Fluid Phase Equilib.* **2012**, *313*, 1–6.

(144) Qu, M.; Sharma, G.; Wada, N.; Ikebata, H.; Matsunami, S.; Takahashi, K. Machine Learning-Driven Generation and Screening of Potential Ionic Liquids for Cellulose Dissolution. *J. Cheminform.* **2025**, *17* (1), 78.

(145) Paduszyński, K.; Kł ebowski, K.; Królikowska, M. Predicting Melting Point of Ionic Liquids Using QSPR Approach: Literature Review and New Models. *J. Mol. Liq.* **2021**, *344*, 117631.

(146) Kilic, A.; Uzun, A.; Yildirim, R.; Eroglu, D. Ionic Liquid Electrolytes for Metal-Air Batteries: High-Throughput Screening and Machine Learning Modeling. *Electrochim. Acta* **2025**, *524*, 145997.

(147) Mashayekhi, A. Accelerating the Discovery of Ionic Liquids for Zinc Electrochemical Applications. *Comput. Theor. Chem.* **2025**, *1251*, 115340.

(148) Liu, X.; Yin, J.; Zhang, X.; Qiu, W.; Jiang, W.; Zhang, M.; Zhu, L.; Li, H.; Li, H. Rapid and Accurate Prediction of the Melting Point for Imidazolium-Based Ionic Liquids by Artificial Neural Network. *Chemistry (Basel)* **2024**, *6* (6), 1552–1571.

(149) Torrecilla, J. S.; Rodriguez, F.; Bravo, J. L.; Rothenberg, G.; Seddon, K. R.; López-Martin, I. Optimising an Artificial Neural Network for Predicting the Melting Point of Ionic Liquids. *Phys. Chem. Chem. Phys.* **2008**, *10* (38), 5826–5831.

(150) Liang, T.; Liu, W.; Tan, K.; Wu, A.; Lu, X. Advancing Ionic Liquid Research with pSCNN: A Novel approach for Accurate Normal Melting Temperature Predictions. *ACS Omega* **2024**, *9* (29), 31694–31702.

(151) Venkatraman, V.; Evjen, S.; Knuutila Hanna K and Fiksdahl, A.; Alsberg, B. K. Predicting Ionic Liquid Melting Points Using Machine Learning. *J. Mol. Liq.* **2018**, *264*, 318–326.

(152) Fatemi, M. H.; Izadiyan, P. Cytotoxicity Estimation of Ionic Liquids Based on Their Effective Structural Features. *Chemosphere* **2011**, *84* (5), 553–563.

(153) Torrecilla, J. S.; Palomar, J.; Lemus, J.; Rodriguez, F. A Quantum-Chemical-Based Guide to Analyze/Quantify the Cytotoxicity of Ionic Liquids. *Green Chem.* **2010**, *12* (1), 123–134.

(154) Wu, T.; Li, W.; Chen, M.; Zhou, Y.; Zhang, Q. Estimation of Ionic Liquids Toxicity Against Leukemia Rat Cell IPC-81 Based on the Empirical-like Models Using Intuitive and Explainable Fingerprint Descriptors. *Mol. Inform.* **2020**, *39* (10), e2000102.

(155) Abdullah, N. H.; Zaini, D.; Lal, B. Prediction of Ionic Liquids Toxicity Using Machine Learning for Application to Gas Hydrate. *Process Saf. Prog.* **2024**, *43* (S1), S199–S212.

(156) Abdellatif, H.; Laidi, M.; Si-moussa, C.; Amrane, A.; Euldji, I.; Benmouloud, W. Contributions to the Development of Prediction Models for the Toxicity of Ionic Liquids. *Struct. Chem.* **2025**, 36, 865-886.

(157) Qin, L.-T.; Wu, J.; Mo, L.-Y.; Zeng, H.-H.; Liang, Y.-P. Linear Regression Model for Predicting Interactive Mixture Toxicity of Pesticide and Ionic Liquid. *Environ. Sci. Pollut. Res. Int.* **2015**, *22* (16), 12759–12768.

(158) Roy, K.; Das, R. N.; Popelier, P. L. A. Quantitative Structure-Activity Relationship for Toxicity of Ionic Liquids to *Daphnia Magna*: Aromaticity vs. Lipophilicity. *Chemosphere* **2014**, *112*, 120–127.

(159) Grzonkowska, M.; Sosnowska, A.; Barycki, M.; Rybinska, A.; Puzyn, T. How the Structure of Ionic Liquid Affects Its Toxicity to *Vibrio fischeri*? *Chemosphere* **2016**, *159*, 199–207.

(160) He, W.; Yan, F.; Jia, Q.; Xia, S.; Wang, Q. QSAR Models for Describing the Toxicological Effects of ILs Against Staphylococcus Aureus Based on Norm Indexes. *Chemosphere* **2018**, *195*, 831–838.

(161) Fan, D.; Xue, K.; Liu, Y.; Zhu Wenguang and Chen, Y.; Cui, P.; Sun, S.; Qi, J.; Zhu, Z.; Wang, Y. Modeling the Toxicity of Ionic Liquids Based on Deep Learning. *Comput. Chem. Eng.* **2023**, *176* (108293), 108293.

(162) Tabaaza, G. A.; Tackie-Otoo, B. N.; Zaini, D. B.; Otchere, D. A.; Lal, B. Application of Machine Learning Models to Predict Cytotoxicity of Ionic Liquids Using VolSurf Principal Properties. *Comput. Toxicol.* **2023**, *26*, 100266.

(163) Semenyuta, I.; Kovalishyn, V.; Hodyna, D.; Startseva, Y.; Rogalsky, S.; Metelytsia, L. New QSTR Models to Evaluation of Imidazolium- and Pyridinium-Contained Ionic Liquids Toxicity. *Comput. Toxicol.* **2024**, *30*, 100309.

(164) Izadiyan, P.; Fatemi, M. H.; Izadiyan, M. Elicitation of the Most Important Structural Properties of Ionic Liquids Affecting Ecotoxicity in Limnic Green Algae; a QSAR Approach. *Ecotoxicol. Environ. Saf.* **2013**, *87*, 42–48.

(165) Shan, R.; Zhang, R.; Gao, Y.; Wang, W.; Zhu, W.; Xin, L.; Liu, T.; Wang, Y.; Cui, P. Evaluating Ionic Liquid Toxicity with Machine Learning and Structural Similarity Methods. *Green Chemical Engineering* **2025,** *6(2),* 249-262.

(166) Torrecilla, J. S.; Garcia, J.; Rojo, E.; Rodriguez, F. Estimation of Toxicity of Ionic Liquids in Leukemia Rat Cell Line and Acetylcholinesterase Enzyme by Principal Component, Neural Networks and Multiple Lineal Regressions. *J. Hazard. Mater.* **2009**, *164* (1), 182–194.

(167) Zhao, Y.; Zhao, J.; Huang, Y.; Zhou Qing and Zhang, X.; Zhang, S. Toxicity of Ionic Liquids: Database and Prediction via Quantitative Structure-Activity Relationship Method. *J. Hazard. Mater.* **2014**, *278*, 320–329.

(168) Yan, F.; Shang, Q.; Xia, S.; Wang Qiang and Ma, P. Topological Study on the Toxicity of Ionic Liquids on Vibrio fischeri by the Quantitative Structure-Activity Relationship Method. *J. Hazard. Mater.* **2015**, *286*, 410–415.

(169) Cao, L.; Zhu, P.; Zhao, Y.; Zhao, J. Using Machine Learning and Quantum Chemistry Descriptors to predict the Toxicity of Ionic Liquids. *J. Hazard. Mater.* **2018**, *352*, 17–26.

(170) Peng, D.; Picchioni, F. Prediction of Toxicity of Ionic Liquids Based on GC-COSMO Method. *J. Hazard. Mater.* **2020**, *398*, 122964.

(171) Sadaghiyanfam, S.; Kamberaj, H.; Isler, Y. Leveraging ChemBERTa and Machine Learning for Accurate Toxicity Prediction of Ionic Liquids. *J. Taiwan Inst. Chem. Eng.* **2025**, *171*, 106030.

(172) Salam, M. A.; Abdullah, B.; Ramli, A.; Mujtaba, I. M. Structural Feature Based Computational Approach of Toxicity of Ionic Liquids: Cationic and Anionic Effects on Ionic Liquids Toxicity. *J. Mol. Liq.* **2016**, *224*, 393–400.

(173) Fan, D.; Xue, K.; Zhang, R.; Zhu, W.; Zhang, H.; Qi, J.; Zhu, Z.; Wang, Y.; Cui, P. Application of Interpretable Machine Learning Models to Improve the Prediction Performance of Ionic Liquids Toxicity. *Sci. Total Environ.* **2024**, *908*, 168168.

(174) Wu, X.; Gong, J.; Ren, S.; Tan, F.; Wang, Y.; Zhao, H. A Machine Learning-Based QSAR Model Reveals Important Molecular Features for Understanding the Potential Inhibition Mechanism of Ionic Liquids to Acetylcholinesterase. *Sci. Total Environ.* **2024**, *915*, 169974.

(175) Yan, J.; Yan, X.; Hu, S.; Zhu, H.; Yan, B. Comprehensive Interrogation on Acetylcholinesterase Inhibition by Ionic Liquids Using Machine Learning and Molecular Modeling. *Environ. Sci. Technol.* **2021**, *55* (21), 14720–14731.

(176) Fajar, A. T. N.; Hartono, A. D.; Moshikur Rahman Md.; Goto, M. Ionic Liquids Curated by Machine Learning for Metal Extraction. *ACS Sustain. Chem. Eng.* **2022**, *10* (38), 12698–12705.

(177) Zhang, R.; Wang, Y.; Zhu, W.; Xin, L.; Qi, J.; Wang, Y.; Zhu, Z.; Cui, P. Insight into the Mechanism of Machine Learning Models for predicting Ionic Liquids Toxicity Based on Molecular Structure. *ACS Sustain. Chem. Eng.* **2024**, *12* (49), 17749–17760.

(178) Yan, F.; Xia, S.; Wang, Q.; Ma, P. Predicting the Toxicity of Ionic Liquids in Leukemia Rat Cell Line by the Quantitative Structure–Activity Relationship Method Topological Indexes. *Ind. Eng. Chem. Res.* **2012**, *51* (43), 13897–13901.

(179) Zhang, R.; Chen, Y.; Fan, D.; Liu, T.; Ma, Z.; Dai Y and Wang, Y.; Zhu, Z. Modelling Enzyme Inhibition Toxicity of Ionic Liquid from Molecular Structure via Convolutional Neural Network Model. *SAR QSAR Environ. Res.* **2023**, *34* (10), 789–803.

(180) Nu'aim, M. N.; Bustam, M. A. Toxicity Prediction of Ionic Liquids Based on Daphnia Magna by using Density Functional Theory. *IOP Conf. Ser. Mater. Sci. Eng.* **2018**, *344*, 12017.

(181) Hodyna, D.; Kovalishyn, V.; Semenyuta, I.; Rogalsky, S.; Trokhimenko, O.; Gryniukova, A.; Metelytsia, L. Ester-Functionalized Imidazolium- and Pyridinium-Based Ionic Liquids: Design, Synthesis and Cytotoxicity Evaluation. *Biointerface Res. Appl. Chem.* **2022**, *12* (3), 2905–2957.

(182) Zhu, P.; Kang, X.; Zhao, Y.; Latif Ullah and Zhang, H. Predicting the Toxicity of Ionic Liquids Toward Acetylcholinesterase Enzymes Using Novel QSAR Models. *Int. J. Mol. Sci.* **2019**, *20* (9), 2186.

(183) Wang, Z.; Song, Z.; Zhou, T. Machine Learning for Ionic Liquid Toxicity Prediction. *Processes (Basel)* **2021**, *9* (1), 65.

(184) Sadaghiyanfam, S.; Kamberaj, H.; Isler, Y. Enhanced Prediction of Ionic Liquid Toxicity Using a Meta-Ensemble Learning Framework with Data Augmentation. *Artificial Intelligence Chemistry* **2025**, *3* (1), 100087.

(185) Feng, H.; Jiajia, L.; Jian, Z. Prediction of Ionic Liquid Toxicity by Interpretable Machine Learning. *Chin. J. Chem. Eng.* **2025**, *84*, 201–210.

(186) Obaid, R. J.; Kotb, H.; Alsubaiyel, A. M.; Uddin, J.; Sani Sarjad, M.; Lutfor Rahman, M.; Ahmed, S. A. Novel and Accurate Mathematical Simulation of Various Models for Accurate Prediction of Surface Tension Parameters through Ionic Liquids. *Arab. J. Chem.* **2022**, *15* (11), 104228.

(187) Shojaeian, A.; Asadizadeh, M. Prediction of Surface Tension of the Binary Mixtures Containing Liquid Using Heuristic Approaches; an Input Parameters Investigation. *J. Mol. Liq.* **2020**, *298*, 111976.

(188) Setiawan, R.; Daneshfar, R.; Rezvanjou, O.; Ashoori, S.; Naseri, M. Surface Tension of Binary Mixtures Containing Environmentally Ionic Liquids: Insights from Artificial Intelligence. *Environ. Dev. Sustain.* **2021**, *23* (12), 17606–17627.

(189) Yalcin, D.; Le, T. C.; Drummond, C. J.; Greaves, T. L. Machine Learning Approaches for Further Developing the understanding of the Property Trends Observed in Protic Ionic Liquid Containing Solvents. *J. Phys. Chem. B* **2019**, *123* (18), 4085–4097.

(190) Bawazeer, S. Advanced Hybrid Machine Learning Based Modeling for Prediction of Properties of Ionic Liquids at Different Temperatures. *Sci. Rep.* **2025**, *15* (1), 21865.

(191) Deng, J.; Zhang, Y.; Jia, G. Surface Tension of Binary and Ternary Mixtures Mapping with ASP and UNIFAC Models Based on Machine Learning. *Phys. Fluids (1994)* **2023**, *35* (6), 062101.

(192) Zhu, W.; Zhang, R.; Liu, H.; Xin, L.; Zhong, J.; Zhang, H.; Qi, J.; Wang, Y.; Zhu, Z. Prediction of Ionic Liquid Surface Tension via a Generalized Structure-Surface Tension Relationship Model. *AIChE J.* **2024**, *70* (11), e18558.

(193) Paduszyński, K. Extensive Databases and Group Contribution QSPRs of Ionic Liquid Properties. 3: Surface Tension. *Ind. Eng. Chem. Res.* **2021**, *60* (15), 5705–5720.

(194) Alshahrani, S. M.; Uddin, J.; Yasmin, S.; Alshehri, S.; Alsubaiyel, A. M. Prediction of Novel Ionic Liquids' Surface Tension via Bagging KNN Predictive Model: Modeling and Simulation. *J. Mol. Liq.* **2022**, *368* (120748), 120748.

(195) Mohan, M.; Smith, M. D.; Demerdash, O.; Kidder, M. K.; Smith, J. C. Predictive Understanding of the Surface Tension and Velocity of Sound in Ionic Liquids Using Machine Learning. *J. Chem. Phys.* **2023**, *158* (21), 214502.

(196) Fu, Y.; Chen, Y.; Zhang, C.; Lei, Y.; Liu, X. Prediction Surface Tension of Ionic Liquid–Water Mixtures using a Hybrid Group Contribution and Artificial Neural Network Method. *Fluid Phase Equilib.* **2023**, *563*, 113571.

(197) Soleimani, R.; Saeedi Dehaghani, A. H.; Shoushtari, N. A.; Yaghoubi, P.; Bahadori, A. Toward an Intelligent Approach for Predicting Surface Tension of Binary Mixtures Containing Ionic Liquids. *Korean J. Chem. Eng.* **2018**, *35* (7), 1556–1569.

(198) ZareNezhad, B.; Aminian, A. Accurate Prediction of the Surface Tensions of Ionic–Amines–Water Mixtures Regarding CO_2 Recovery and sequestration Processes. *Chem. Eng. Commun.* **2016**, *203* (10), 1288–1297.

(199) Soleimani, R.; Saeedi Dehaghani, A. H. Insights into the Estimation of Surface Tensions of Mixtures on Designable Green Materials Using an Ensemble Learning. *Sci. Rep.* **2023**, *13* (1), 14145.

(200) Mohan, M.; Smith, M. D.; Demerdash, O. N.; Simmons, B. A.; Singh, S.; Kidder, M. K.; Smith, J. C. Quantum Chemistry-Driven Machine Learning Approach for the Prediction of the Surface Tension and Speed of Sound in Ionic Liquids. *ACS Sustain. Chem. Eng.* **2023**, *11* (20), 7809–7821.

(201) Benmouloud, W.; Si-Moussa, C.; Benkortbi, O. Machine Learning Approach for the Prediction of Surface tension of Binary Mixtures Containing Ionic Liquids Using σ-profile Descriptors. *Int. J. Quantum Chem.* **2023**, *123* (3), e27026.

(202) Abourehab, M. A. S.; Shawky, A. M.; Venkatesan, K.; Yasmin, S.; Alsubaiyel, A. M.; AboRas, K. M. Efficiency Development of Surface Tension for Different Ionic Liquids through Novel Model of Machine Learning Technique: Application of in-Thermal Engineering. *J. Mol. Liq.* **2022**, *367*, 120391.

(203) Járvás, G.; Kontos, J.; Babics, G.; Dallos, A. A Novel Method for the Surface Tension Estimation of Ionic Liquids Based on COSMO-RS Theory. *Fluid Phase Equilib.* **2018**, *468*, 9–17.

(204) Lazzús, J. A.; Cuturrufo, F.; Pulgar-Villarroel, G.; Salfate, I.; Vega, P. Estimating the Temperature-Dependent Surface Tension of Ionic Liquids Using a Neural Network-Based Group Contribution Method. *Ind. Eng. Chem. Res.* **2017**, *56* (23), 6869–6886.

(205) Sheikhshoaei, A. H.; Sanati, A. Interfacial Tension Modeling of Aqueous Ionic Liquids via Machine and Deep Learning. *Energy Fuels* **2025**, *39* (36), 17506-17521.

(206) Lashkarbolooki, M. Artificial Neural Network Modeling for Prediction of Binary Tension Containing Ionic Liquid. *Sep. Sci. Technol.* **2017**, *52* (8), 1454–1467.
